# Supplementary material for: Wet carbonate-promoted radical arylation of vinyl pinacolboronates with diaryliodonium salts yields substituted olefins
Source: Commun Chem. 2020 Jul 22;3:92. doi: 10.1038/s42004-020-00343-8 (PMC9814134; doi:10.1038/s42004-020-00343-8)
Supplement: Supplementary file 1 — Supplementary Information [file 42004_2020_343_MOESM1_ESM.pdf]

Supporting Information

Wet Carbonate-promoted Radical Arylation of Vinyl Pinacolboronates with Diaryliodonium Salts Yields Substituted Olefins

Chao Wu,[1+] Chongyang Zhao,[2+] Jun Zhou,[3] Han-Shi Hu,[2*] Jun Li,[2] Panpan Wu[3] and Chao Chen[1,3*]

[1]Key Laboratory of Bioorganic Phosphorus Chemistry & Chemical Biology (Ministry of Education), Department of Chemistry, and the Graduate School at Shenzhen, Tsinghua University, Beijing 100084, China. [2]Department of Chemistry & Key Laboratory of Organic Optoelectronics and Molecular Engineering of Ministry of Education, Tsinghua University, Beijing 100084, China. [3]Environmental Engineering, Wuyi University, Jiangmen, 529000, China; International Healthcare Innovation Institute (Jiangmen), Jiangmen, 529000, China [+] These authors contributed equally: Chao Wu, Chongyang Zhao. *Correspondence: chenchao01@mails.tsinghua.edu.cn; hshu@mail.tsinghua.edu.cn

Table of the contents:

General Comments……………………………………………………………………...……….…....2

General procedure synthesis of trans-arylvinylboronates……………..………………………….…....2

Optimization of the reaction conditions of step 1…………………....…………………………..….…4

General synthesis of substituted aryl olefins derivates………………..…………………………….….5

Optimization of the reaction conditions of step 2…………………....…………………………….….…5

Procedure for the preparation of compound **10**and **12**. ………………...……………………..…..…6

Controlled Experiments for the investigations on the mechanism…………………….………......7

Radical trapping experiments and EPR experiments……………..……………………….….......9

Density Functional Theory (DFT) calculations……………………………………………….….10

NMR spectra……………………….…………………………………………..…..……...…16

References…………………………………………………………………….……………….…….68

Supplementary Methods

General Comments

All of the reactions were conducted in dried Schlenk tubes with a Teflon-lined plug filled by N_2. All of the solvents were distilled before use according to the standard method. Column chromatography was performed on silica gel. ^{1}H NMR and ^{13}C NMR test were recorded at room temperature on a 300MHz or 400MHz spectrometer. Chemical shifts (δ) were given in ppm, referenced to the residual proton resonance of $CDCl_3$ (7.26 ppm), or to the carbon resonance of $CDCl_3$ (77.16 ppm). Multiple, doublet quartet, quartet, triplet, doublet, single was represented as m, dq, q, t, d, s, and coupling constants (J) were given in Hertz (Hz). Mass spectra were obtained using Bruker Esquire ion trap mass spectrometer in positive mode. The reaction progress was monitored by GC if applicable, using n-Dodecane as internal standard.

Diaryliodonium salts and vinyl pinacolboronic esters were prepared according to the literature methods unless the commercial available ones. [1-4]

General synthesis of trans-arylvinylboronates

1. Change the Hypervalent Iodonium

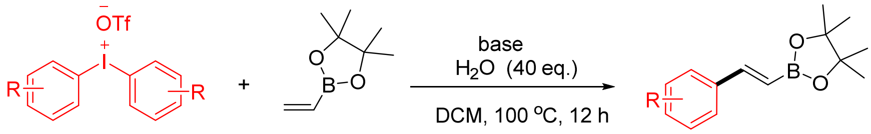

Mixture of diaryliodonium salt (0.15mmol, 1.0 eq.) and base [condition a: K_2CO_3 (1 eq.) condition b: Li_2CO_3 (2 eq.)] were added into a schlenk tube and then evacuated and recharged with N_2 for 3 times. After that, 1.0 ml DCM were added in, followed by vinyl pinacol boronic esters (0.30mmol, 51 µl) and pure water (6.0mmol, 100 µl). The tube and mixture were stirred at 100 °C for 12 h. After completion. The tube was cooled to room temperature, then NaCl aq. (10 ml) was added and the mixture was extracted with EtOAc (10 ml x 3), then dried by anhydrous Na_2SO_4. The mixture was evaporated then purified on silica gel (petroleum ether/ EtOAc=50:1) provided the corresponding product.

2. Change the Ester

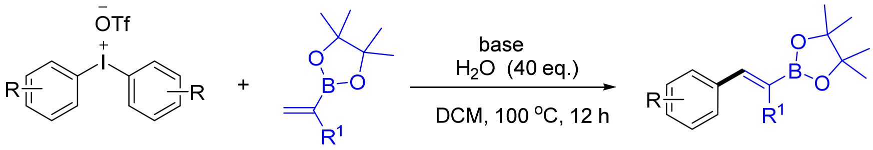

Mixture of diaryliodonium salt (0.15mmol, 1.0 eq.) and base [condition a: K_2CO_3 (1 eq.) condition b: Li_2CO_3 (2 eq.)] were added into a schlenk tube and then evacuated and recharged with N_2 for 3 times. After that, 1.0 ml DCM were added in, followed by vinyl pinacol boronic esters (0.30mmol, 51 μl) and pure water (6.0mmol, 100 μl). The tube and mixture were stirred at 100 ^{o}C for 12 h. After completion. The tube was cooled to room temperature, then NaCl aq. (10 ml) was added and the mixture was extracted with EtOAc (10 ml x 3), then dried by anhydrous Na_2SO_4. The mixture was evaporated then purified on silica gel (petroleum ether/ EtOAc=50:1) provided the corresponding product.

Optimization of the Reaction Conditions

Supplementary Table 1: Optimization of reaction conditions[a]

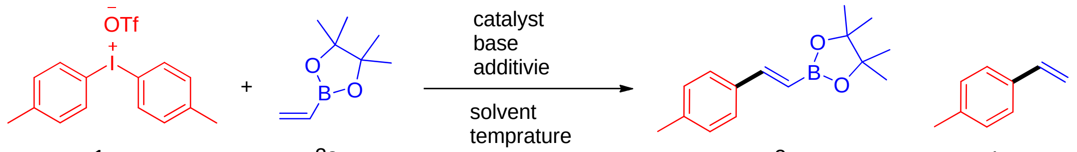

1a 2a 3a 4a

entry	catalyst	base (1 eq.)	addtive	solvent	temprature (^{o}C)	yield[b] (%) 3a	4a
1	CuCl	DIPEA	—	DCE	80	trace	<1
2	CuCl	K_2CO_3	—	DCE	80	trace	8
3	CuCl	K_2CO_3	TBAF[c]	DCE	80	3	2
4	CuCl	K_2CO_3	H_2O	DCE	80	62[d]	4
5	$Pd(OAc)_2$	K_2CO_3	H_2O	DCE	80	11	58
6	$Pd(PPh_3)_4$	K_2CO_3	H_2O	DCE	80	8	67
7	—	K_2CO_3	H_2O	DCE	80	64	<1
8	—	K_2CO_3	H_2O	DCM	80	81	<1
9	—	K_2CO_3	H_2O	PhMe	80	61	<1
10	—	K_2CO_3	H_2O	DCM	90	84	<1
**11**	—	**K_2CO_3**	**H_2O**	**DCM**	**100**	**89 (88)[e]**	**<1**
12	—	K_2CO_3	H_2O	CH_3OH	100	trace	0
13	—	K_2CO_3	H_2O	THF	100	trace	0
14	—	K_2CO_3	H_2O	DMF	100	trace	0
15	—	K_3PO_4	H_2O	DCM	100	51	<1
16	—	$NaHCO_3$	H_2O	DCM	100	78	<1
17	—	Li_2CO_3	H_2O	DCM	100	84 (82)[e]	<1
18	—	Ag_2CO_3	H_2O	DCM	100	NP[f]	<1
19	—	KH_2PO_4	H_2O	DCM	100	trace	<1
20	—	K_2CO_3 (2 eq.)	H_2O	DCM	100	43	<1
21	—	K_2CO_3	H_2O (10 eq.)	DCM	100	19	<1
22	—	K_2CO_3	H_2O (20 eq.)	DCM	100	62	<1
23	—	K_2CO_3	CH_3OH	DCM	100	NP[f]	NP[f]
24[g]	—	K_2CO_3	H_2O	DCM	100	23	0
25[h]	—	K_2CO_3	H_2O	DCM	100	19	0
26	—	—	H_2O	DCM	100	0	0

[a] Unless noted, reactions were performed with 1a (0.15 mmol), 2a (2.0 eq.), catalyst (10 mol%), and additive (40 eq.), in solvent (1 mL) at the temperature described. [b] Determined by GC analysis using n-dodecane as an internal standard. [c] 2.0 equiv of TBAF was used. [d] E/Z isomer of 3a was 2/1. [e] Isolated yield. [f] No product. [g]The counter anion was OTs. [h]The counter anion was OAc.

General synthesis of substituted aryl olefins derivates.

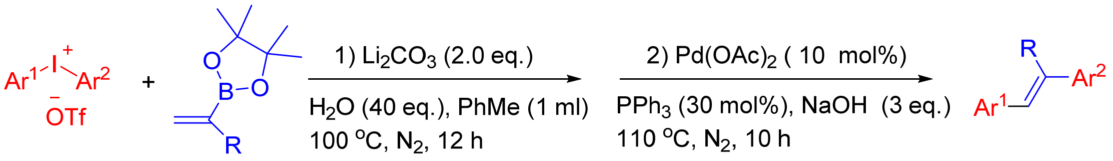

Mixture of diaryliodonium salt (0.15mmol, 1.0 eq.) and Li_2CO_3 (0.30 mmol, 2 eq.) were added into a schlenk tube and then evacuated and recharged with N_2 for 3 times. After that, 1.0 ml PhMe were added in, followed by vinyl pinacol boronic esters (0.165 mmol, 1.1 eq.) and pure water (6.0mmol, 100 μl). The tube and mixture were stirred at 100 °C for 12 h. After completion. The tube were cooled to room temperature, then $Pd(OAc)_2$(0.015mmol, 3.4 mg), PPh_3(0.045mmol, 11.8 mg), NaOH(0.45mmol, 18 mg) were in-situ added into the mixture of schlenk tube under the N_2 atmosphere, and then was stirred at 110 °C for 12 h. After completion. The tube were cooled to room temperature, then NaCl aq. (10 ml) was added and the mixture was extracted with EtOAc (10 ml x 3), then dried by anhydrous Na_2SO_4. The mixture was evaporated then purified on silica gel (petroleum ether) provided the corresponding product.

Supplementary Table 2 : Optimization of reaction conditions[a]

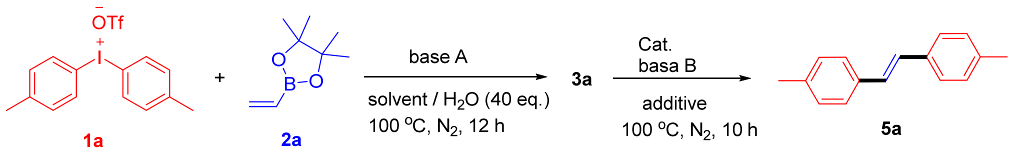

entry	base A	solvent	3a (yield[b]%)	cat.(10 mol%)	base B	addtive	conversion of 3a (%)[b]	yield[b] (%) 5a
1	K_2CO_3	DCM	84	$Pd(PPh_3)_4$	NaOH	—	17	13
2	K_2CO_3	PhMe	61	$Pd(PPh_3)_4$	NaOH	—	60	36
3	Li_2CO_3	PhMe	80	$Pd(PPh_3)_4$	NaOH	—	61	47
4	Li_2CO_3	DMF	trace	$Pd(PPh_3)_4$	NaOH	—	—	0
5	Li_2CO_3	PhMe	82	$Pd(PPh_3)_2Cl_2$	NaOH	—	87	54 (52)[c]
6	Li_2CO_3	PhMe	82	$Pd(OAc)_2$	NaOH	—	98	58
7	Li_2CO_3	PhMe	82	$Pd(OAc)_2$	NaOH	PPh_3	>99	71
8	Li_2CO_3	PhMe	82	$Pd(OAc)_2$	K_2CO_3	PPh_3	67	25
9	Li_2CO_3	PhMe	82	$Pd(OAc)_2$	Cs_2CO_3	PPh_3	80	31
10	Li_2CO_3	PhMe	82	$Pd(OAc)_2$	Li_2CO_3	PPh_3	64	16
**11**	**Li_2CO_3**	**PhMe**	**82**	**$Pd(OAc)_2$**	**NaOH**	**PPh_3**	**>99**	**82 (81)[c]**
12[d]	Li_2CO_3	PhMe	82	$Pd(OAc)_2$	NaOH	PPh_3	>99	72
13	Li_2CO_3	PhMe	82	$Pd(OAc)_2$	—	PPh_3	8	3
14	Li_2CO_3	PhMe	82	—	NaOH	PPh_3	0	0

[a] Unless noted, reactions were performed with 1a (0.15 mmol), 2a (1.1 eq.), H_2O(40 eq.), catalyst (10 mol%), base A (K_2CO_3,1.0 eq.) or (Li_2CO_3,2.0 eq.) , base B (3.0 eq.) and additive (0.3 eq.), in solvent (1 mL) at the temperature described. [b] Determined by GC analysis using n-dodecane as an internal standard. [c] Isolated yield. [d] 5mol% of $Pd(OAc)_2$ was used.

Procedure 1 for preparation of 10 from 5n:[5]

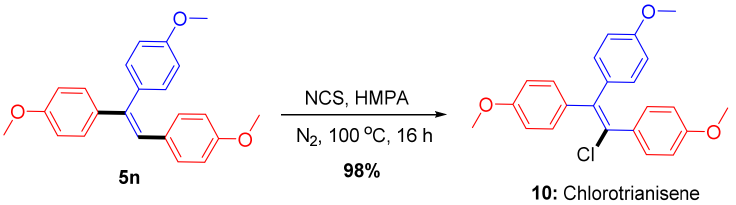

A sealed tube was charged with the mixture of 4,4',4''-(ethene-1,1,2-triyl) Tris(methoxybenzene)(5n) (0.4 mmol), N-Chlorosuccinimide (13.2 mmol, 1.726 g). The tube was evacuated and recharged with N_2 for 3 times. After HMPA (6.0 mL) was added, the tube was sealed and the mixture was allowed to stir at 100 °C for 16 h. After completion, the mixture was cooled to room temperature, the mixture was extracted with DCM (5 mL x 3), dried by anhydrous Na_2SO_4. The residue was subjected to silica gel column chromatography (PE/EA 50:1) to give the desired product **10** as a white solid (yield 98%).

Procedure 2 for preparation of 12 from 5m:

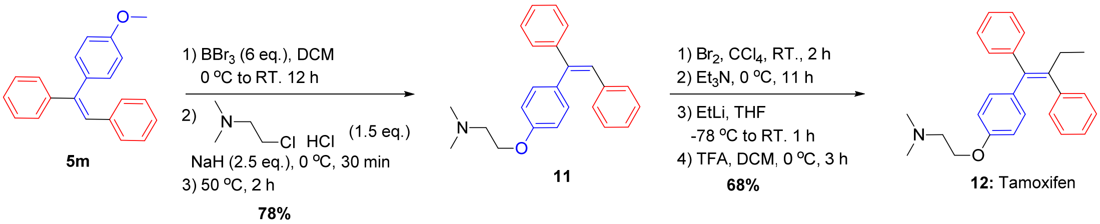

1): A sealed tube was charged with the mixture of (Z)-(1-(4-methoxyphenyl) Ethane -1,2-diyl) dibenzene (5m) (1 mmol), Boron Tribromide (6 mmol) was stirred for 1 h at 0 °C then warmed to room temperature and stirred for 12 h. After the complement, the NaH (2.5 eq.) in DCM (2ml) was added dropwise into the tube for 30 min. Then, the tube was heated to stir at 50 °C for 2 h. After completion, the mixture was cooled to room temperature, the mixture was extracted with EA (5 mL x 3), dried by anhydrous Na_2SO_4. The residue was subjected to silica gel column chromatography (PE/EA 10:1) to give the product **11** as a white solid (yield 78%).

2): According to the procedure described by Nunes,[6] a solution of bromine (575 mg, 3.6 mmol) in CH_2Cl_2 (10 mL) was added dropwise to a stirred solution of (E)-1-(4-phenoxy-N, N-dimethylethylamine)-1,2-diphenylethylene (1.03 g, 3 mmol) and Et_3N (1.20 g, 12 mmol) in CCl_4 (10 mL) at r.t. for 2 h. Then, the reaction mixture was kept overnight in the dark and washed with a saturated aqueous solution of $NaHSO_3$ to destroy the excess of bromine (until disappearance of the color). The organic layer was washed with a 10% aqueous KOH solution (20 mL) and dried over Na_2SO_4, and CCl_4 was evaporated to give solid mixture. Then, the mixture and THF (10 mL) was

added into a 50 mL, four-necked, round-bottomed flask equipped with a mechanical stirrer. Then, a solution of ethyllithium in ether (1M, 3.15 mmol, 3.15 mL, 1.05 eq.) was added dropwise at -78 °C in 1 h. After completion of the addition, the reaction mixture was stirred for 1 h at -78 °C then warmed to room temperature and stirred for 4 h. Solvents were removed in vacuo. The crude product and TFA (10 eq.) and CH_2Cl_2 (10 mL) at 0 °C in 3 h. Finally, The crude product was purified by chromatography on a silica gel column (eluent: petroleum ether / acetone / triethylamine (10:1:1)) yielding (Z)- tamoxifen **12** as a white solid (780 mg, 68%).

Controlled Experiments for the investigations on the mechanism

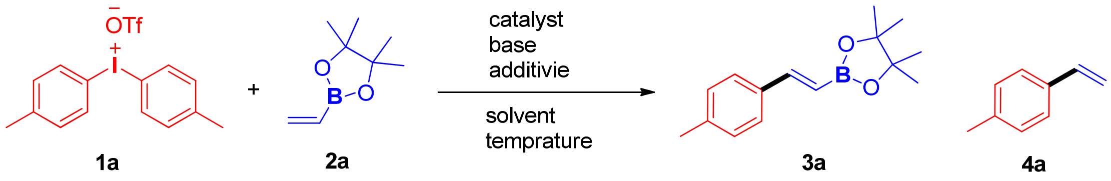

Mixture of diaryliodonium salt (0.15mmol, 1.0 eq.) and K_2CO_3 (n eq.) were added into a schlenk tube and then evacuated and recharged with N_2 for 3 times. After that, 1.0 ml DCM were added in, followed by vinyl pinacol boronic esters (0.30mmol, 51 μl) and pure water (m eq.). The tube and mixture were stirred at 100 °C. Then the mixture was detected in different reaction time periods.

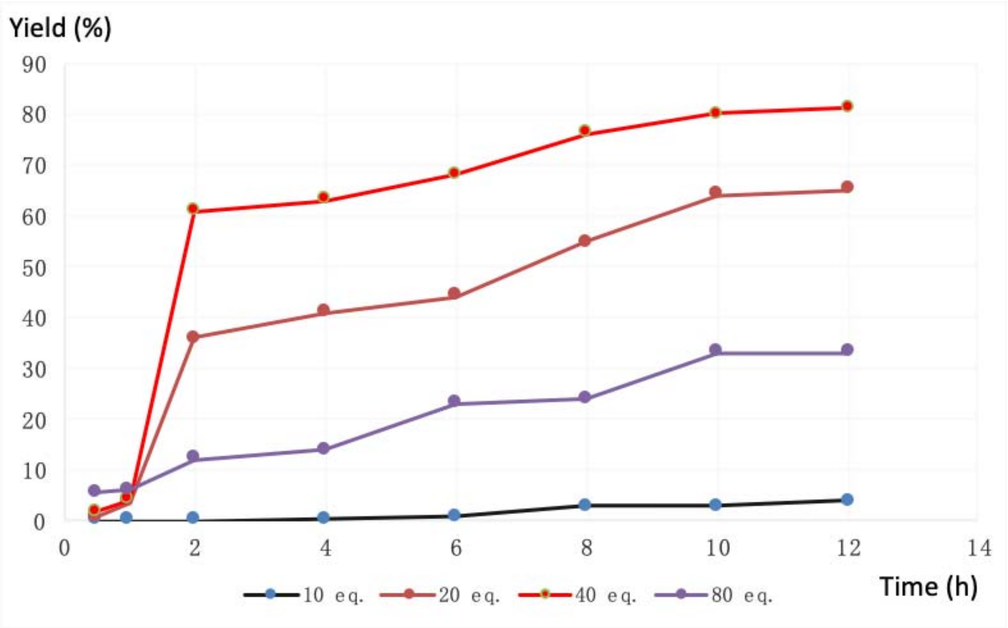

(1) **Supplementary Figure 1.** Monitoring the reaction of **1a** with **2a** under different equivalents of pure water by GC analysis using n-dodecane as internal standard.

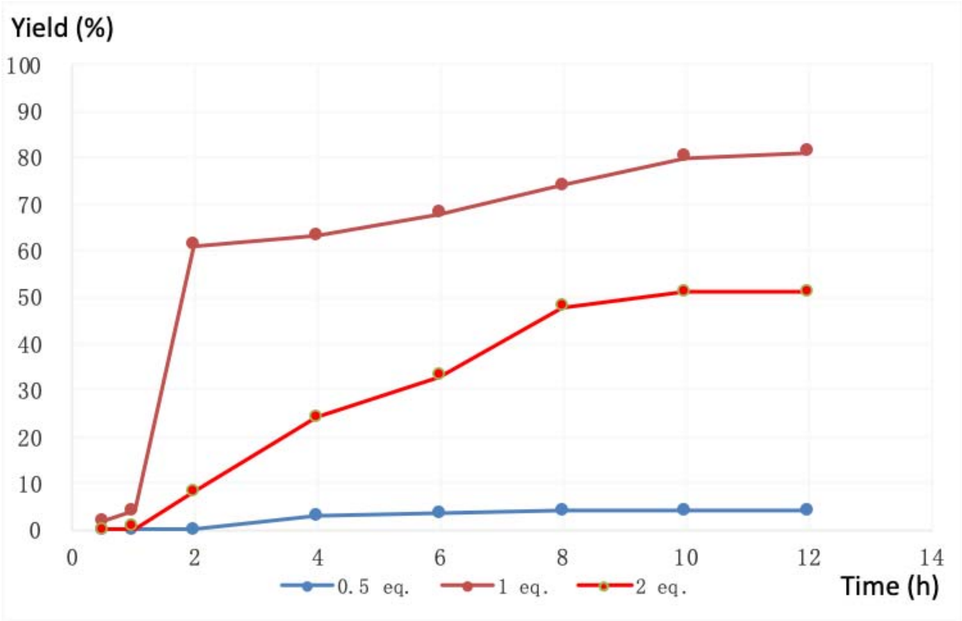

(2) **Supplementary Figure 2.** Monitoring the reaction of 1a with 2a under different equivalents of K_2CO_3 by GC analysis using n-dodecane as internal standard.

Radical trapping experiments

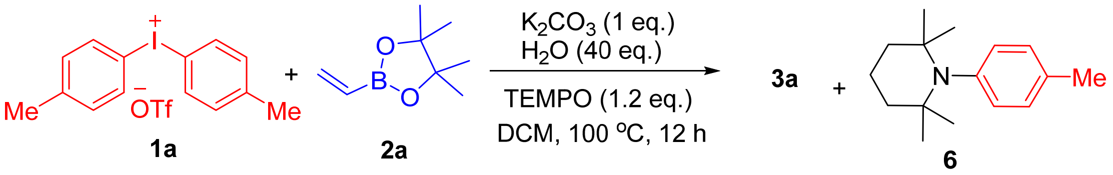

	3a	6
Standard condition	trace	21 %
The absence of **2a**	No	44 %
The absence of **K_2CO_3** or **H_2O**	trace	No

Standard condition: mixture of diaryliodonium salt (0.15mmol, 1.0 eq.) and K_2CO_3 (0.15mmol, 1.0 eq.) and TEMPO (0.18mmol, 1.2 eq.) were added into a schlenk tube and then evacuated and recharged with N_2 for 3 times. After that, 1.0 ml DCM were added in, followed by vinyl pinacol boronic esters (0.30mmol, 51 μl) and pure water (6.0mmol, 100 μl). The tube and mixture were stirred at 100 °C for 12 h. Then, the mixture was determined by GC with n-dodecane as internal standard. The trapping product **6** was detected by GC as well as the document (Yu' work) previously reported,[7]and the reason for the formation of this compound can be explained by Chen's work.[8]

EPR experiments

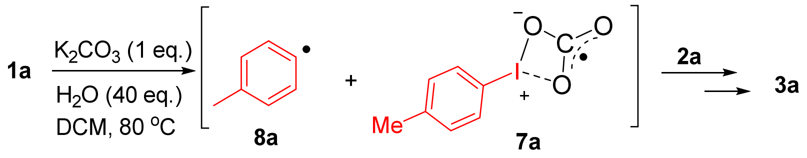

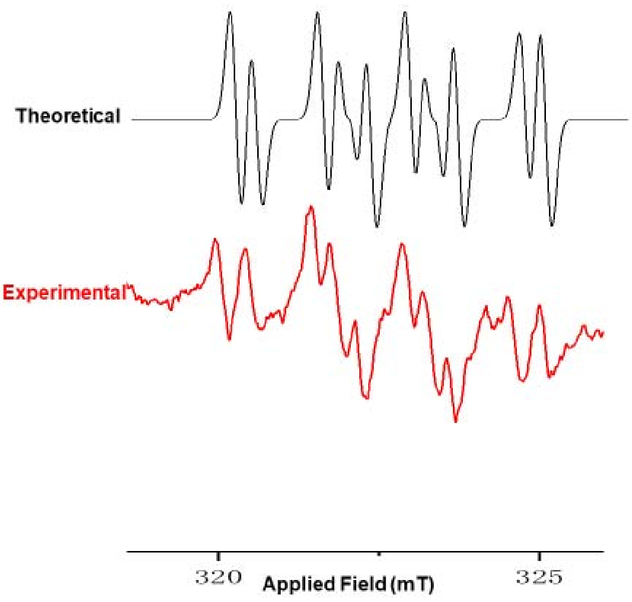

Supplementary Figure 3. Experimental EPR spectrum of the spin adducts **8a** (a_N=15.8 G, a_H=22.8 G), generated from the reaction of diphenyliodonium triflate (1a, 4.6 × 10^{-3} M) with and K_2CO_3 (1 eq.) and pure water (40 eq.) in the presence of the DMPO (5, 1.35 ×10^{-3} M) in DCM, and then the vinyl pinacol boronic esters (0.30mmol, 51 μl) was added to the mixture.

Density Functional Theory (DFT) calculations

Computational details: The density functional theory using B3LYP exchange-correlation functional [9-11] and Gaussian-09 software [12] were used for the calculations to explore the reaction mechanism. Ahlrichs' double-ζ polarized def2-SV(P)[13] basis set was employed. The geometry optimization was performed at the B3LYP/def2-SV(P) level. The characters of the stationary point (intermediates and transition state) were verified by the harmonic vibrational frequency analysis. The solvent effect was modeled by using the SMD continuum solvation model[14]. Inasmuch as the amount of dichloromethane was much larger than water added into the system, the modeled solvation used dichloromethane. The reported Gibbs free energies in this work were calculated at B3LYP/def2-TZVP[8] (Ahlrichs' triple-ζ multiple polarized basis set) level. The Gibbs free energy correction at the

experimental temperature 100°C was obtained from harmonic vibrational analysis in gas phase at the calculated stationary point.

Supplementary Note 1: The role of H_2O in the reaction: following which we have added more calculations by adding one H_2O molecule into complex **IV** and the results are shown in Figure 4 below. It shows that the addition of one H_2O molecule step is endothermic by 13.7 kcal/mol. The following step of proton transfer (PT) requires further 27.0kcal/mol free energy barrier. This H_2O molecule plays a role in the proton transfer between benzyl site and carbonate bonded to the boron atom. The total barrier including binding energy of the water-catalyzed proton abstraction is high as to 40.7kcal/mol. Therefore, we think the H_2O molecule does help to activate the reactions.

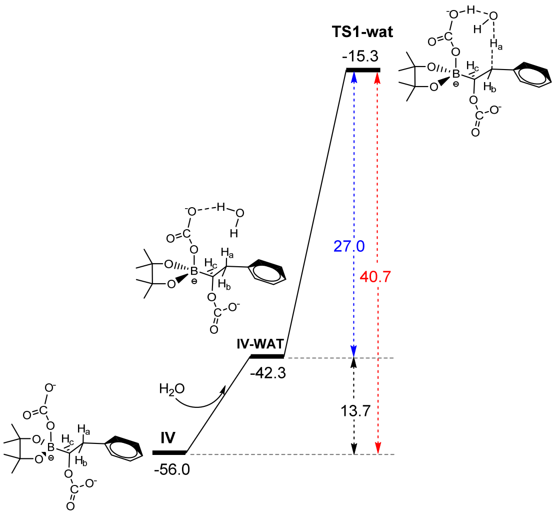

Supplementary Figure 4. The potential energy profile (in unit of kcal/mol) of the water-catalyzed proton transport between benzyl site and carbonate.

Besides, we have also computationally investigated the effect by increasing of the number of H_2O molecules and the results are listed in Figure 5 below. It shows that the binding energy and total barrier of the proton transfer increase along with increasing number of H_2O molecules, which tells that the H_2O molecule plays a role in hindering the reaction rather than stimulating it.

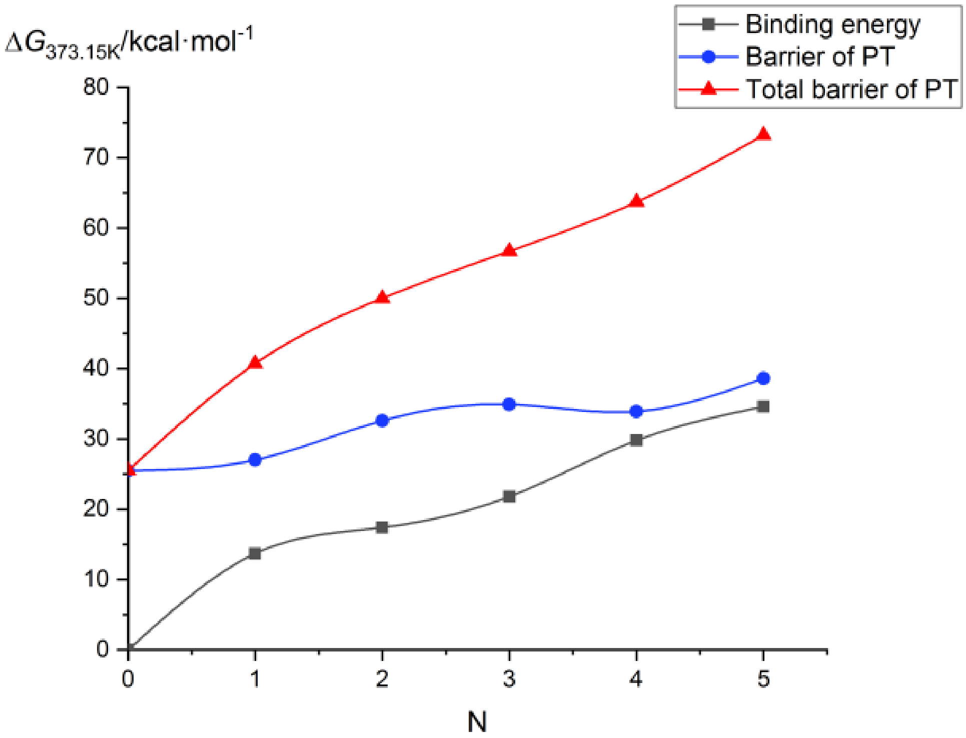

Supplementary Figure 5. The binding energy, barrier energy of proton transfer and the total barrier energy respectively with different number N of H_2O molecules.

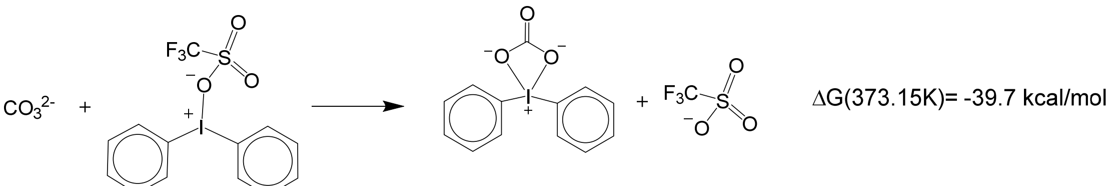

Supplementary Figure 6. The reaction Gibbs free energy for anion exchange of diaryliodonium salts from OTf⁻ to CO_3^{2-}.

In this system, the complex Ph-I$^+$-Ph$^-$X (X=K_2CO_3, CO_3^{2-}, OTf$^-$) decomposes to Ph-I$^+$-X· radical and phenyl radical endothermically. The Gibbs free energies required by the decomposition processes follow this order K_2CO_3 > OTf$^-$ > KCO_3^- > CO_3^{2-}.

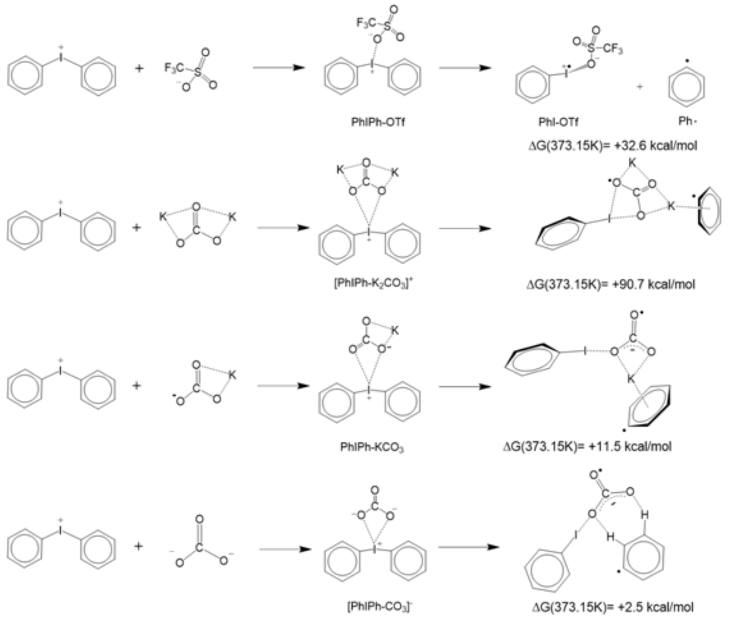

Supplementary Figure 7. The reaction Gibbs free energy change of the decomposition of $PhI^{+}Ph$-X ($X = OTf^{-}$, K_2CO_3 , KCO_3^{-} and CO_3^{2-})

As shown in Figure 7, the Gibbs free energy for the case $X = OTf^{-}$ and K_2CO_3 is around 90.7 kcal/mol, which is high that the decomposition cannot take place under the experimental condition .Although the Gibbs free energy for PhIPh-KCO_3 decomposition process is only about 11.5 kcal/mol in thermodynamics, the decomposition barrier is too high as shown in Figure 8 and it's hard to occur kinetically.

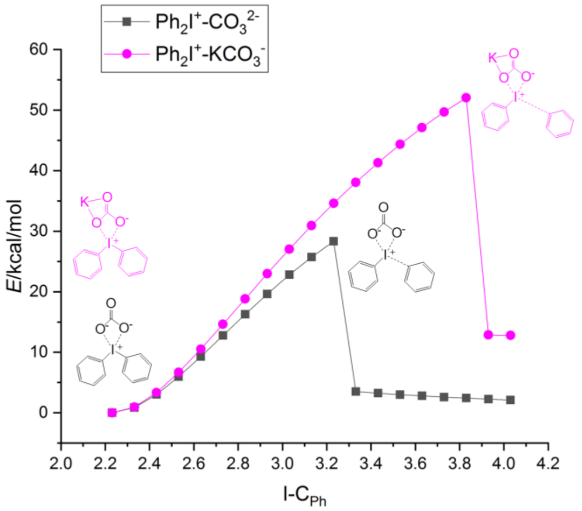

Supplementary Figure 8. The potential energy profile (in unit of kcal/mol) of the decomposition of Ph_2I^{+}-CO_3^{2-} and Ph_2I^{+}-KCO_3^{-} in the gas phase.

The decomposition of Ph_2I^{+}-CO_3^{2-} gives the halogen bonded complex radical pair PhI-$CO_3^{\cdot-}$-Ph·,

where the PhI and radical pair $CO_3^{\cdot -}$-Ph· are connected by one halogen bond, with the I-O bond length to be 2.73Å. The phenyl trapped by TEMPO comes from the further step of PhI-$CO_3^{\cdot -}$-Ph· decompostion, where phenyl radical bound to PhI-$CO_3^{\cdot -}$ via weak interaction between the hydrogen atom in phenyl radical and oxygen atoms in CO_3 moiety.

The Gibbs free energy for the case X = CO_3^{2-} coming from the ionization of K_2CO_3 by water is negative, explaining the reason why no reaction without water addition to the system. Figure 9 shows the Mulliken spin-density distributions for the atoms in the radical species.

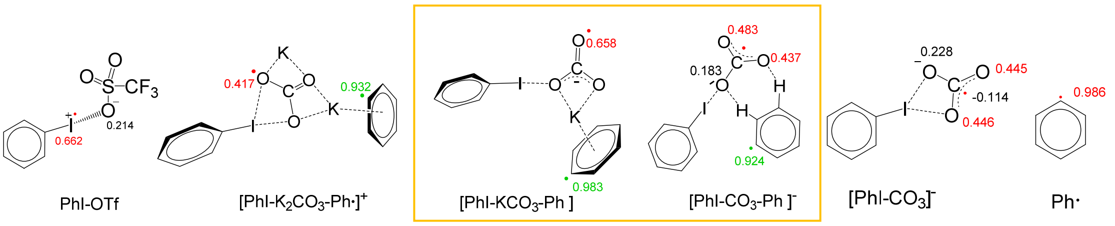

Supplementary Figure 9. The Mulliken spin-density distributions of the atoms in the decomposition product of PhI$^+$Ph-X (X = $^-$OTf, K_2CO_3, KCO_3^- and CO_3^{2-}) radical and phenyl radical. The values of Mulliken spin-density distributions less than 0.1 are not shown, the values in red are corresponding to the spin-density of α electron, and in green are corresponding to the spin-density of β electron.

As shown in Figure 10,the proposed mechanism starts from the combination of vinyl pinacol boronates and carbonate (potassium carbonate anion) to form the intermediate II (II-K), which is calculated to be exothermic by 7.4 kcal/mol(-0.9 kcal/mol). The phenyl radical attacking the =CH_2 group of II (II-K) to get the intermediate III (III-K) is calculated to be exothermic by 12.4 kcal/mol (14.4 kcal/mol).

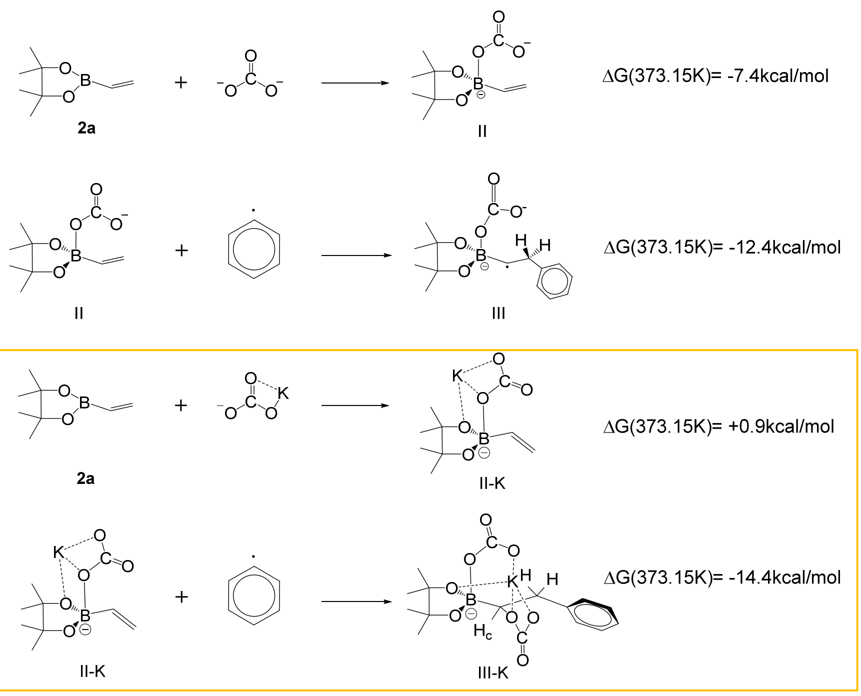

Supplementary Figure 10. The reaction of **2a** with carbonate and **II** with phenyl radical

As shown in Figure 11, we have also considered the situation that reaction starts from the binding of KCO_3^-, the incomplete solvated product of K_2CO_3 in water, to vinylboronate **2a** and the results are shown in Figure 5. The binding of KCO_3^- to 2a costs a small Gibbs free energy of 0.9kcal/mol. The addition of phenyl radical to $=CH_2$ group to get the intermediate III is calculated to be exothermic by 14.4kcal/mol. Then intermediate III reacts with PhI-CO_3^- radical to produce the intermediate IV with PhI. The next step is the rate-determining one with barrier of 36.1kcal/mol. The Ha-O bond length in TS1 is 1.00Å and O-C bond is 1.53Å, which indicates that TS1 is a late transition state. The barrier height of the rate-determining step in the case of K^+ is slightly higher than the total barrier height according to the energy difference between TS2 and IV in the case without K^+ by about 3.2kcal/mol. The departure of $[KCO_3\text{-}HCO_3]^{2-}$ cluster from boron atom need to overcome a free energy barrier of 22.7 kcal/mol. This barrier is much larger than the case without K^+. Therefore, from all these results, the favorable reaction pathway is the case without the participation of K^+.

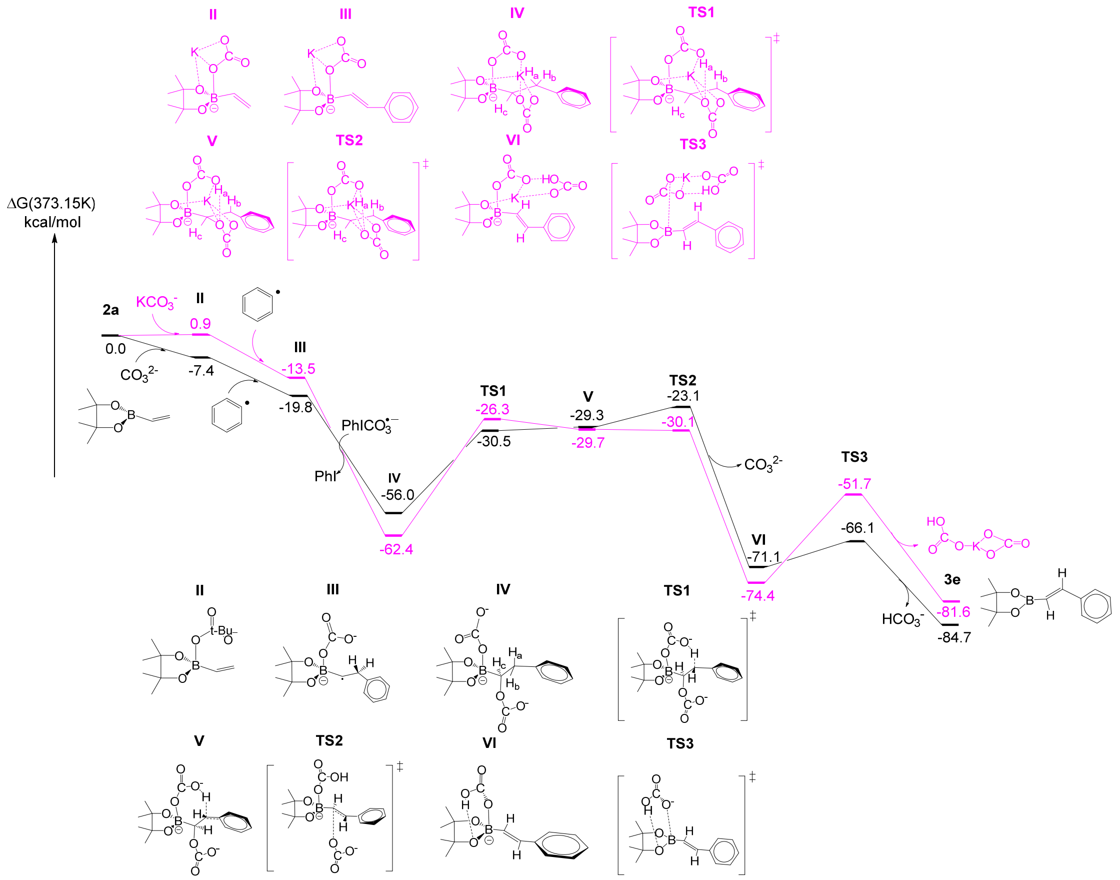

Supplementary Figure 11. The energy profile the reaction pathway level of theory with and without potassium ion in the reaction system

NMR spectra data of 3a-3w, 5a-5n and Chlorotrianisene 10 and Tamoxifen 12.

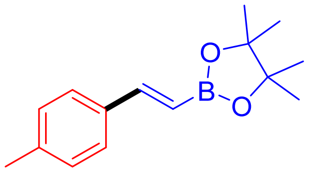

(E)-4,4,5,5-tetramethyl-2-(4-methylstyryl)-1,3,2-dioxaborolane (**3a**): white solid, yield: 88%, (PE/EA 50:1). **^{1}H NMR** (400 MHz, CHLOROFORM-D) δ 7.42 – 7.34 (m, 3H), 7.15 (d, *J* = 7.5 Hz, 2H), 6.12 (d, *J* = 18.4 Hz, 1H), 6.12 (d, *J* = 18.4 Hz, 1H), 2.35 (s, 3H), 1.32 (s, 12H). **^{13}C NMR** (101 MHz, CHLOROFORM-D) δ 149.6, 139.1, 134.9, 129.4, 127.1, 83.4, 77.5, 77.13, 76.8, 24.9, 21.4. **ESI-HRMS**: m/z calculated for $C_{15}H_{21}BO_2$ $[M+H]^+$: 245.1713; found: 245.197

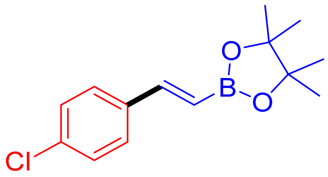

(E)-2-(4-chlorostyryl)-4,4,5,5-tetramethyl-1,3,2-dioxaborolane (**3b**): white solid, yield: 86%, (PE/EA 50:1). **^{1}H NMR** (400 MHz, CHLOROFORM-D) δ 7.40 (d, *J* = 7.5 Hz, 2H), 7.32 (t, *J* = 14.1 Hz, 3H), 6.13 (d, *J* = 18.5 Hz, 1H), 1.31 (s, 12H). **^{13}C NMR** (101 MHz, CHLOROFORM-D) δ 147.0, 134.9, 133.6, 127.8, 127.2, 82.4, 23.8. **ESI-HRMS**: m/z calculated for $C_{14}H_{18}BClO_2$ $[M+H]^+$: 265.1166; found: 265.1178.

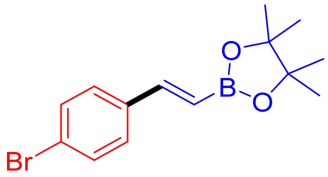

(E)-2-(4-bromostyryl)-4,4,5,5-tetramethyl-1,3,2-dioxaborolane (**3c**): white solid, yield: 80%, (PE/EA 50:1). **^{1}H NMR** (400 MHz, CHLOROFORM-D) δ 7.42 (d, *J* = 6.8 Hz, 2H), 7.30 (d, *J* = 6.8 Hz, 3H), 6.11 (d, *J* = 18.4 Hz, 1H), 1.27 (s, 12H). **^{13}C NMR** (101 MHz, CHLOROFORM-D) δ 148.18, 136.52, 131.87, 128.62, 123.01, 83.58, 24.93. **ESI-HRMS**: m/z calculated for $C_{14}H_{18}BBrO_2$ $[M+H]^+$: 309.0661; found: 309.0922.

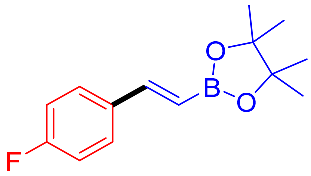

(E)-2-(4-fluorostyryl)-4,4,5,5-tetramethyl-1,3,2-dioxaborolane (**3d**): yellow solid, yield: 71%,

(PE/EA 50:1). **^{1}H NMR** (400 MHz, CHLOROFORM-D) δ 7.45 (s, 2H), 7.35 (d, *J* = 18.4 Hz, 1H), 7.02 (s, 2H), 6.07 (d, *J* = 18.3 Hz, 1H), 1.31 (s, 12H). **^{13}C NMR** (101 MHz, CHLOROFORM-D) δ 148.3, 128.90, 128.8, 115.8, 115.6, 83.5, 25.0. **ESI-HRMS**: m/z calculated for $C_{14}H_{18}BFO_2$ $[M+H]^+$: 249.1462; found: 249.1473.

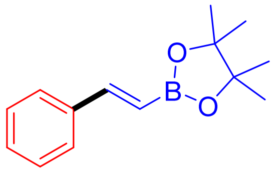

(E)-4,4,5,5-tetramethyl-2-styryl-1,3,2-dioxaborolane (**3e**): yellow solid, yield: 92%, (PE/EA 50:1). **^{1}H NMR** (400 MHz, CHLOROFORM-D) δ 7.52 – 7.48 (m, 2H), 7.41 (d, *J* = 18.4 Hz, 1H), 7.41 (d, *J* = 18.4 Hz, 1H), 7.37 – 7.31 (m, 2H), 7.32 – 7.27 (m, 1H), 6.18 (d, *J* = 18.5 Hz, 1H), 6.18 (d, *J* = 18.5 Hz, 1H), 1.32 (s, 12H). **^{13}C NMR** (101 MHz, CHLOROFORM-D) δ 149.6, 137.6, 129.0, 128.7, 127.2, 83.4, 24.9. **ESI-HRMS**: m/z calculated for $C_{14}H_{19}BO_2$ $[M+H]^+$: 231.1557; found: 231.1547.

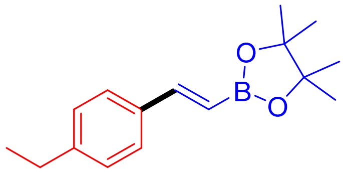

(E)-2-(4-ethylstyryl)-4,4,5,5-tetramethyl-1,3,2-dioxaborolane (**3f**): yellow solid, yield: 83%, (PE/EA 50:1). **^{1}H NMR** (400 MHz, CHLOROFORM-D) δ 7.41 (t, J = 11.9 Hz, 3H), 7.18 (d, J = 7.9 Hz, 2H), 6.13 (d, J = 18.4 Hz, 1H), 2.65 (q, J = 7.6 Hz, 2H), 1.32 (s, 12H), 1.24 (t, J = 7.6 Hz, 3H). **^{13}C NMR** (101 MHz, CHLOROFORM-D) δ 149.6, 145.4, 135.2, 128.2, 127.2, 83.4, 28.8, 24.9, 15.5. **ESI-HRMS**: m/z calculated for $C_{16}H_{23}BO_2$ $[M+H]^+$: 259.1869; found: 259.1937.

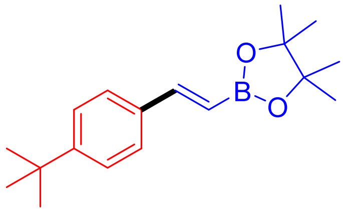

(E)-2-(4-(tert-butyl) styryl)-4,4,5,5-tetramethyl-1,3,2-dioxaborolane (**3g**): white solid, yield: 82%, (PE/EA 50:1). **^{1}H NMR** (400 MHz, CHLOROFORM-D) δ 7.40 (dd, J = 28.5, 7.7 Hz, 5H), 6.13 (d, J = 18.4 Hz, 1H), 1.32 (s, 21H). **^{13}C NMR** (101 MHz, CHLOROFORM-D) δ 152.3, 149.5, 134.9, 127.0, 125.6, 83.4, 34.8, 31.4, 25.0. **ESI-HRMS**: m/z calculated for $C_{18}H_{27}BO_2$ $[M+H]^+$: 287.2182; found: 287.2798.

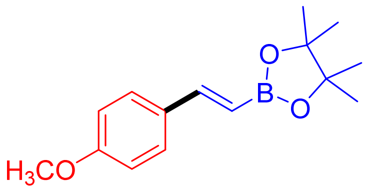

(E)-2-(4-methoxystyryl)-4,4,5,5-tetramethyl-1,3,2-dioxaborolane (**3h**): white solid, yield: 71%, (PE/EA 50:1). **^{1}H NMR** (400 MHz, CHLOROFORM-D) δ 7.46 – 7.41 (m, 2H), 7.35 (d, J = 18.4 Hz, 1H), 6.86 (d, J = 8.7 Hz, 2H), 6.01 (d, J = 18.4 Hz, 1H), 3.81 (s, 3H), 1.31 (s, 12H). **^{13}C NMR** (101 MHz, CHLOROFORM-D) δ 160.4, 149.2, 130.5, 128.6, 114.1, 83.3, 55.4, 24.9. **ESI-HRMS**: m/z calculated for $C_{15}H_{21}BO_3$ $[M+H]^+$: 261.1662; found: 261,1537.

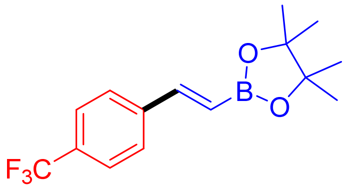

(E)-4,4,5,5-tetramethyl-2-(4-(trifluoromethyl)styryl)-1,3,2-dioxaborolane (**3i**): white solid, yield: 81%, (PE/EA 50:1). **^{1}H NMR** (400 MHz, CHLOROFORM-D) δ 7.57 (s, 4H), 7.40 (d, J = 18.4 Hz, 1H), 6.26 (d, J = 18.4 Hz, 1H), 1.31 (s, 12H). **^{13}C NMR** (101 MHz, CHLOROFORM-D) δ 147.8, 140.9, 130.6 (d, J = 32.3 Hz),127.3, 125.7 (d, J = 3.7 Hz), 124.2 (q, J = 272.1 Hz), 83.7, 25.0. **ESI-HRMS**: m/z calculated for $C_{15}H_{18}BF_3O_2$ $[M+H]^+$: 299.1430; found: 299.1462.

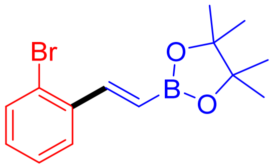

(E)-2-(2-bromostyryl)-4,4,5,5-tetramethyl-1,3,2-dioxaborolane (**3j**): white solid, yield: 91%, (PE/EA 50:1). **^{1}H NMR** (400 MHz, CHLOROFORM-D) δ 7.71 (d, J = 18.2 Hz, 1H), 7.61 (d, J = 7.9 Hz, 1H), 7.55 (d, J = 7.9 Hz, 1H), 7.31 – 7.26 (m, 1H), 7.14 (t, J = 7.6 Hz, 1H), 6.12 (d, J = 18.2 Hz, 1H), 1.32 (s, 12H). **^{13}C NMR** (101 MHz, CHLOROFORM-D) δ 147.7, 137.5, 133.2, 130.0, 127.6, 127.4, 124.5, 83.6, 25.0. **ESI-HRMS**: m/z calculated for $C_{14}H_{18}BBrO_2$ $[M+H]^+$: 309.0661; found: 309.0924..

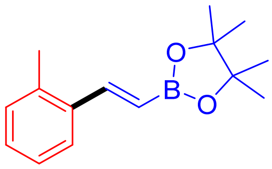

(E)-4,4,5,5-tetramethyl-2-(2-methylstyryl)-1,3,2-dioxaborolane (**3k**): white solid, yield: 72%, (PE/EA 50:1). **^{1}H NMR** (400 MHz, CHLOROFORM-D)δ 7.65 (d, J = 18.3 Hz, 1H), 7.56 (d, J = 8.5 Hz, 1H), 7.19 (s, 3H), 6.09 (d, J = 18.2 Hz, 1H), 2.42 (s, 3H), 1.32 (s, 12H). **^{13}C NMR** (101 MHz, CHLOROFORM-D) δ 147.3, 136.9, 136.4, 130.5, 128.7, 126.2, 125.9, 83.4, 25.0, 20.0. **ESI-HRMS**: m/z calcd for $C_{15}H_{21}BO_2$ $[M+H]^+$: 245.1713; found: 245.1972.

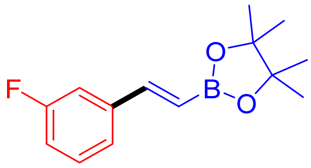

(E)-2-(3-fluorostyryl)-4,4,5,5-tetramethyl-1,3,2-dioxaborolane (**3l**): white solid, yield: 81%, (PE/EA 50:1). **^{1}H NMR** (400 MHz, CHLOROFORM-D) δ 7.32 (dd, J = 18.2, 12.8 Hz, 2H), 7.26 (d, J = 6.9 Hz, 2H), 7.17 (d, J = 10.1 Hz, 1H), 6.98 (s, 1H), 6.16 (d, J = 18.4 Hz, 1H), 1.31 (s, 12H). **^{13}C NMR** (101 MHz, CHLOROFORM-D) δ 148.2, 140.0 (d, J = 7.8 Hz), 130.2 (d, J = 8.4 Hz), 123.1, 115.8 (d, J = 21.3 Hz), 113.4 (d, J = 21.5 Hz), 83.7, 25.0. **ESI-HRMS**: m/z calculated for $C_{15}H_{21}BO_3$ $[M+H]^+$: 249.1462; found: 249.1474.

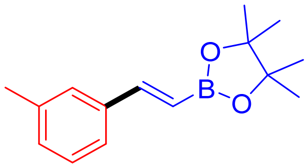

(E)-4,4,5,5-tetramethyl-2-(3-methylstyryl)-1,3,2-dioxaborolane (**3m**): white solid, yield: 82%, (PE/EA 50:1). **^{1}H NMR** (400 MHz, CHLOROFORM-D)δ 7.38 (d, J = 18.4 Hz, 1H), 7.31 (s, 2H), 7.23 (t, J = 7.8 Hz, 1H), 7.11 (d, J = 7.3 Hz, 1H), 6.16 (d, J = 18.4 Hz, 1H), 2.35 (s, 3H), 1.32 (s, 12H). **^{13}C NMR** (101 MHz, CHLOROFORM-D) δ 149.8, 138.2, 137.6, 129.8, 128.6, 127.9, 124.4, 83.4, 25.0, 21.5. **ESI-HRMS**: m/z calculated for $C_{15}H_{21}BO_2$ $[M+H]^+$: 245.1713; found: 245.1974.

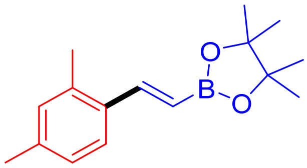

(E)-2-(2,4-dimethylstyryl)-4,4,5,5-tetramethyl-1,3,2-dioxaborolane (**3n**): white solid, yield: 82%, (PE/EA 50:1). **^{1}H NMR** (400 MHz, CHLOROFORM-D) δ 7.62 (d, J = 18.3 Hz, 1H), 7.47 (d, J = 7.8 Hz, 1H), 7.01 – 6.95 (m, 2H), 6.04 (d, J = 18.3 Hz, 1H), 2.39 (s, 3H), 2.31 (s, 3H), 1.32 (s, 12H). **^{13}C NMR** (101 MHz, CHLOROFORM

-D) δ 147.1, 138.7, 136.4, 134.0, 131.3, 127.0, 125.9, 83.4, 25.0, 21.3, 19.9. **ESI-HRMS**: m/z calculated for $C_{16}H_{23}BO_2$ $[M+H]^+$: 259.1869; found: 259.1934.

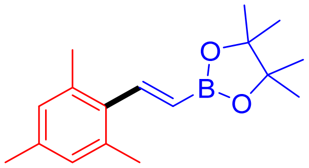

(E)-4,4,5,5-tetramethyl-2-(2,4,6-trimethylstyryl)-1,3,2-dioxaborolane (**3o**): white solid, yield: 55%, (PE/EA 50:1). **^{1}H NMR** (400 MHz, CHLOROFORM-D) δ 7.45 (d, J = 18.9 Hz, 1H), 6.86 (s, 2H), 5.69 (d, J = 18.8 Hz, 1H), 2.30 (s, 6H), 2.27 (s, 3H), 1.33 (s, 12H). **^{13}C NMR** (101 MHz, CHLOROFORM-D) δ 148.7, 136.8, 136.0, 135.3, 128.9, 83.4, 25.0, 21.1, 21.1. **ESI-HRMS**: m/z calculated for $C_{17}H_{25}BO_2$ $[M+H]^+$: 273.2026; found: 273.2087.

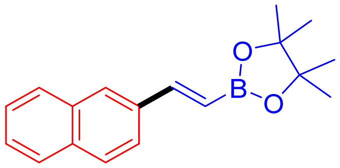

(E)-4,4,5,5-tetramethyl-2-(2-(naphthalen-2-yl)vinyl)-1,3,2-dioxaborolane (**3p**): yellow solid, yield: 55%, (PE/EA 50:1). **^{1}H NMR** (400 MHz, CHLOROFORM-D) δ 7.86 – 7.79 (m, 4H), 7.71 (dd, J = 8.7, 1.2 Hz, 1H), 7.58 (d, J = 18.4 Hz, 1H), 7.49 – 7.44 (m, 2H), 6.30 (d, J = 18.4 Hz, 1H), 1.34 (s, 12H). **^{13}C NMR** (101 MHz, CHLOROFORM-D) δ 149.7, 135.1, 133.9, 133.6, 128.9, 128.6, 128.4, 128.2, 127.8, 126.6, 126.4, 123.5, 83.6, 24.8. **ESI-HRMS**: m/z calculated for $C_{18}H_{21}BO_2$ [M+H]+:281.1713; found: 281.1774

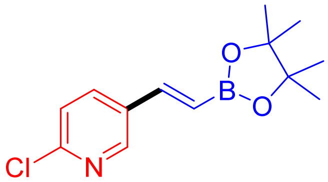

(E)-2-chloro-5-(2-(4,4,5,5-tetramethyl-1,3,2-dioxaborolan-2-yl)vinyl)pyridine (**3q**): brown solid, yield: 51%, (PE/EA 50:1). **^{1}H NMR** (400 MHz, CHLOROFORM-D) δ 8.43 (d, J = 2.3 Hz, 1H), 7.76 (dd, J = 8.3, 2.4 Hz, 1H), 7.31 (dd, J = 13.4, 9.4 Hz, 2H), 6.22 (d, J = 18.7 Hz, 1H), 1.31 (s, 12H). **^{13}C NMR** (101 MHz, CHLOROFORM-D) δ 151.4, 149.0, 144.2, 135.9, 132.1, 124.4, 83.9, 24.9. **ESI-HRMS**: m/z calculated for $C_{13}H_{17}BClNO_2$ $[M+H]^+$:266.1119; found: 266.1152.

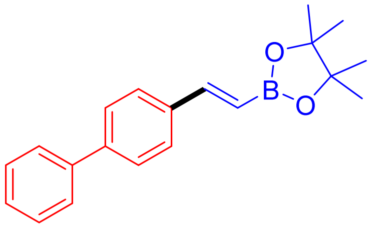

(E)-2-(2-([1,1'-biphenyl]-4-yl)vinyl)-4,4,5,5-tetramethyl-1,3,2-dioxaborolane (**3r**): yellow solid, yield: 57%, (PE/EA 50:1). **^{1}H NMR** (400 MHz, CHLOROFORM-D) δ 7.65 – 7.57 (m, 6H), 7.46 (dd, J = 16.0, 8.5 Hz, 3H), 7.36 (t, J = 7.3 Hz, 1H), 6.24 (d, J = 18.4 Hz, 1H), 1.35 (s, 12H). **^{13}C NMR** (101 MHz, CHLOROFORM-D) δ 149.1, 141.7, 140.7, 136.6, 128.9, 127.6, 127.6, 127.4, 127.1, 83.5, 24.9. **ESI-HRMS**: m/z calculated for $C_{20}H_{23}BO_2$ $[M+H]^+$:307.1869; found: 307.1883.

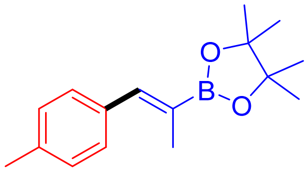

(Z)-4,4,5,5-tetramethyl-2-(1-(p-tolyl)prop-1-en-2-yl)-1,3,2-dioxaborolane (**3t**): white solid, yield: 93%, (PE/EA 50:1). **^{1}H NMR** (400 MHz, CHLOROFORM-D)δ 7.29 (d, J = 6.8 Hz, 2H), 7.21 (s, 1H), 7.15 (d, J = 7.1 Hz, 2H), 2.34 (s, 3H), 1.99 (s, 3H), 1.31 (s, 12H). **^{13}C NMR** (101 MHz, CHLOROFORM-D) δ 142.5, 137.0, 135.3, 129.6, 128.9, 83.6, 25.0, 21.4, 16.1. **ESI-HRMS**: m/z calculated for $C_{16}H_{23}BO_2$ $[M+H]^+$:259.1869; found: 259.1846.

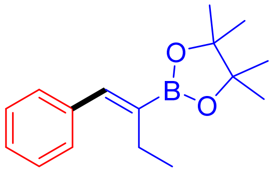

(Z)-4,4,5,5-tetramethyl-2-(1-phenylbut-1-en-2-yl)-1,3,2-dioxaborolane (**3u**): white solid, yield: 86%, (PE/EA 50:1). **^{1}H NMR** (400 MHz, CHLOROFORM-D)δ 7.34 (d, J = 4.5 Hz, 3H), 7.27 – 7.21 (m, 2H), 2.41 (q, J = 7.4 Hz, 2H), 1.33 (s, 12H), 1.12 (t, J = 7.5 Hz, 3H). **^{13}C NMR** (101 MHz, CHLOROFORM-D) δ 141.5, 138.0, 129.1,128.2, 127.1, 83.5, 24.9, 22.8, 14.8. **ESI-HRMS**: m/z calculated for $C_{16}H_{23}BO_2$ $[M+H]^+$:

259.1869; found: 259.1885.

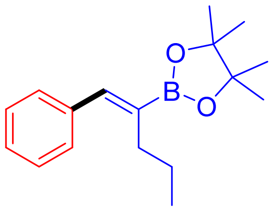

(Z)-4,4,5,5-tetramethyl-2-(1-phenylpent-1-en-2-yl)-1,3,2-dioxaborolane (**3v**): white solid, yield: 78%, (PE/EA 50:1). **^{1}H NMR** (400 MHz, CHLOROFORM-D) δ 7.34 – 7.32 (m, 3H), 7.27 – 7.21 (m, 2H), 2.40 – 2.31 (m, 2H), 1.56 – 1.46 (m, 2H), 1.31 (s, 12H), 0.92 (t, J = 7.4 Hz, 3H). **^{13}C NMR** (101 MHz, CHLOROFORM-D) δ 142.0, 138.2, 129.1, 128.2, 127.1, 83.5, 31.7, 24.9, 23.4, 14.4. ESI-HRMS: m/z calculated for $C_{17}H_{25}BO_2$ $[M+H]^+$:273.2026; found: 273.2058.

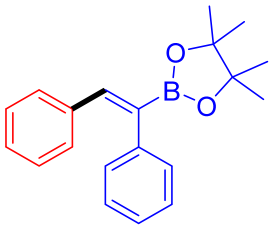

(Z)-2-(1,2-diphenylvinyl)-4,4,5,5-tetramethyl-1,3,2-dioxaborolane (**3w**): white solid, yield: 27%, (PE/EA 50:1). **^{1}H NMR** (400 MHz, CHLOROFORM-D) δ 7.37 (s, 1H), 7.25 (ddd, J = 7.4, 4.4, 1.4 Hz, 2H), 7.20 – 7.15 (m, 3H), 7.09 (dd, J = 6.0, 2.5 Hz, 3H), 7.06 – 7.02 (m, 2H), 1.29 (s, 12H). **^{13}C NMR** (101 MHz, CHLOROFORM-

D)δ 143.3, 140.5, 137.1, 130.0, 128.9, 128.3, 127.9, 127.7, 126.4, 83.8, 24.9.

ESI-HRMS: m/z calculated for $C_{20}H_{23}BO_2$ $[M+H]^+$:307.1869; found: 307.1937.

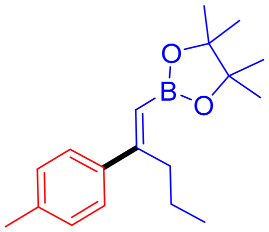

(E)-4,4,5,5-tetramethyl-2-(2-(p-tolyl) pent-1-en-1-yl)-1,3,2-dioxaborolane (**3x**): yellow solid, yield: 41%, (PE/EA 50:1). **^{1}H NMR** (400 MHz, CHLOROFORM-D) δ 7.35 (d, J = 8.1 Hz, 2H), 7.12 (d, J = 8.1 Hz, 2H), 5.63 (s, 1H), 2.92 – 2.83 (m, 2H), 2.34 (s, 3H), 1.40 (dd, J = 14.8, 7.4 Hz, 2H), 1.30 (s, 12H), 0.87 (t, J = 7.3 Hz, 3H).

^{13}C NMR (101 MHz, CHLOROFORM-D) δ 163.1, 140.4, 137.7, 129.0, 126.4, 82.9, 35.1, 25.0, 23.0, 21.3, 13.8. **ESI-HRMS**: m/z calculated for $C_{18}H_{27}BO_2$ $[M+H]^+$:

287.2182; found: 287.2243.

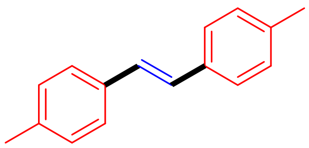

(E)-1,2-di-p-tolylethene (**5a**): white solid, yield: 81%, (PE).**^{1}H NMR** (400 MHz, CHLOROFORM-D) δ 7.43 (d, *J* = 7.9 Hz, 4H), 7.19 (d, *J* = 7.8 Hz, 4H), 7.07 (s, 2H), 2.39 (s, 6H). **^{13}C NMR** (101 MHz, CHLOROFORM-D) δ 137.4, 134.9, 129.5, 127.8, 126.4, 21.4. **ESI-HRMS**: m/z calculated for $C_{16}H_{16}$ $[M+H]^+$:209.1332; found:

209.1367.

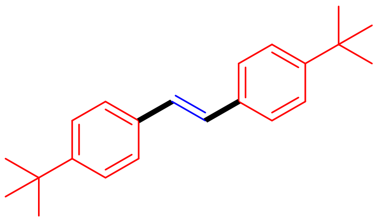

(E)-1,2-bis(4-(tert-butyl) phenyl) ethene (**5b**): white solid, yield: 64%, (PE). **^{1}H NMR** (400 MHz, CHLOROFORM-D) δ 7.46 (d, J = 8.1 Hz, 4H), 7.39 (d, J = 8.3 Hz, 4H), 7.07 (s, 2H), 1.34 (s, 24H). **^{13}C NMR** (101 MHz, CHLOROFORM-D) δ 150.7, 134.9, 127.9, 126.3, 125.7, 34.8, 31.5. **ESI-HRMS**: m/z calculated for $C_{22}H_{28}$ $[M+H]^+$:293.2269; found: 293.2281.

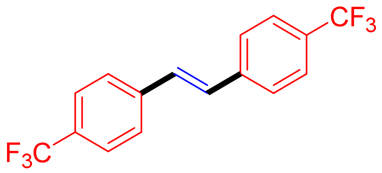

(E)-1,2-bis(4-(trifluoromethyl) phenyl) ethene (**5c**): yellow solid, yield: 41%, (PE).

^{1}H NMR (400 MHz, CHLOROFORM-D) δ 7.65 – 7.58 (m, 8H), 7.19 (d, J = 2.7 Hz, 2H). **^{13}C NMR** (101 MHz, CHLOROFORM-D) δ 140.2, 129.7, 127.0, 125.9, 125.9, 124.3 (d, J = 271.9 Hz).

ESI-HRMS: m/z calculated for $C_{16}H_{10}$ F_6 $[M+H]^+$:317.0765; found: 317.0797.

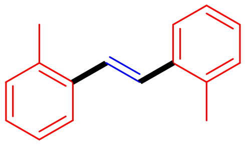

(E)-1,2-di-o-tolylethene (**5d**): white solid, yield: 78%, (PE). **^{1}H NMR** (400 MHz, CHLOROFORM-D) δ 7.63 (d, *J* = 7.1 Hz, 2H), 7.25 (dt, *J* = 10.4, 3.9 Hz, 8H), 2.46 (s, 6H). **^{13}C NMR** (101 MHz, CHLOROFORM-D) δ 136.9, 136.0, 130.5, 128.1, 127.7, 126.3, 125.7, 20.1. **ESI-HRMS**: m/z calculated for $C_{16}H_{16}$ $[M+H]^+$:209.1332; found: 209.1359.

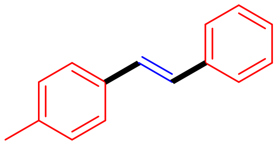

(E)-1-methyl-4-styrylbenzene (**5e**): white solid, yield: 81%, (PE). **^{1}H NMR** (400 MHz, CHLOROFORM-D) δ 7.53 (d, J = 7.5 Hz, 2H), 7.44 (d, J = 8.1 Hz, 2H), 7.37 (t, J = 7.6 Hz, 2H), 7.29 – 7.24 (m, 1H), 7.19 (d, J = 8.0 Hz, 2H), 7.10 (d, J = 2.5 Hz, 2H), 2.41 – 2.36 (m, 3H). **^{13}C NMR** (101 MHz, CHLOROFORM-D) δ 137.6, 134.7, 129.5, 128.8, 128.7, 127.8, 127.5, 126.6, 126.5, 21.4. **ESI-HRMS**: m/z calculated for $C_{15}H_{14}$ $[M+H]^{+}$:195.1174; found: 195.1189.

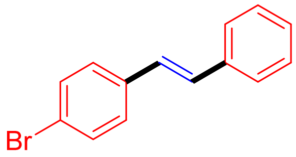

(E)-1-bromo-4-styrylbenzene (**5f**): white solid, yield: 61%, (PE). **^{1}H NMR** (400 MHz, CHLOROFORM-D) δ 7.52 (d, J = 1.3 Hz, 1H), 7.49 (t, J = 2.3 Hz, 2H), 7.47 (d, J = 1.9 Hz, 1H), 7.40 – 7.34 (m, 4H), 7.31 – 7.26 (m, 1H), 7.10 (d, J = 16.3 Hz, 1H), 7.03 (d, J = 16.4 Hz, 1H). **^{13}C NMR** (101 MHz, CHLOROFORM-D) δ 137.1, 136.4, 131.9, 129.6, 128.9, 128.1, 128.1, 127.5, 126.7, 121.5. **ESI-HRMS**: m/z calculated for $C_{14}H_{11}$ Br $[M+H]^{+}$:259.0122; found: 259.0146.

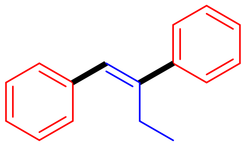

(E)-but-1-ene-1,2-diyldibenzene (**5g**): white solid, yield: 72%, (PE). **^{1}H NMR** (400 MHz, CHLOROFORM-D) δ 7.58 (d, J = 1.5 Hz, 1H), 7.56 (s, 1H), 7.48 – 7.41 (m, 6H), 7.41 – 7.37 (m, 1H), 7.35 (dd, J = 6.9, 2.1 Hz, 1H), 6.80 (s, 1H), 2.85 (q, J = 7.5 Hz, 2H), 1.17 (t, J = 7.5 Hz, 3H). **^{13}C NMR** (101 MHz, CHLOROFORM-D) δ 144.6, 142.8, 138.4, 128.9, 128.5, 128.4, 127.7, 127.3, 126.8, 126.7, 23.4, 13.6. **ESI-HRMS**: m/z calculated for $C_{16}H_{16}$ $[M+H]^{+}$:209.1332; found: 209.1354.

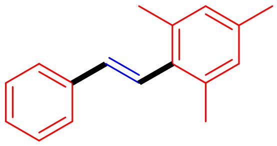

(E)-1,3,5-trimethyl-2-styrylbenzene (**5h**): white solid, yield: 58%, (PE). **^{1}H NMR** (400 MHz, CHLOROFORM-D) δ 7.54 (d, J = 7.3 Hz, 2H), 7.41 (t, J = 7.5 Hz, 2H), 7.30 (dd, J = 10.5, 4.0 Hz, 1H), 7.15 (d, J = 16.6 Hz, 1H), 6.95 (s, 2H), 6.63 (d, J = 16.6 Hz, 1H), 2.39 (s, 6H), 2.34 (s, 3H). **^{13}C NMR**

(101 MHz, CHLOROFORM-D) δ 137.9, 136.4, 136.3, 134.1, 133.8, 128.9, 128.8, 127.6, 127.1, 126.4, 21.1, 21.1.

ESI-HRMS: m/z calculated for $C_{17}H_{18}$ $[M+H]^+$:223.1487; found: 223.1486.

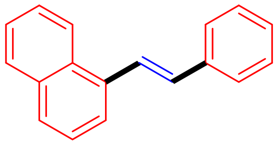

(E)-1-styrylnaphthalene (**5i**): white solid, yield: 80%, (PE). **^{1}H NMR** (400 MHz, CHLOROFORM-D) δ 8.24 (d, J = 8.0 Hz, 1H), 7.94 – 7.87 (m, 2H), 7.82 (d, J = 8.2 Hz, 1H), 7.77 (d, J = 7.1 Hz, 1H), 7.63 (d, J = 7.4 Hz, 2H), 7.57 – 7.49 (m, 3H), 7.42 (t, J = 7.6 Hz, 2H), 7.33 (d, J = 7.4 Hz, 1H), 7.17 (d, J = 16.0 Hz, 1H). **^{13}C NMR** (101 MHz, CHLOROFORM-D) δ 137.8, 135.2, 133.9, 131.9, 131.5, 128.9, 128.8, 128.2, 127.9, 126.8, 126.2, 126.0, 126.0, 125.8, 123.9, 123.8. **ESI-HRMS**: m/z calculated for $C_{18}H_{14}$ $[M+H]^+$:231.1174; found: 231.1182.

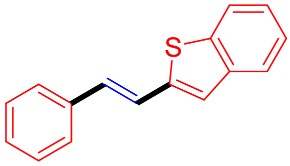

(E)-2-styrylbenzo[b]thiophene (**5j**): yellow solid, yield: 63%, (PE /EA =20:1). **^{1}H NMR** (400 MHz, CHLOROFORM-D) δ 7.80 – 7.76 (m, 1H), 7.70 (dd, J = 6.3, 2.6 Hz, 1H), 7.52 (d, J = 7.4 Hz, 2H), 7.37 (dd, J = 13.2, 5.4 Hz, 3H), 7.34 – 7.27 (m, 4H), 7.00 (d, J = 16.0 Hz, 1H). **^{13}C NMR** (101 MHz, CHLOROFORM-D) δ 143.1, 140.4139.1, 136.8, 132.2, 131.1, 128.9, 128.2, 126.7, 124.9, 124.7, 123.6, 123.4, 122.5, 122.4. **ESI-HRMS**: m/z calculated for $C_{16}H_{12}$ $[M+H]^+$:237.0738; found: 237.0742.

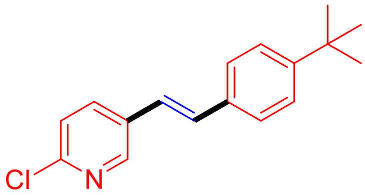

(E)-5-(4-(tert-butyl)styryl)-2-chloropyridine (**5k**): dark green solid, yield: 51%, (PE /EA =10:1). **^{1}H NMR** (400 MHz, CHLOROFORM-D) δ 8.46 (d, J = 2.2 Hz, 1H), 7.80 (dd, J = 8.3, 2.4 Hz, 1H), 7.46 (d, J = 8.3 Hz, 2H), 7.41 (d, J = 8.4 Hz, 2H), 7.30 (d, J = 8.3 Hz, 1H), 7.14 (dd, J = 16.5, 7.4 Hz, 1H), 6.99 (d, J = 16.4 Hz, 1H), 1.34 (s, 9H).

^{13}C NMR (101 MHz, CHLOROFORM-D) δ 151.9, 149.8, 148.2, 135.3, 133.7, 132.4, 131.5, 126.6, 125.9, 124.3, 122.7, 34.9, 31.4. **ESI-HRMS**: m/z calculated for $C_{17}H_{18}$ ClN $[M+H]^+$:272.1206; found: 272.1241.

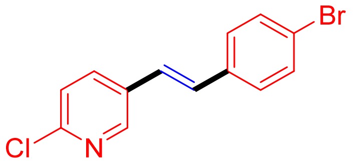

(E)-5-(4-bromostyryl)-2-chloropyridine (**5l**): dark green solid, yield: 54%, (PE /EA =10:1). **^{1}H NMR** (400 MHz, CHLOROFORM-D) δ 8.46 (d, J = 2.4 Hz, 1H), 7.78 (dd, J = 8.3, 2.5 Hz, 1H), 7.50 (d, J = 8.5 Hz, 2H), 7.37 (d, J = 8.5 Hz, 2H), 7.31 (d, J = 8.3 Hz, 1H), 7.09 – 6.97 (m, 2H). **^{13}C NMR** (101 MHz, CHLOROFORM-D) δ 150.3, 148.3, 135.4, 135.4, 132.1, 131.8, 130.3, 128.2, 124.4, 124.3, 122.5. **ESI-HRMS**: m/z calculated for $C_{13}H_9BrClN$ $[M+H]^+$:293.9685; found: 293.9673.

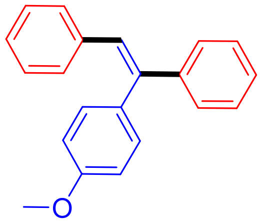

(Z)-(1-(4-methoxyphenyl)ethene-1,2-diyl)dibenzene (**5m**): white solid, yield: 37%, (PE). **^{1}H NMR** (400 MHz, CHLOROFORM-D) δ 7.41 (dd, J = 5.2, 1.8 Hz, 3H), 7.36 (d, J = 8.8 Hz, 2H), 7.34 – 7.29 (m, 2H), 7.24 – 7.16 (m, 3H), 7.14 – 7.09 (m, 2H), 7.00 (s, 1H), 6.94 (d, J = 8.9 Hz, 2H), 3.88 (s, 3H). **^{13}C NMR** (101 MHz, CHLOROFORM-D) δ 159.3, 142.2, 140.7, 137.7, 136.1, 130.5, 129.5, 128.9, 128.7, 128.0, 127.4, 126.6, 126.6, 113.7, 55.4. **ESI-HRMS**: m/z calculated for $C_{21}H_{18}O$ $[M+H]^+$:287.1436; found: 287.1447.

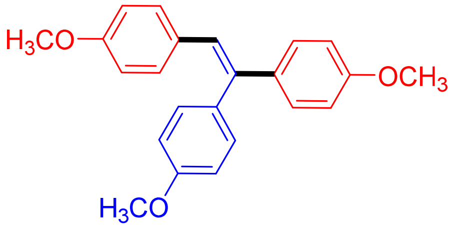

4,4',4''-(ethene-1,1,2-triyl)tris(methoxybenzene) (**5n**): white solid, yield: 41%, (PE). **^{1}H NMR** (400 MHz, CHLOROFORM-D) δ 7.28 (d, *J* = 8.7 Hz, 2H), 7.17 (d, *J* = 8.6 Hz, 2H), 7.01 (d, *J* = 8.7 Hz, 2H), 6.91 (d, *J* = 8.6 Hz, 2H), 6.87 (d, *J* = 8.7 Hz, 2H), 6.83 (s, 1H), 6.72 (d, *J* = 8.7 Hz, 2H), 3.86 (s, 3H), 3.83 (s, 3H), 3.77 (s, 3H). **^{13}C NMR** (101 MHz, CHLOROFORM-D) δ 159.1, 158.9, 158.2, 139.9, 136.7, 133.1, 131.7, 130.7, 130.6, 128.7, 125.8, 114.1, 113.6, 113.5, 55.4, 55.3, 55.2. **ESI-HRMS**: m/z calculated for $C_{23}H_{22}O_3$ $[M+H]^+$:347.1647; found: 347.1683.

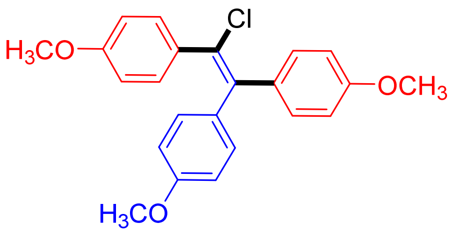

4,4',4''-(2-chloroethene-1,1,2-triyl) tris(methoxybenzene) Chlorotrianisene (**10**): white solid, yield: 98%, (PE/EA=50:1)

^{1}H NMR (400 MHz, CHLOROFORM-D) δ 7.20 (d, J = 8.7 Hz, 2H), 7.16 (d, J = 8.8 Hz, 2H), 6.79 (dd, J = 8.7, 3.2 Hz, 4H), 6.62 (d, J = 8.8 Hz, 2H), 6.54 (d, J = 8.7 Hz, 2H), 3.72 (s, 3H), 3.66 (s, 3H), 3.62 (s, 3H).**^{13}C NMR** (101 MHz, CHLOROFORM-D) δ 159.0, 158.8, 158.5, 138.6, 134.9, 134.1, 132.3, 132.0, 131.5, 131.4, 128.4, 113.5, 113.4, 55.3, 55.2. **DEPT-135: ^{13}C NMR** (101 MHz, CHLOROFORM-D) δ 132.0, 131.5, 131.4, 113.5, 113.4, 55.3, 55.2. **ESI-HRMS**: m/z calculated for $C_{23}H_{21}ClO_3$ $[M+H]^+$:381.1259; found: 381.1271.

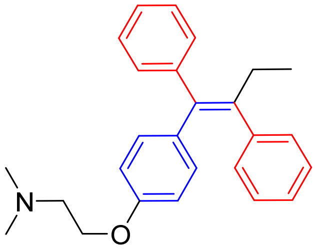

(Z)-2-(4-(1,2-diphenylbut-1-en-1-yl) phenoxy)-N, N-dimethylethan-1-amine

Tamoxifen (**12**): white solid, yield: 68%, (petroleum ether / acetone / triethylamine =10:1:1)

^{1}H NMR (400 MHz, CHLOROFORM-D) δ 7.40 – 7.32 (m, 2H), 7.26 (ddd, J = 6.6, 5.2, 1.5 Hz, 3H), 7.15 (tdd, J = 5.5, 4.6, 1.5 Hz, 5H), 6.84 – 6.74 (m, 2H), 6.63 – 6.53 (m, 2H), 3.97 – 3.90 (m, 2H), 2.68 – 2.62 (m, 2H), 2.46 (dd, J = 14.8, 7.4 Hz, 2H), 2.28 (s, 6H), 1.01 – 0.90 (m, 3H). **^{13}C NMR** (101 MHz, CHLOROFORM-D) δ 156.9, 143.9, 142.5, 141.4, 138.4, 135.6, 131.9, 129.8, 129.6, 128.2, 128.0, 126.6, 126.1, 113.5, 65.8, 58.4, 46.1, 29.1, 13.7. **ESI-HRMS**: m/z calculated for $C_{26}H_{29}NO$ $[M+H]^+$: 372.2329, found: 372.2335.

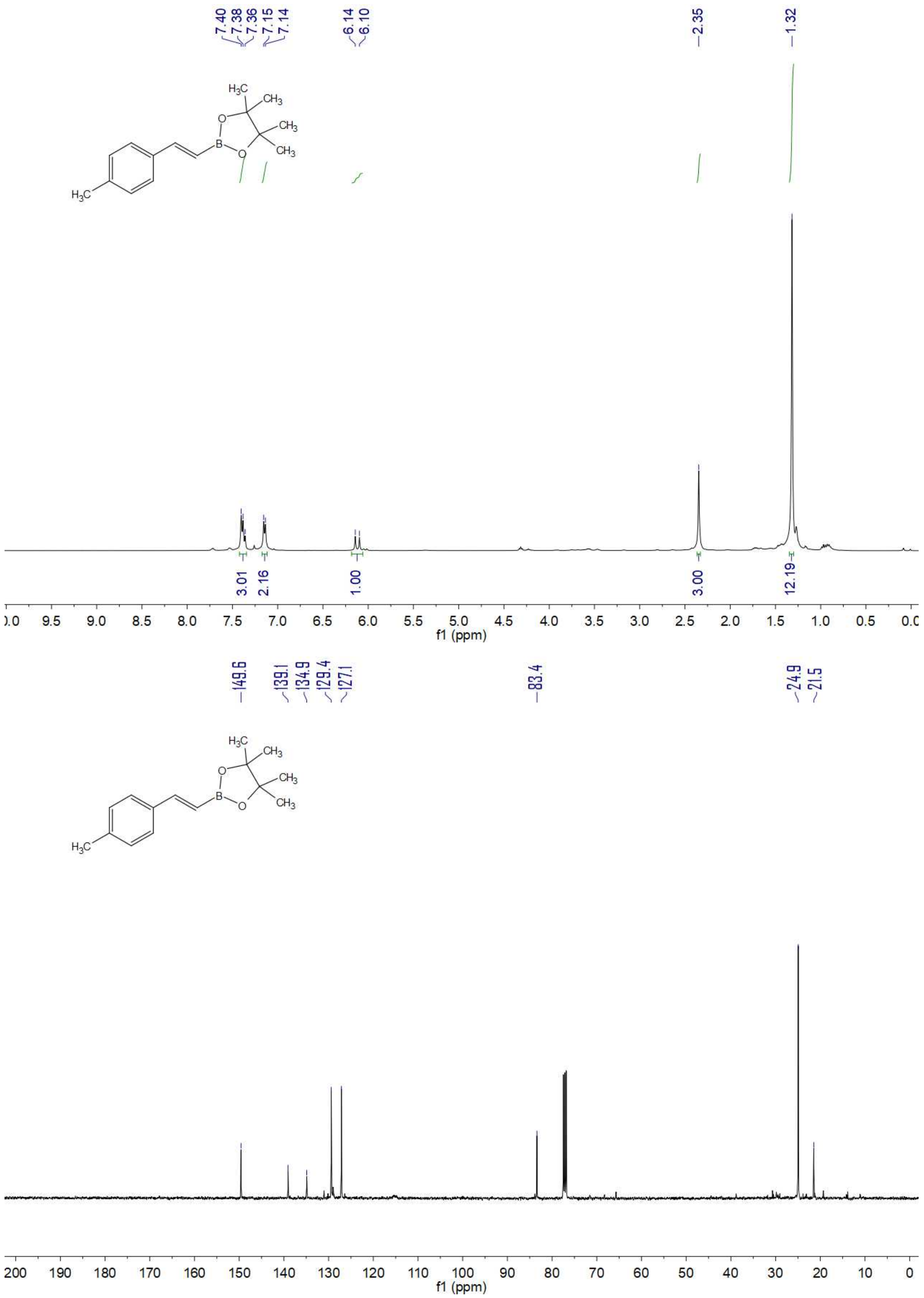

Supplementary Figure 12. **3a:** ^{1}H NMR (400 MHz, $CDCl_3$) (up) and ^{13}C NMR (101 MHz, $CDCl_3$) (down)

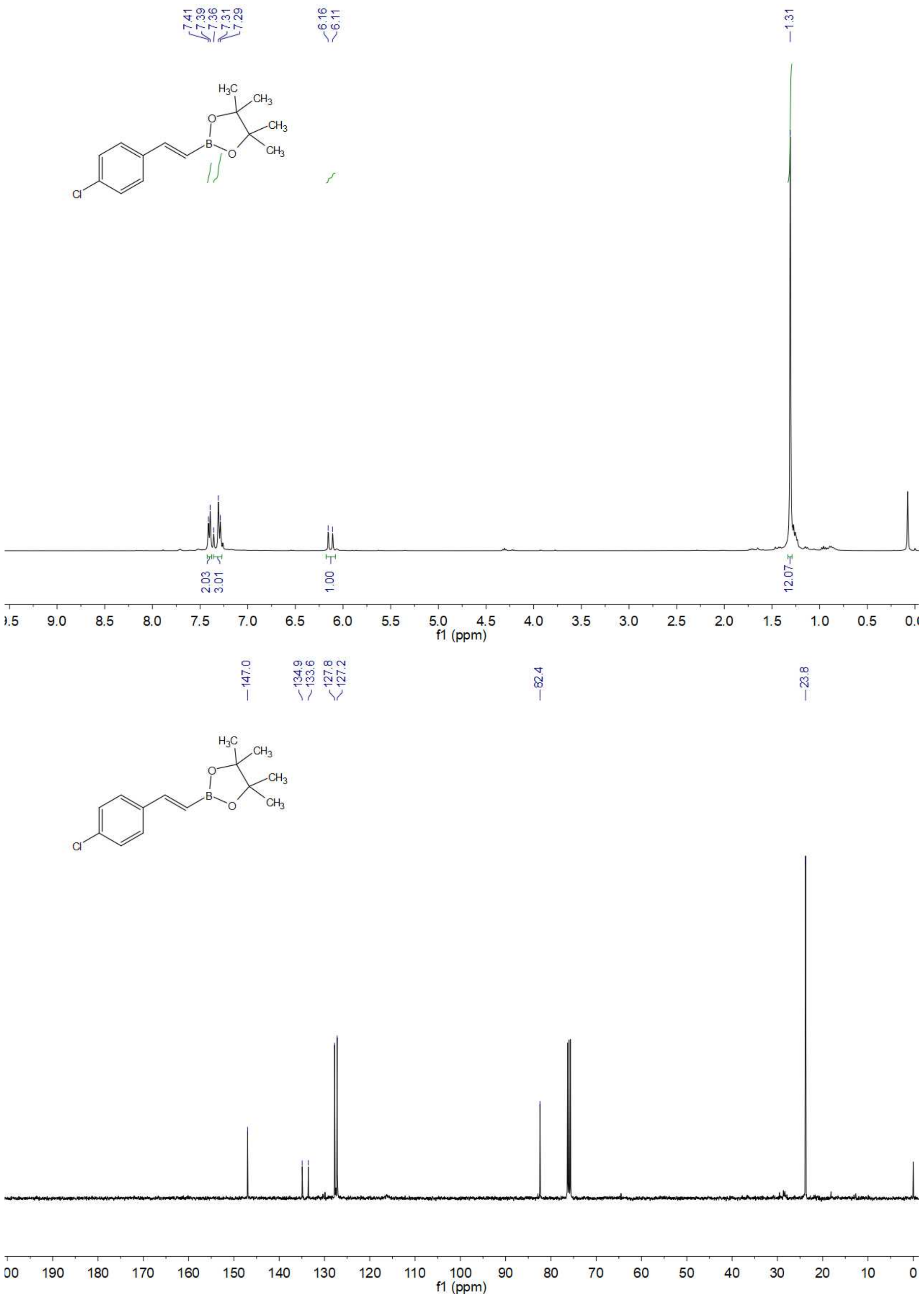

Supplementary Figure 13. **3b:** ^{1}H NMR (400 MHz, $CDCl_3$) (up) and ^{13}C NMR (101 MHz, $CDCl_3$) (down)

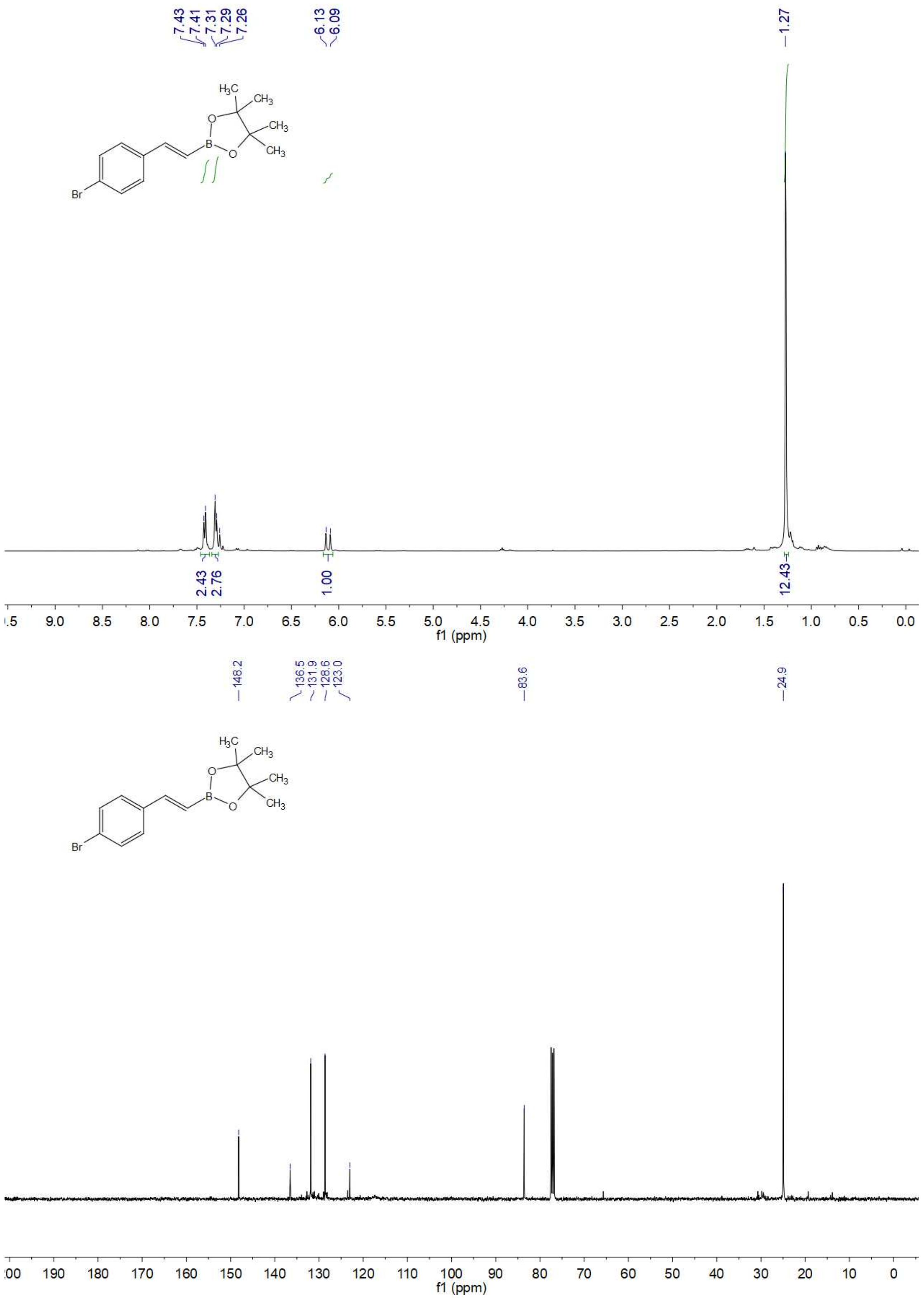

Supplementary Figure 14. **3c:** ^{1}H NMR (400 MHz, $CDCl_3$) (up) and ^{13}C NMR (101 MHz, $CDCl_3$) (down)

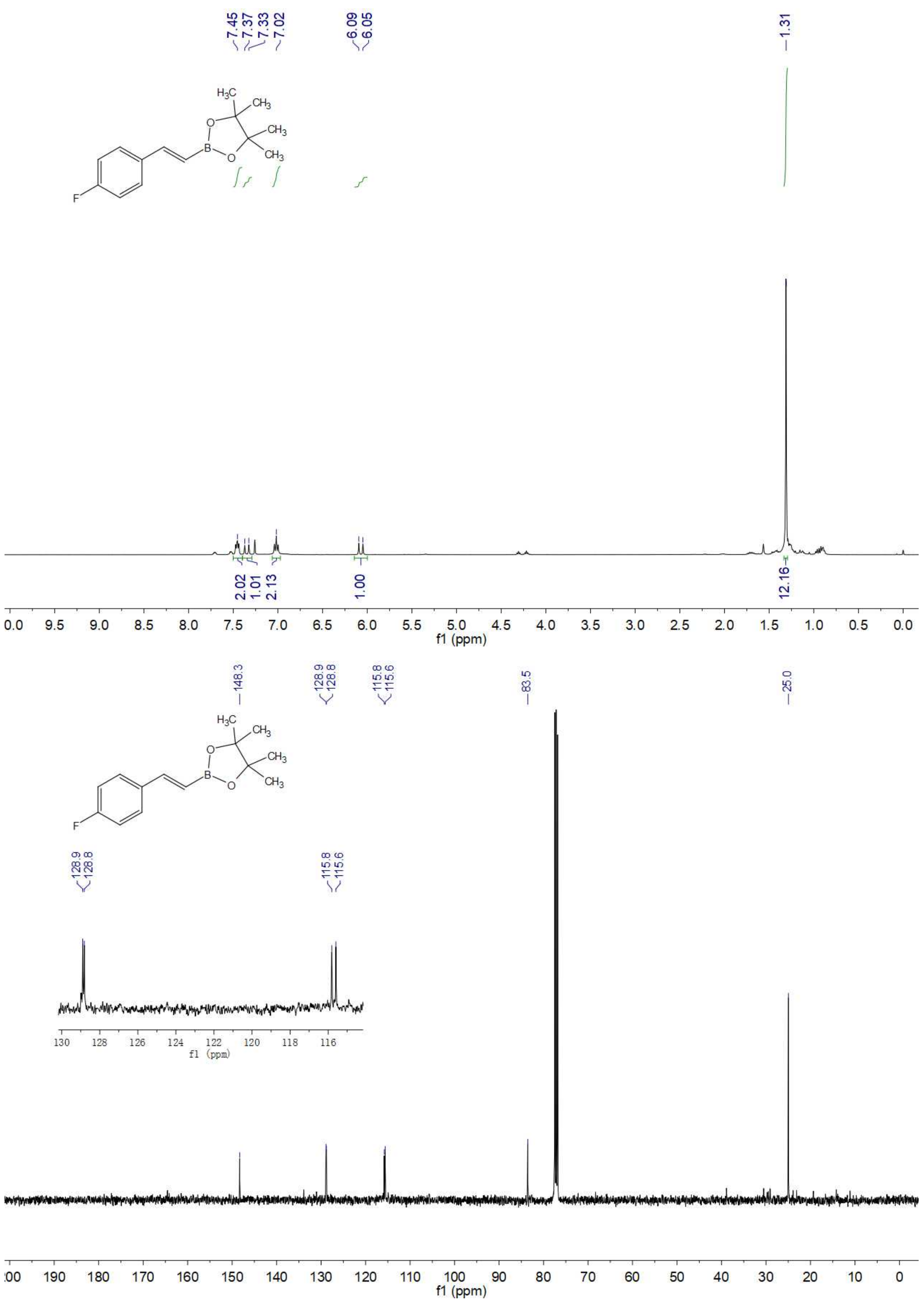

Supplementary Figure 15. **3d:** ^{1}H NMR (400 MHz, $CDCl_3$) (up) and ^{13}C NMR (101 MHz, $CDCl_3$) (down)

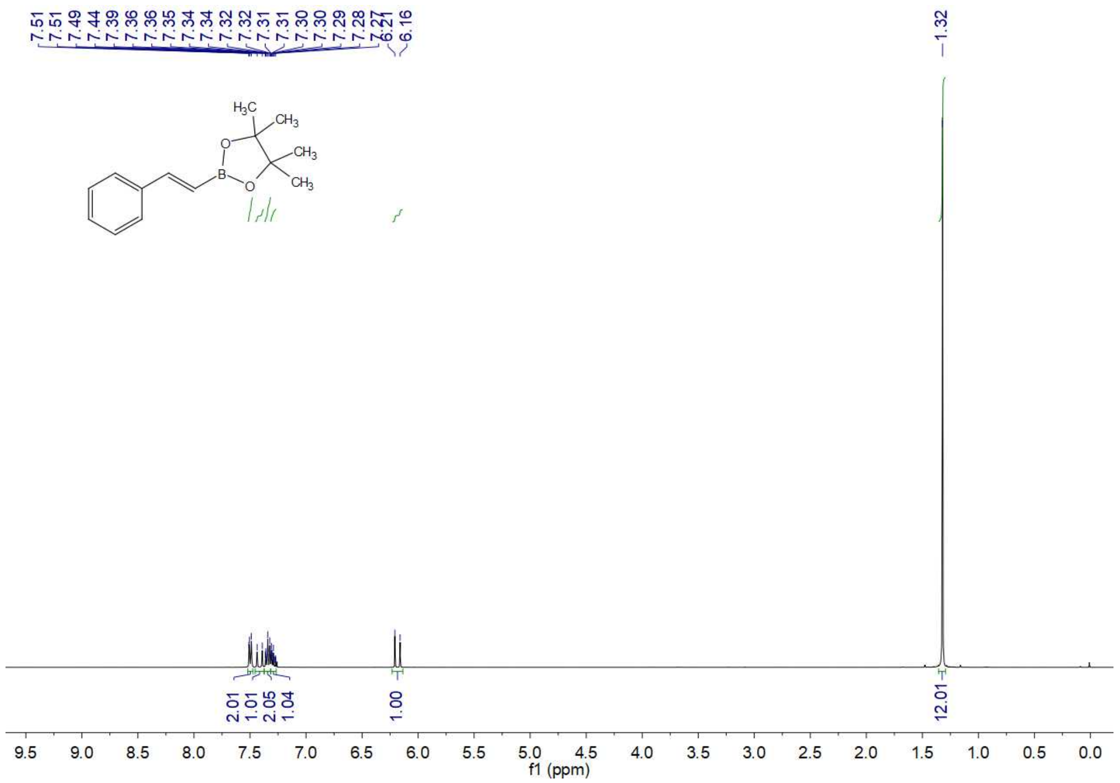

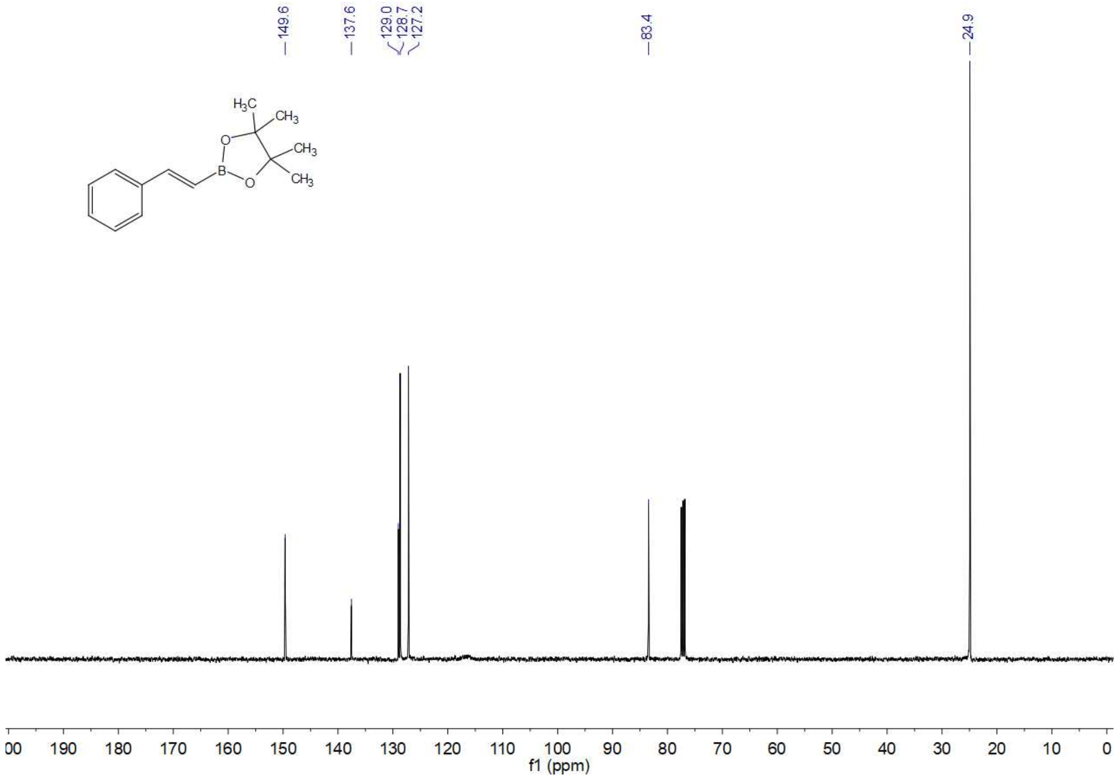

Supplementary Figure 16. **3e:** ^{1}H NMR (400 MHz, $CDCl_3$) (up) and ^{13}C NMR (101 MHz, $CDCl_3$) (down)

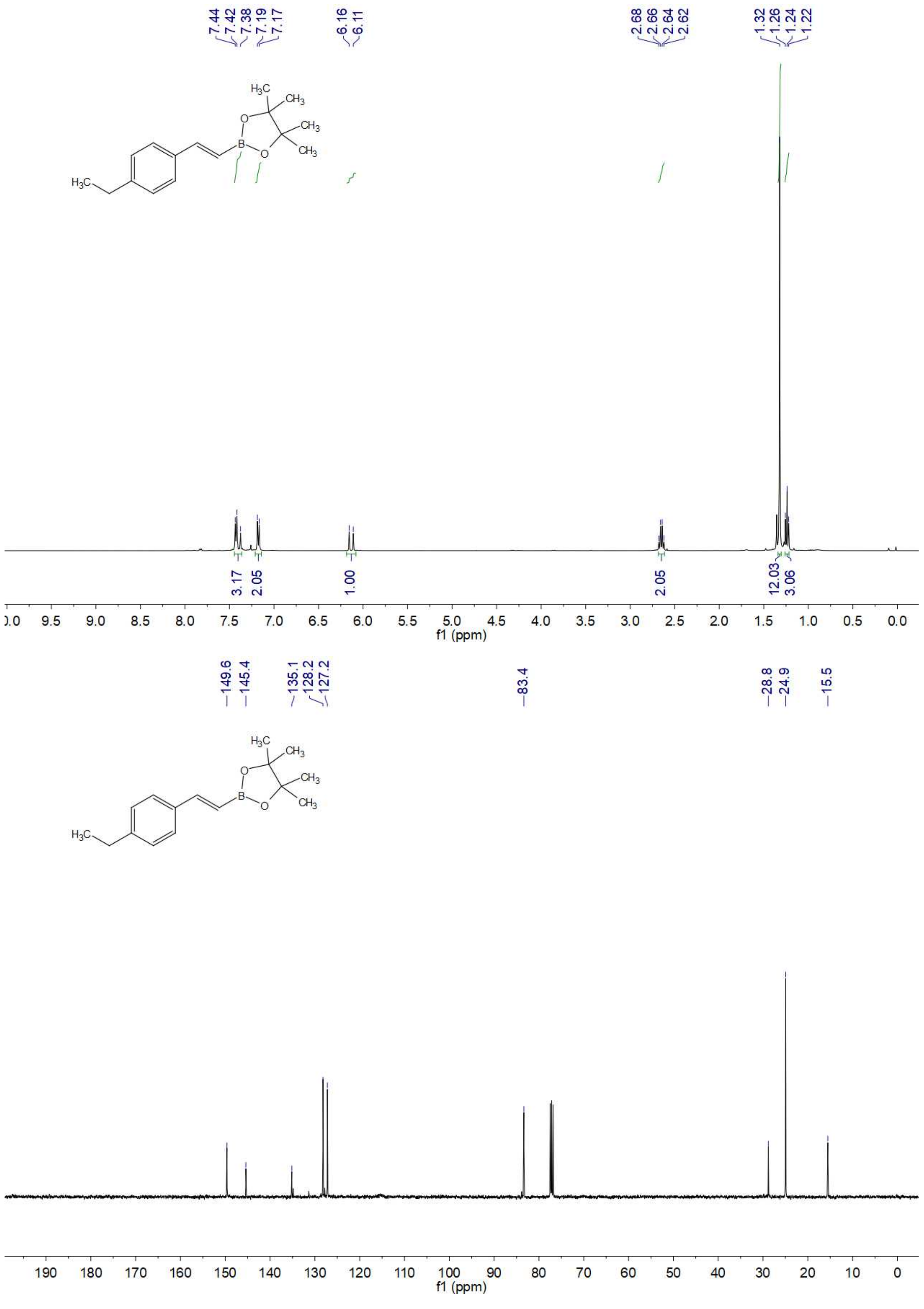

Supplementary Figure 17. **3f:** ^{1}H NMR (400 MHz, $CDCl_3$) (up) and ^{13}C NMR (101 MHz, $CDCl_3$) (down)

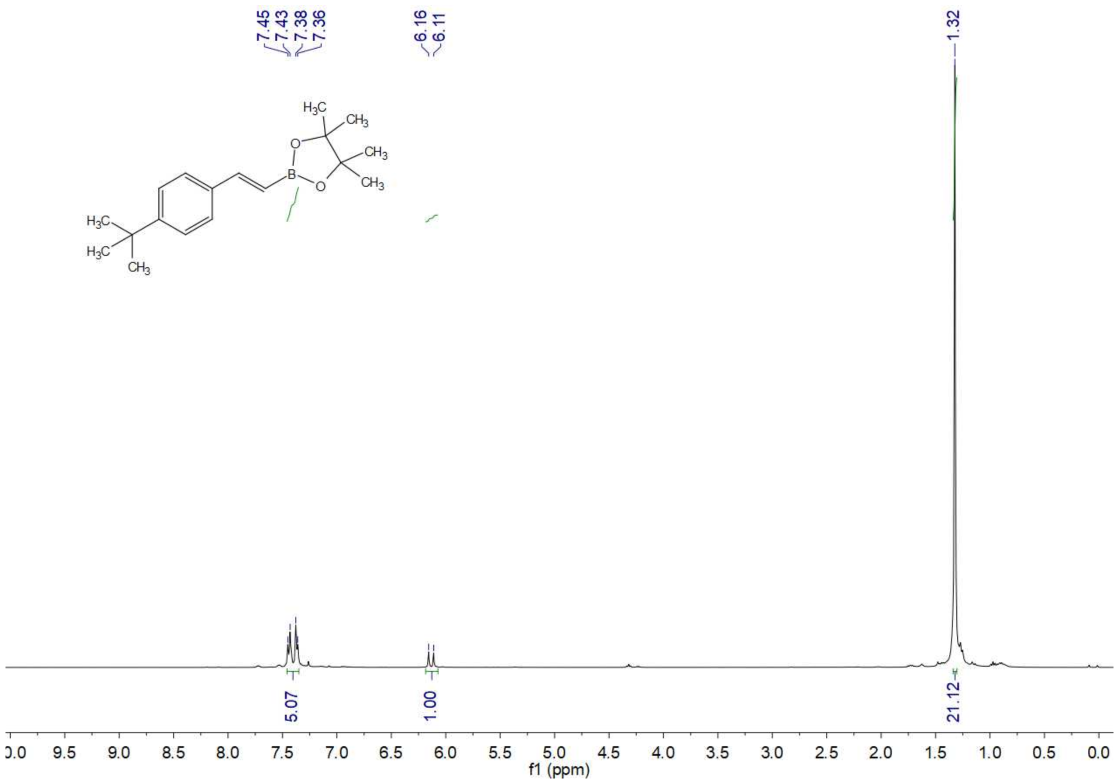

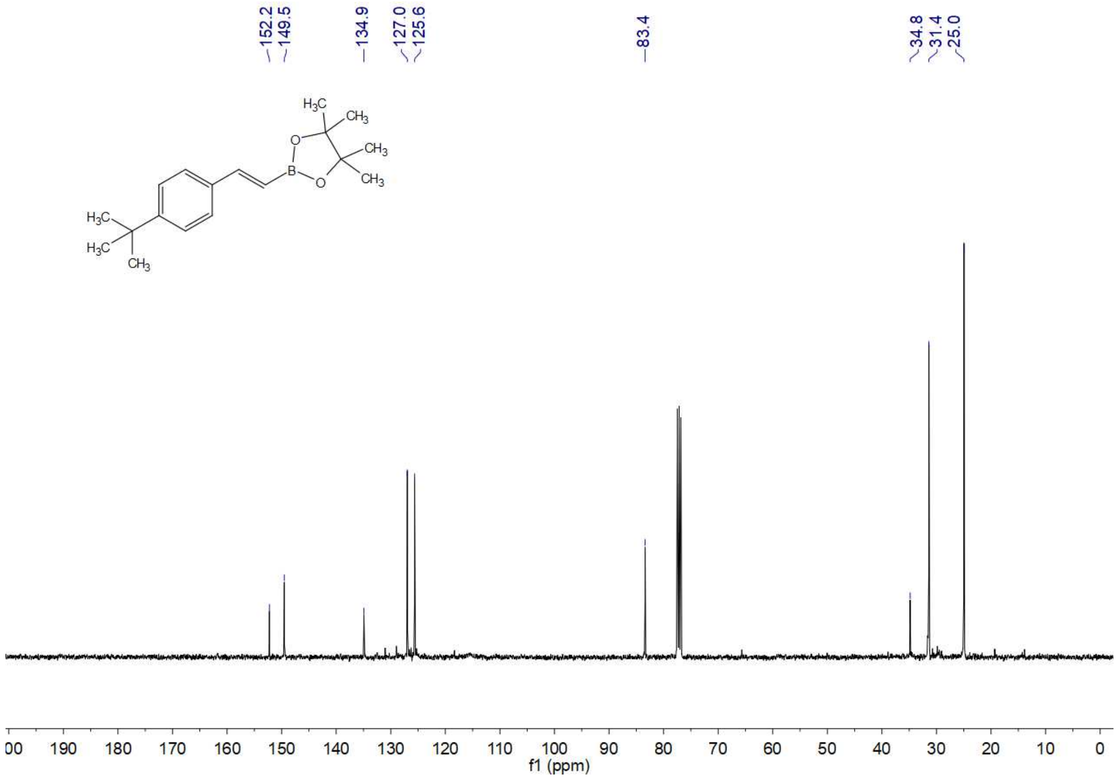

Supplementary Figure 18. **3g:** ^{1}H NMR (400 MHz, $CDCl_3$) (up) and ^{13}C NMR (101 MHz, $CDCl_3$) (down)

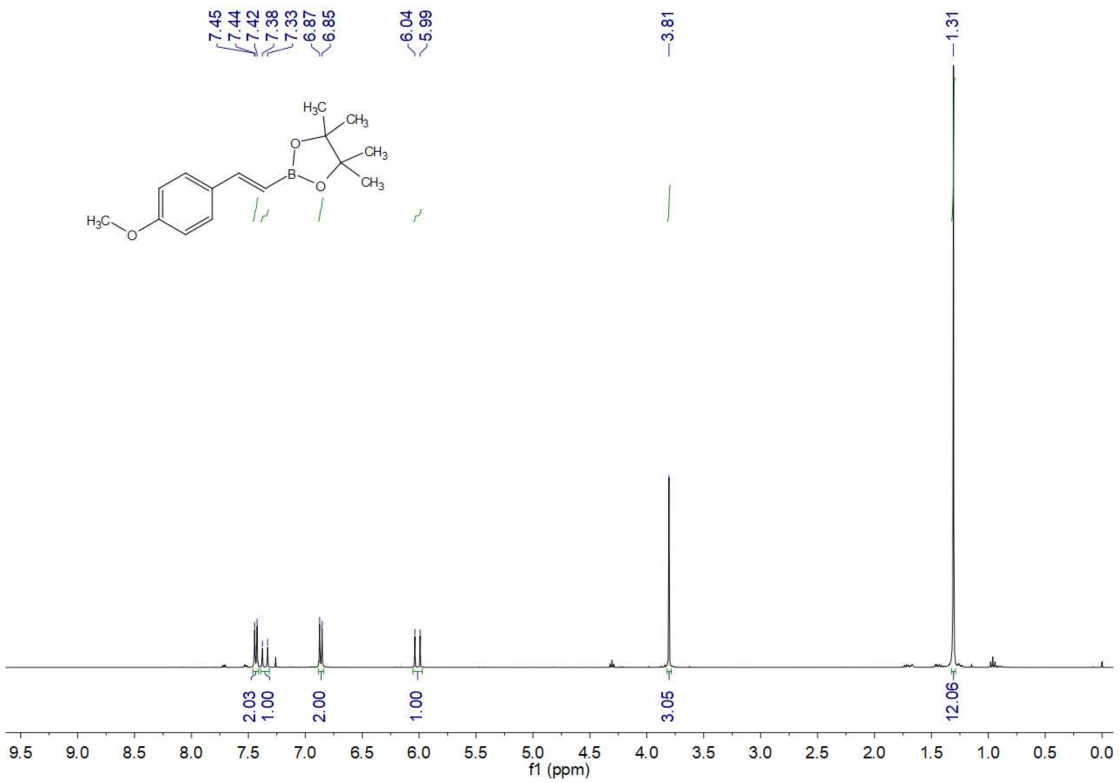

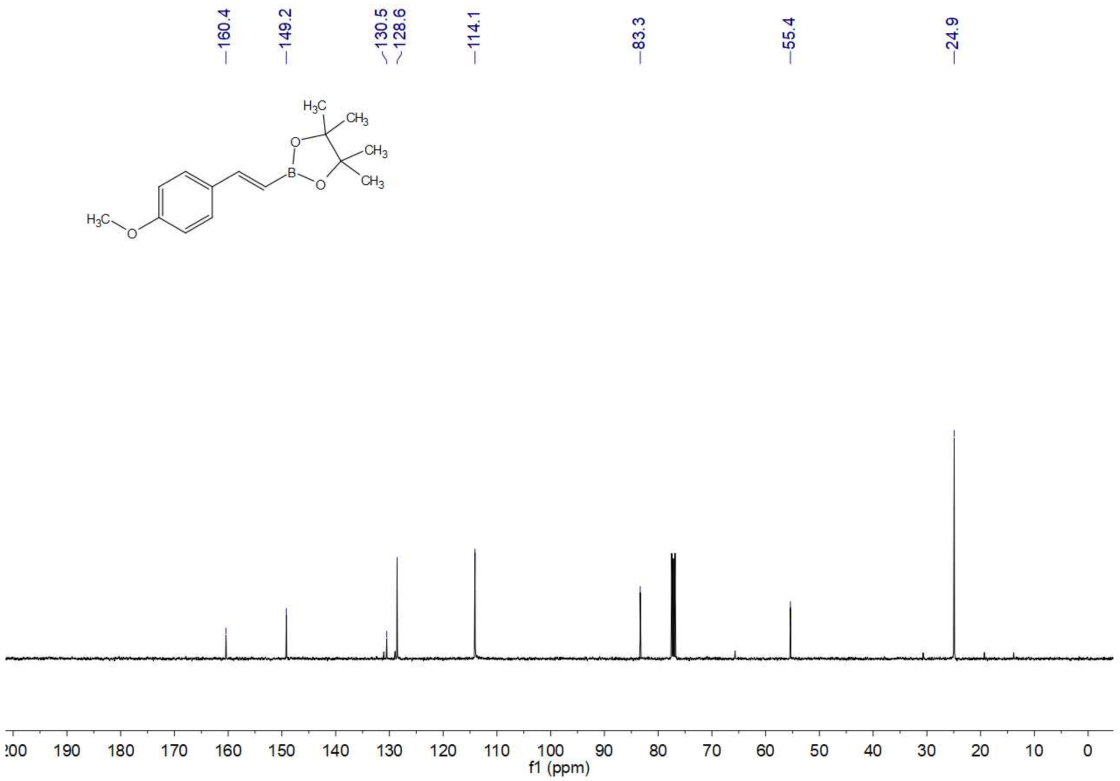

Supplementary Figure 19. **3h:** ^{1}H NMR (400 MHz, $CDCl_3$) (up) and ^{13}C NMR (101 MHz, $CDCl_3$) (down)

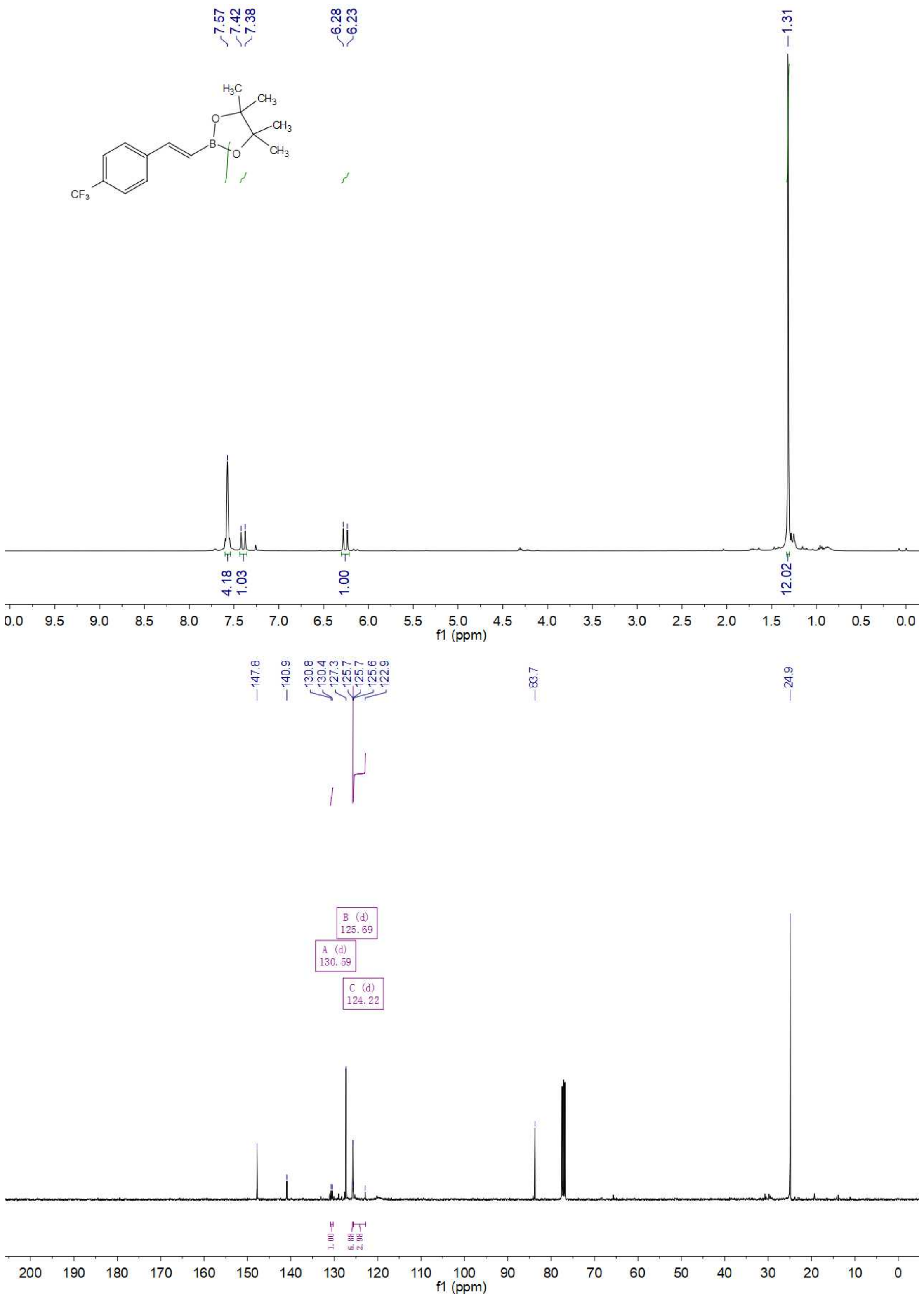

Supplementary Figure 20. **3i:** ^{1}H NMR (400 MHz, $CDCl_3$) (up) and ^{13}C NMR (101 MHz, $CDCl_3$) (down)

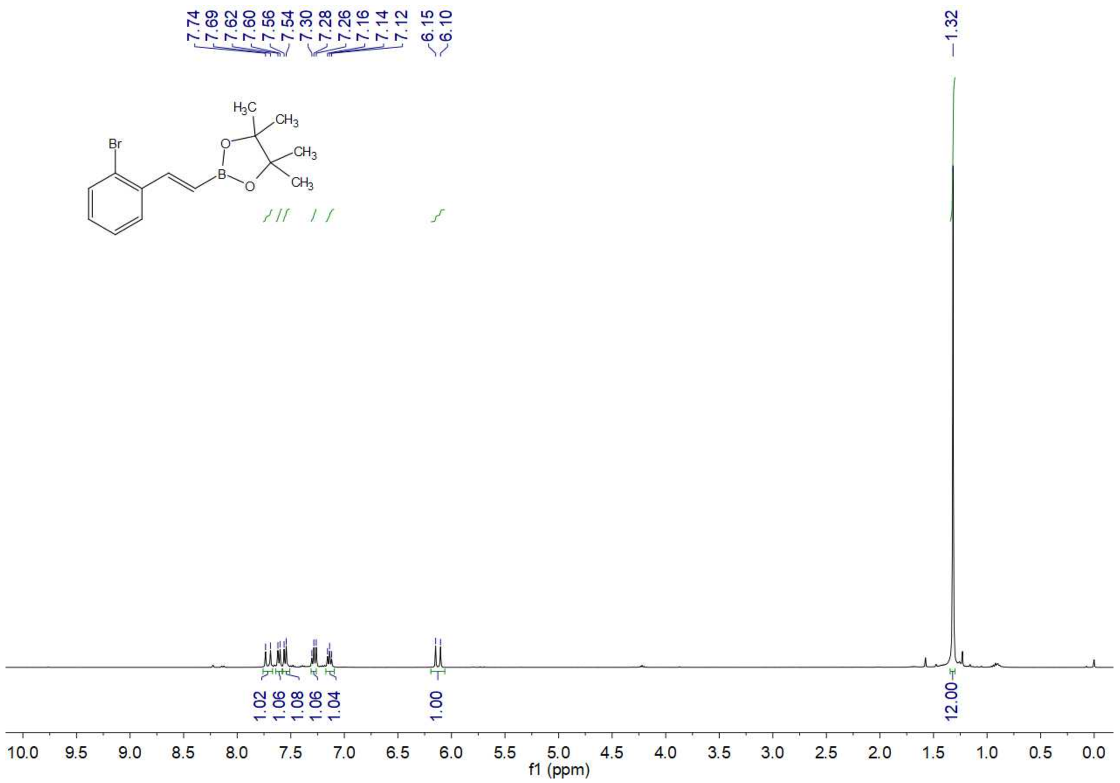

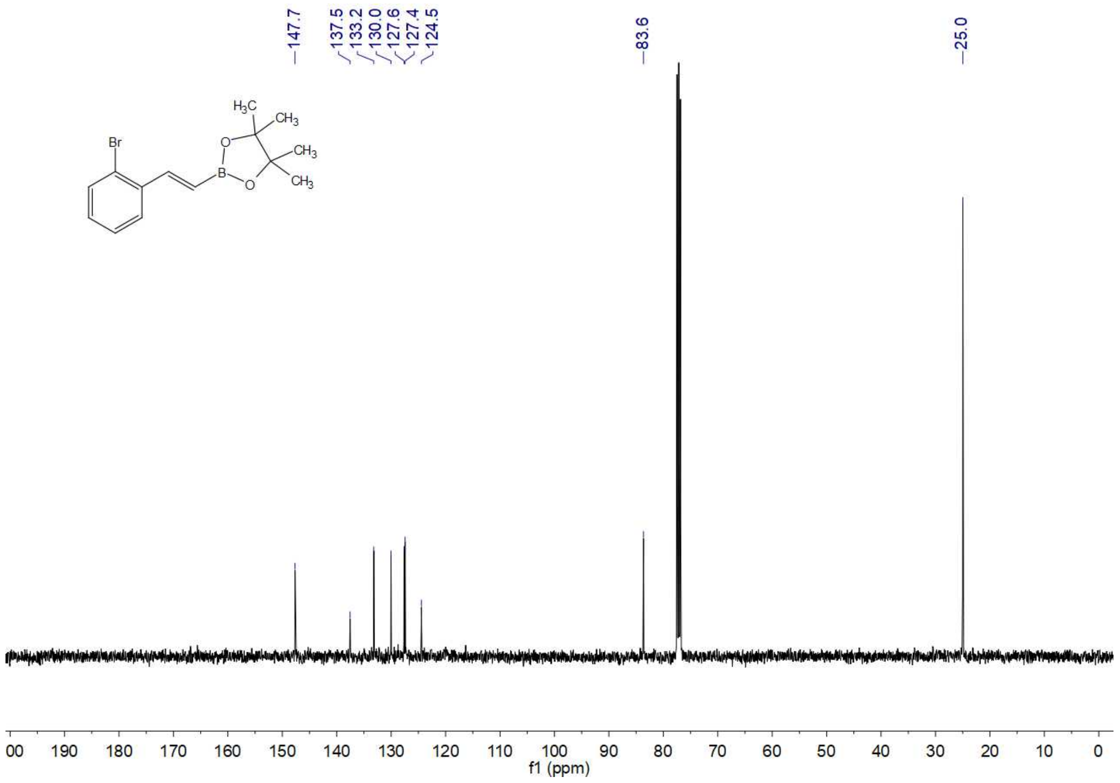

Supplementary Figure 21. **3j:** ^{1}H NMR (400 MHz, $CDCl_3$) (up) and ^{13}C NMR (101 MHz, $CDCl_3$) (down)

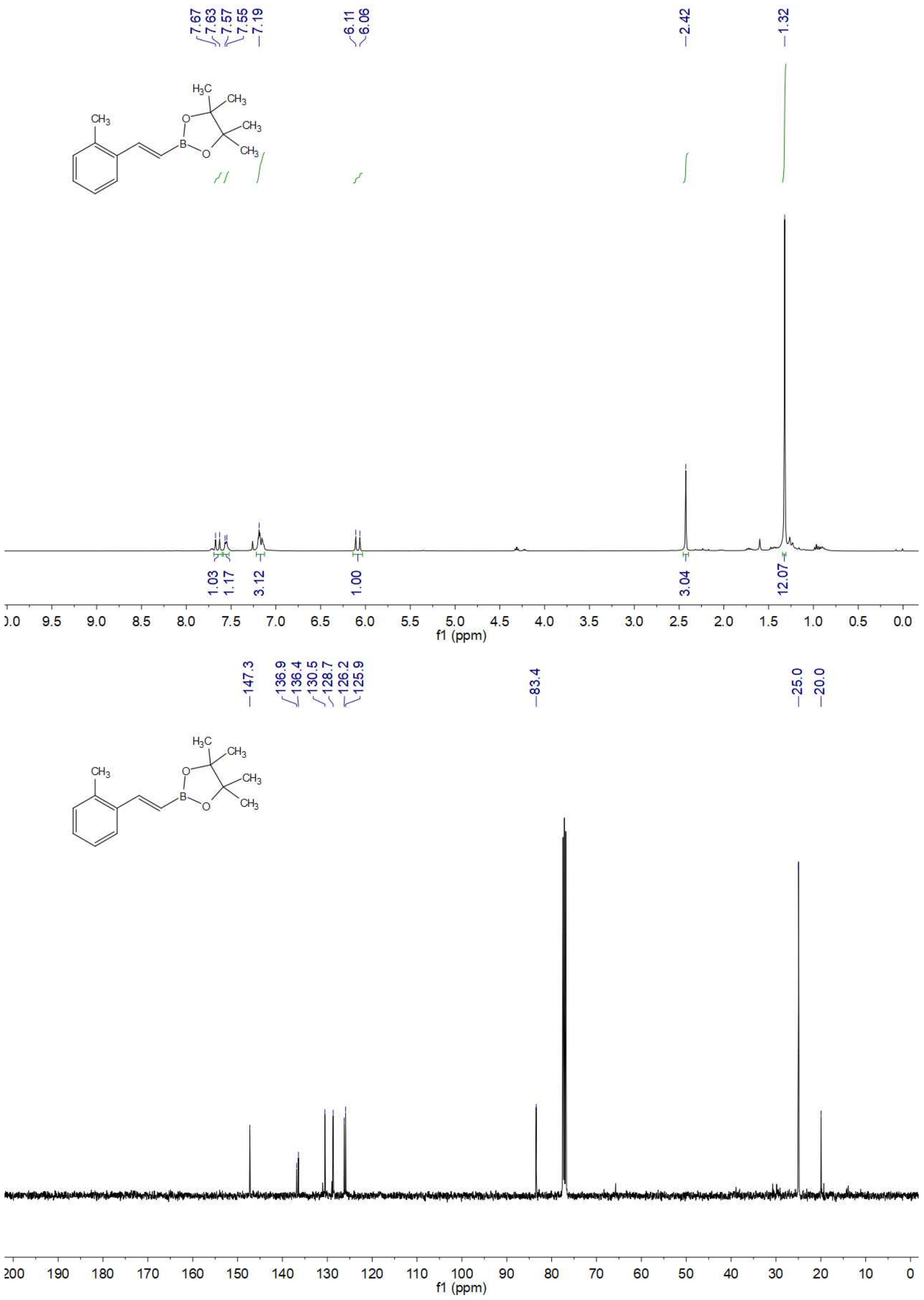

Supplementary Figure 22. **3k:** ^{1}H NMR (400 MHz, $CDCl_3$) (up) and ^{13}C NMR (101 MHz, $CDCl_3$) (down)

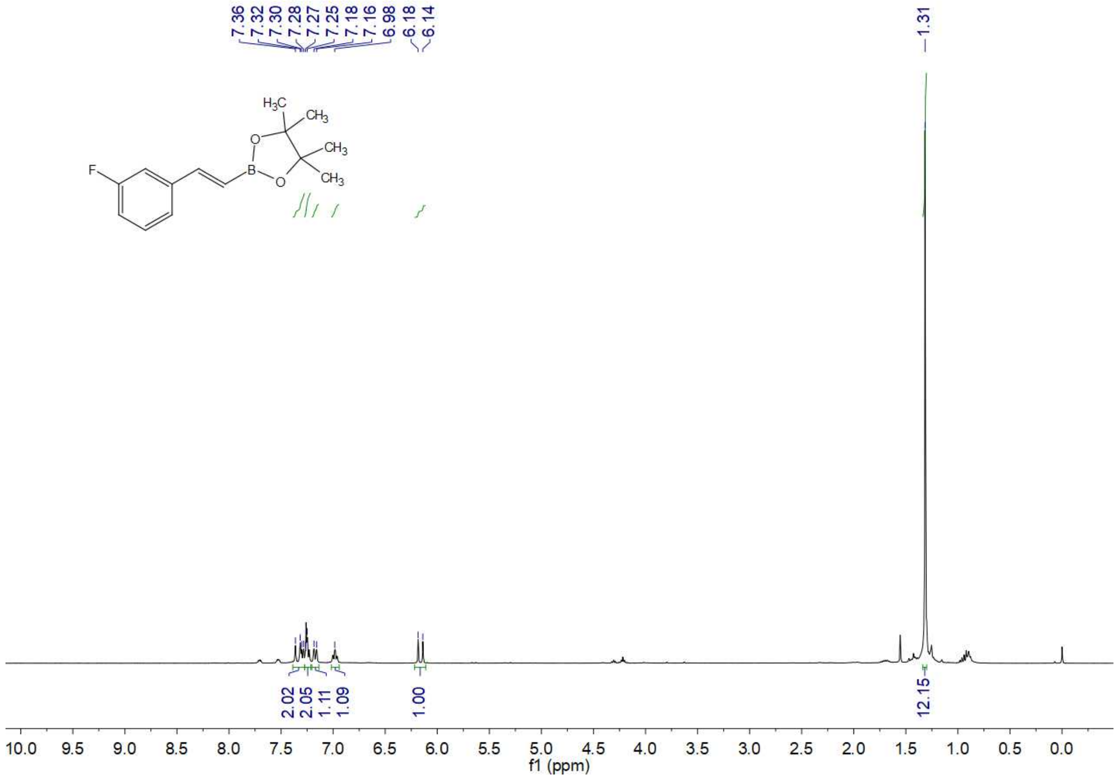

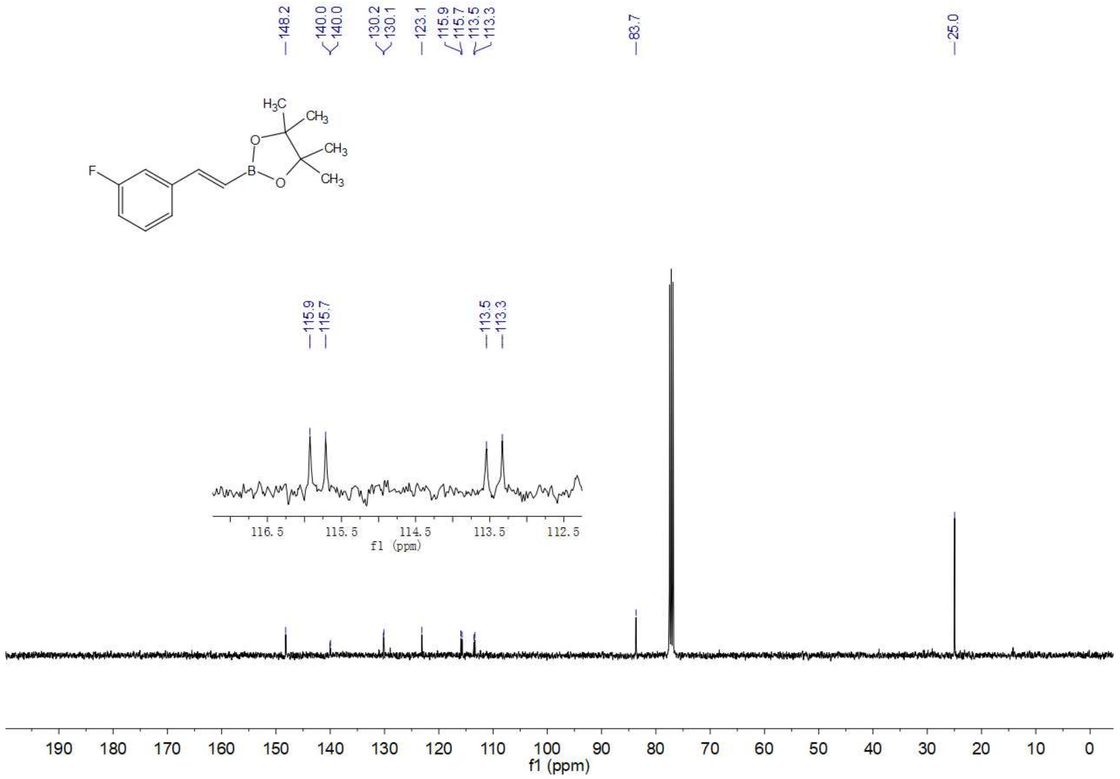

Supplementary Figure 23. **3l:** ^{1}H NMR (400 MHz, $CDCl_3$) (up) and ^{13}C NMR (101 MHz, $CDCl_3$) (down)

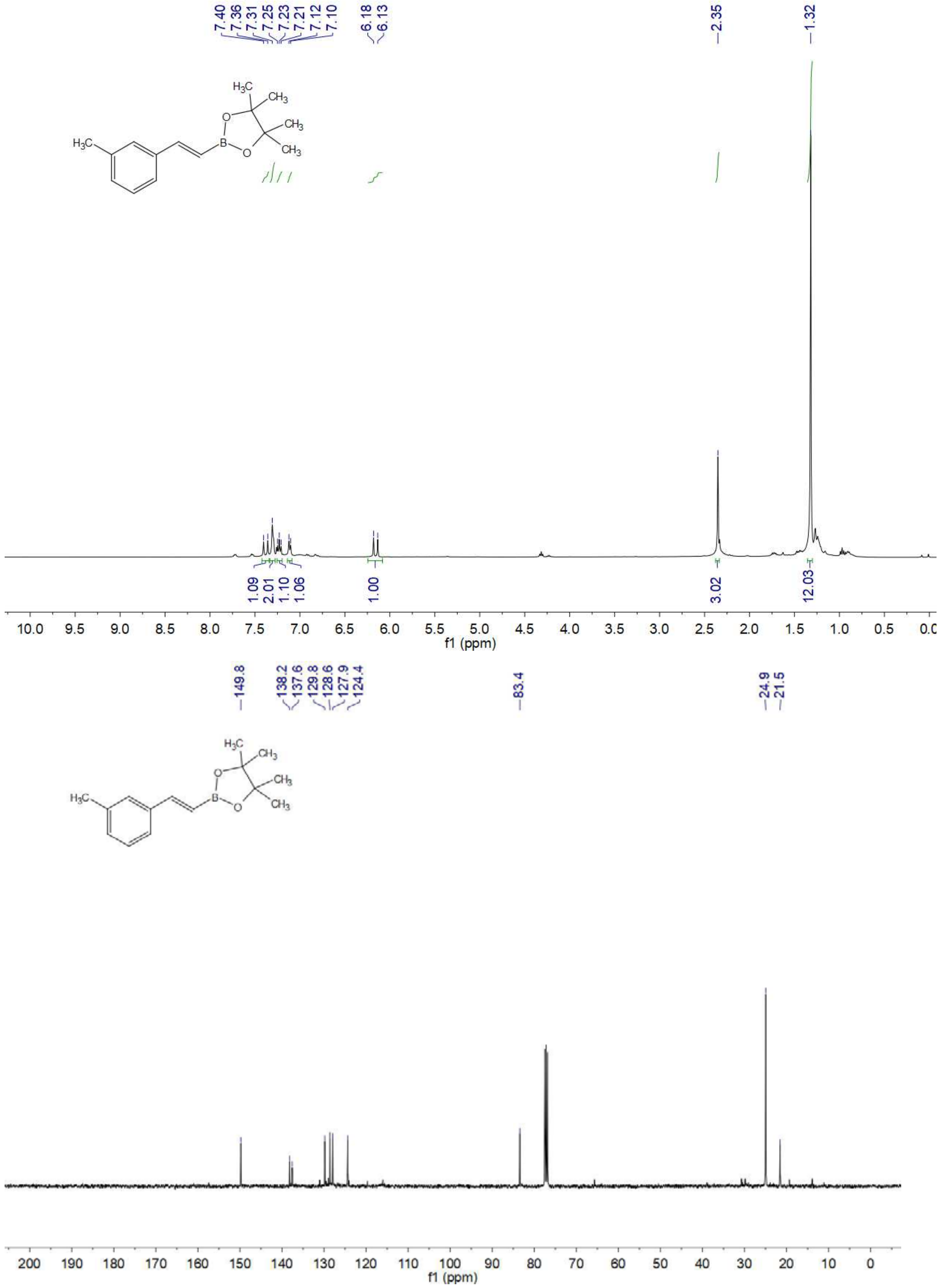

Supplementary Figure 24. **3m:** ^{1}H NMR (400 MHz, $CDCl_3$) (up) and ^{13}C NMR (101 MHz, $CDCl_3$) (down)

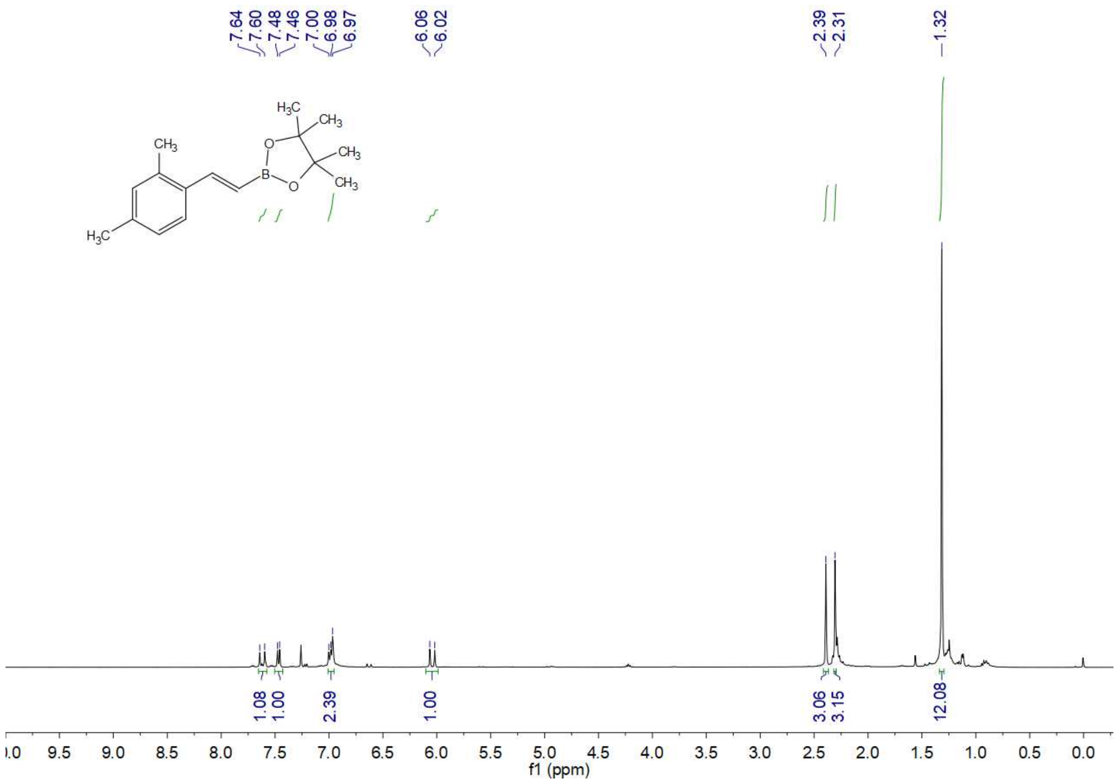

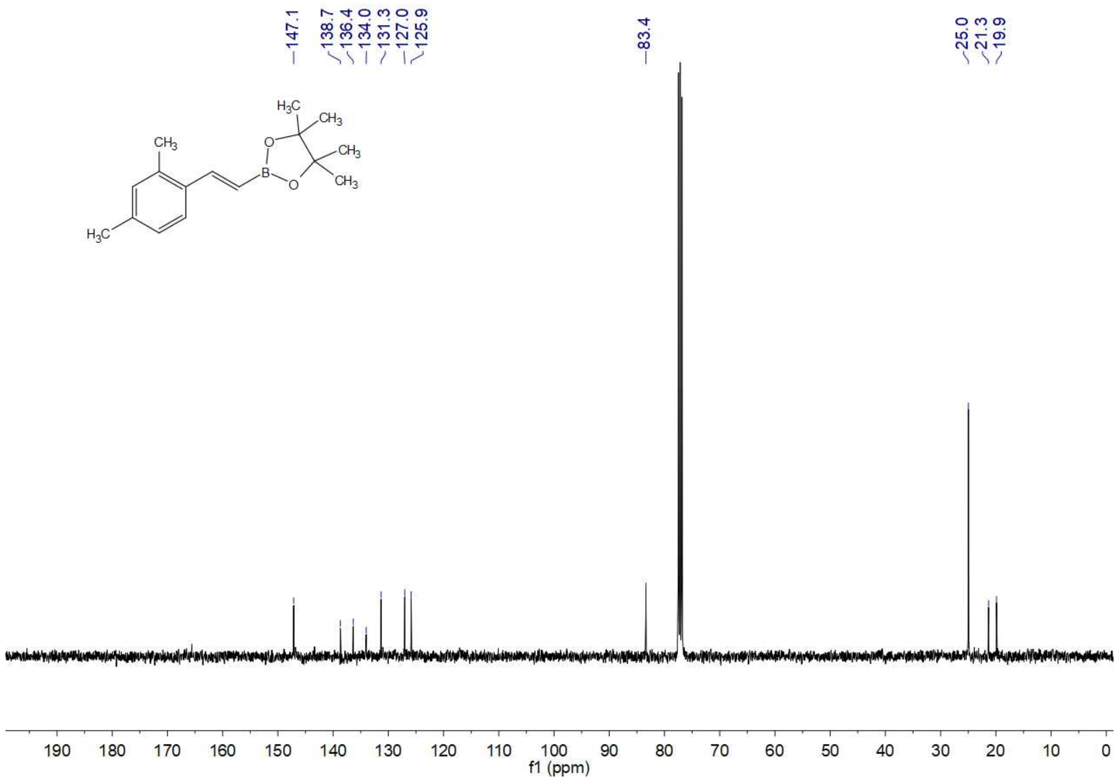

Supplementary Figure 25. **3n:** ^{1}H NMR (400 MHz, $CDCl_3$) (up) and ^{13}C NMR (101 MHz, $CDCl_3$) (down)

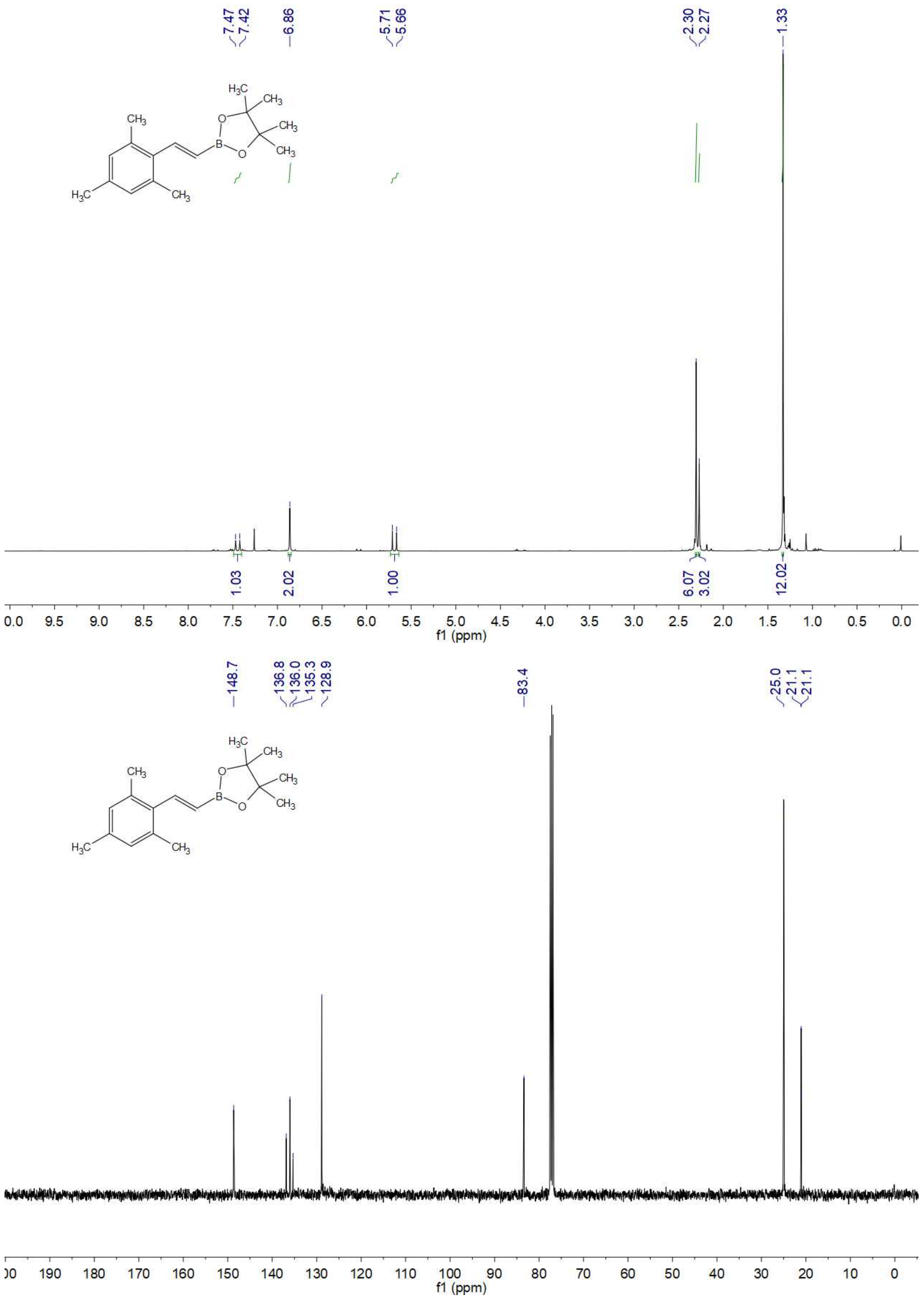

Supplementary Figure 26. **3o:** ^{1}H NMR (400 MHz, $CDCl_3$) (up) and ^{13}C NMR (101 MHz, $CDCl_3$) (down)

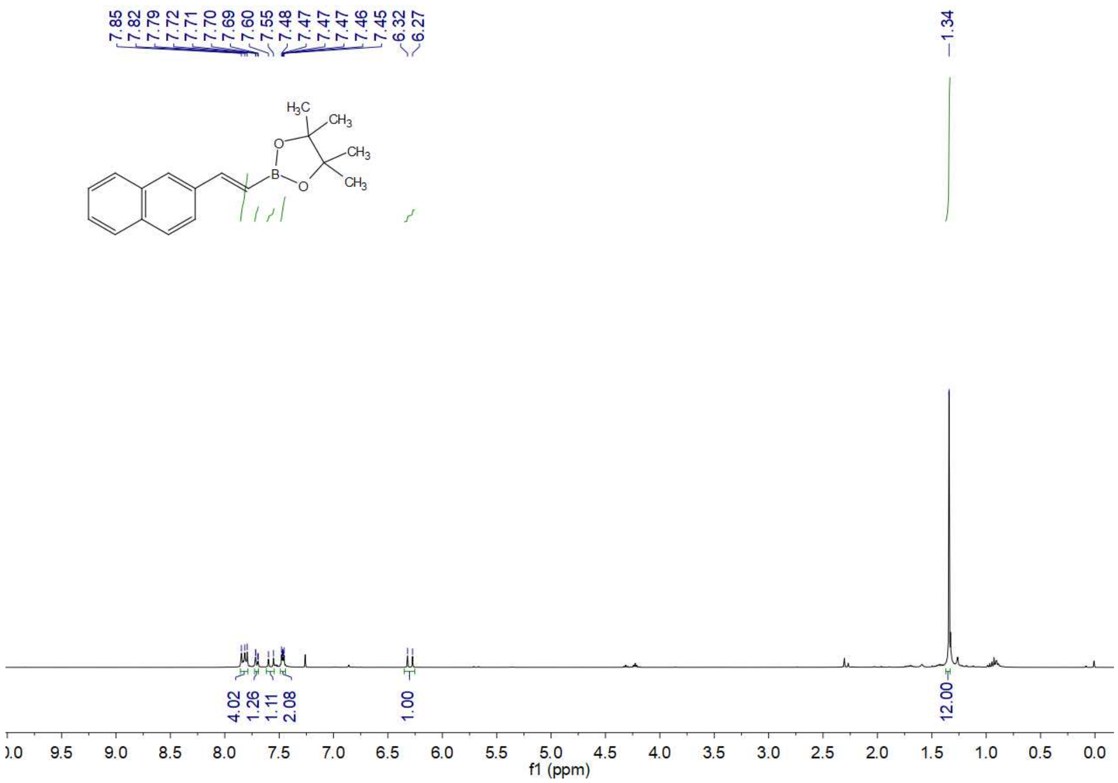

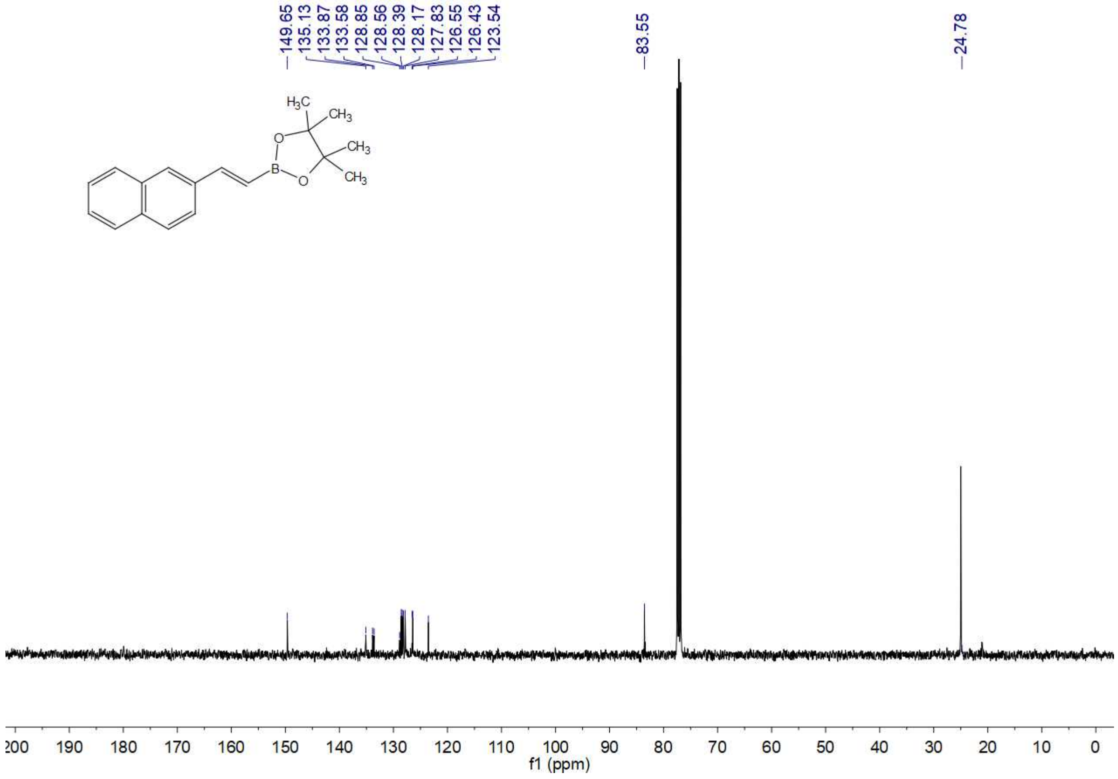

Supplementary Figure 27. **3p:** ^{1}H NMR (400 MHz, $CDCl_3$) (up) and ^{13}C NMR (101 MHz, $CDCl_3$) (down)

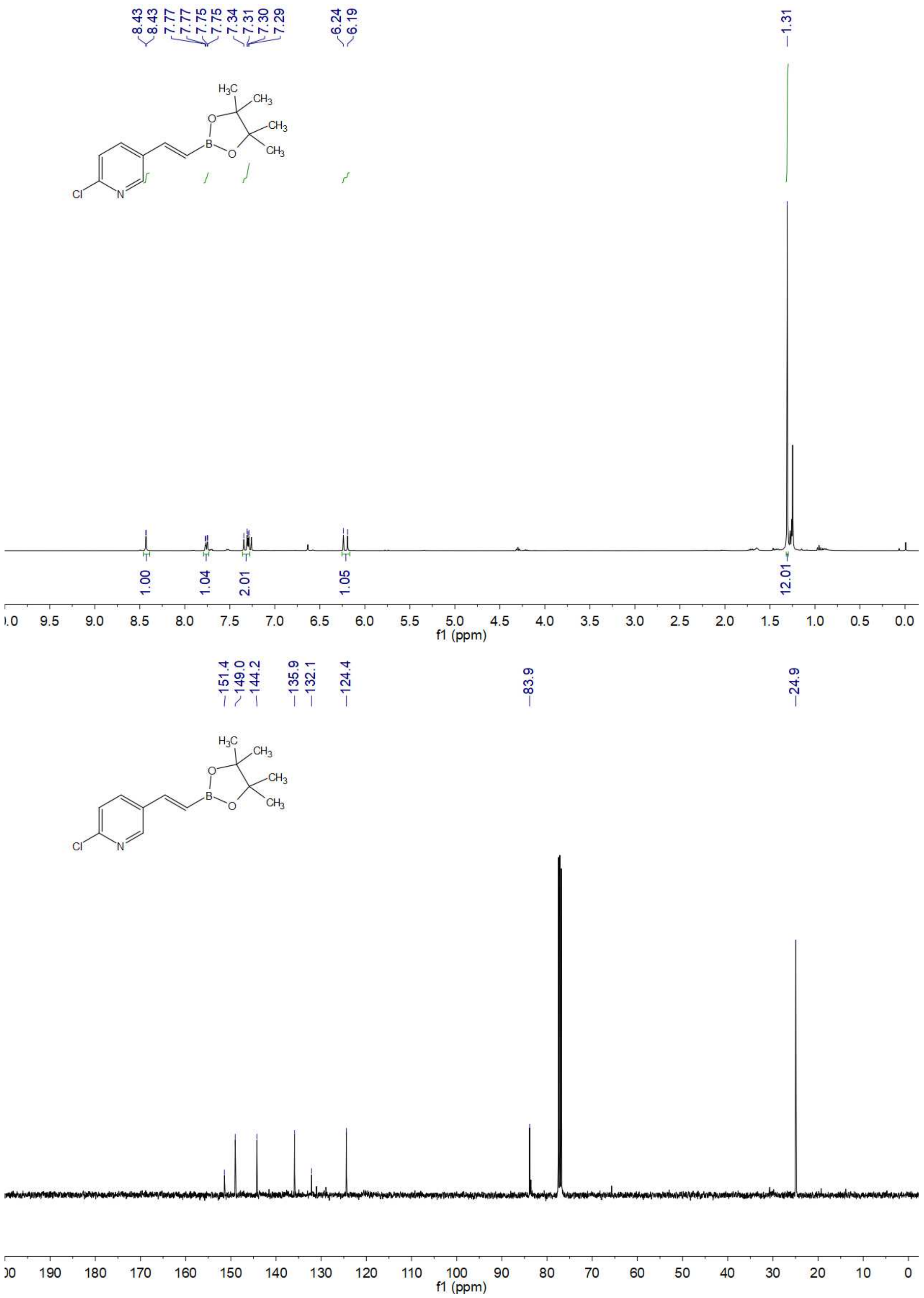

Supplementary Figure 28. **3q:** ^{1}H NMR (400 MHz, $CDCl_3$) (up) and ^{13}C NMR (101 MHz, $CDCl_3$) (down)

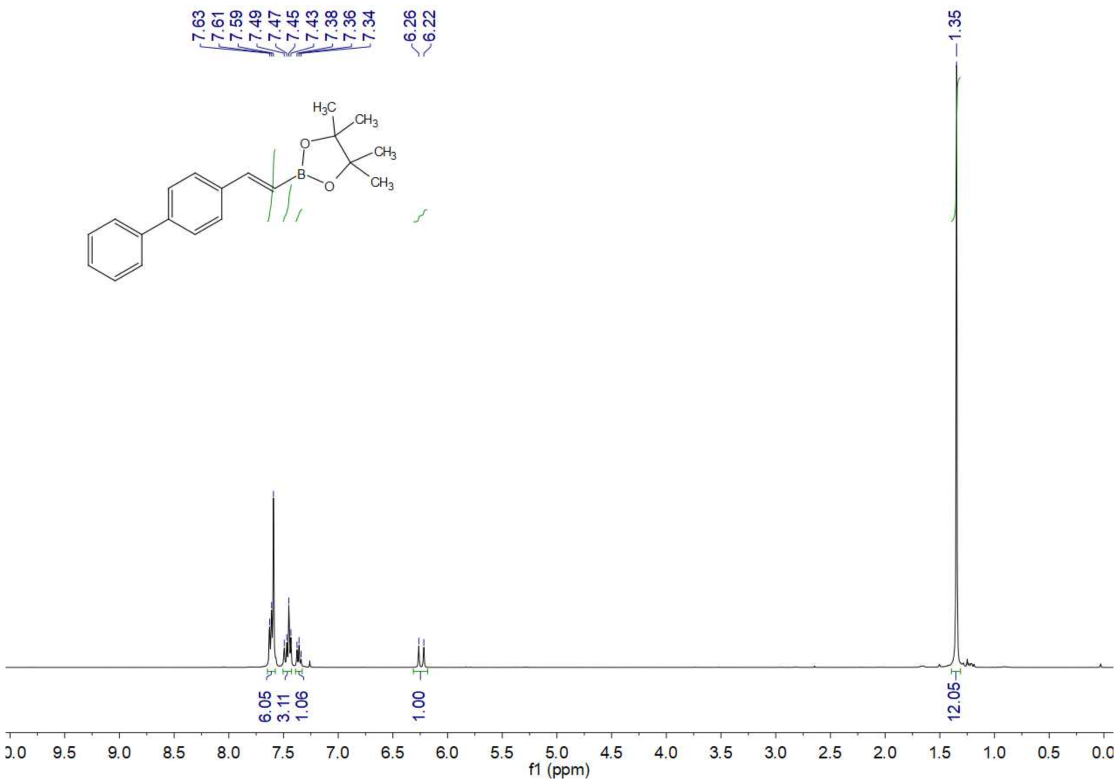

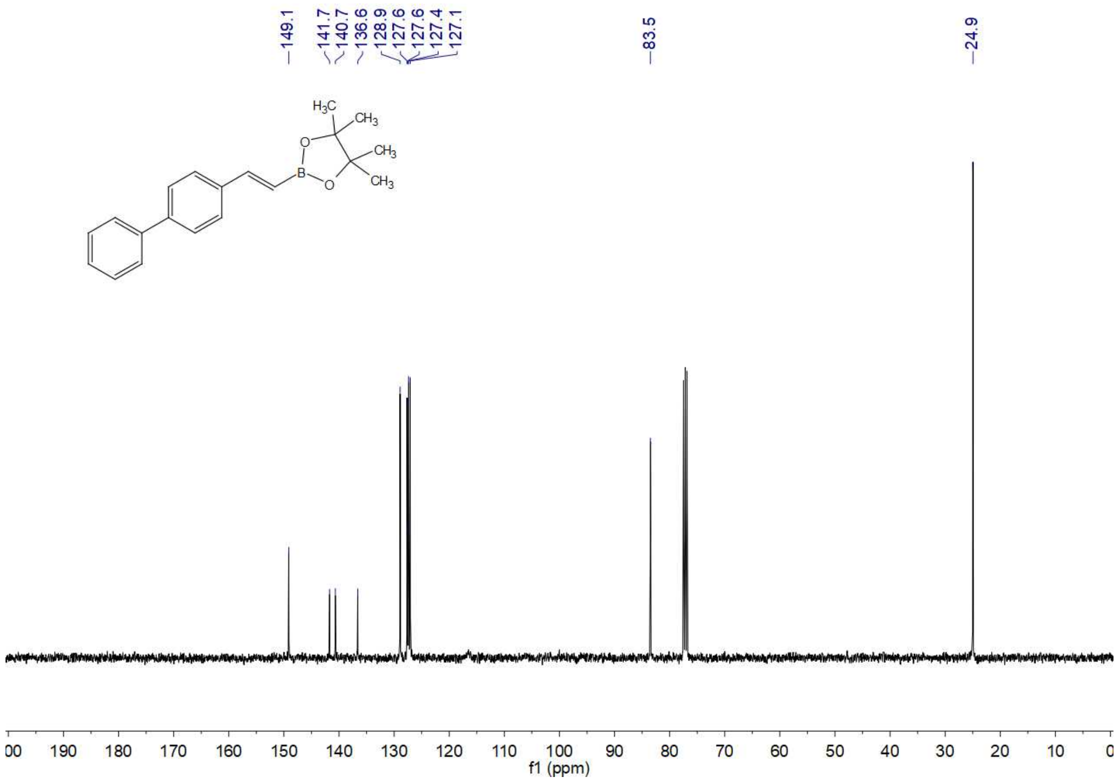

Supplementary Figure 29. **3r:** ^{1}H NMR (400 MHz, $CDCl_3$) (up) and ^{13}C NMR (101 MHz, $CDCl_3$) (down)

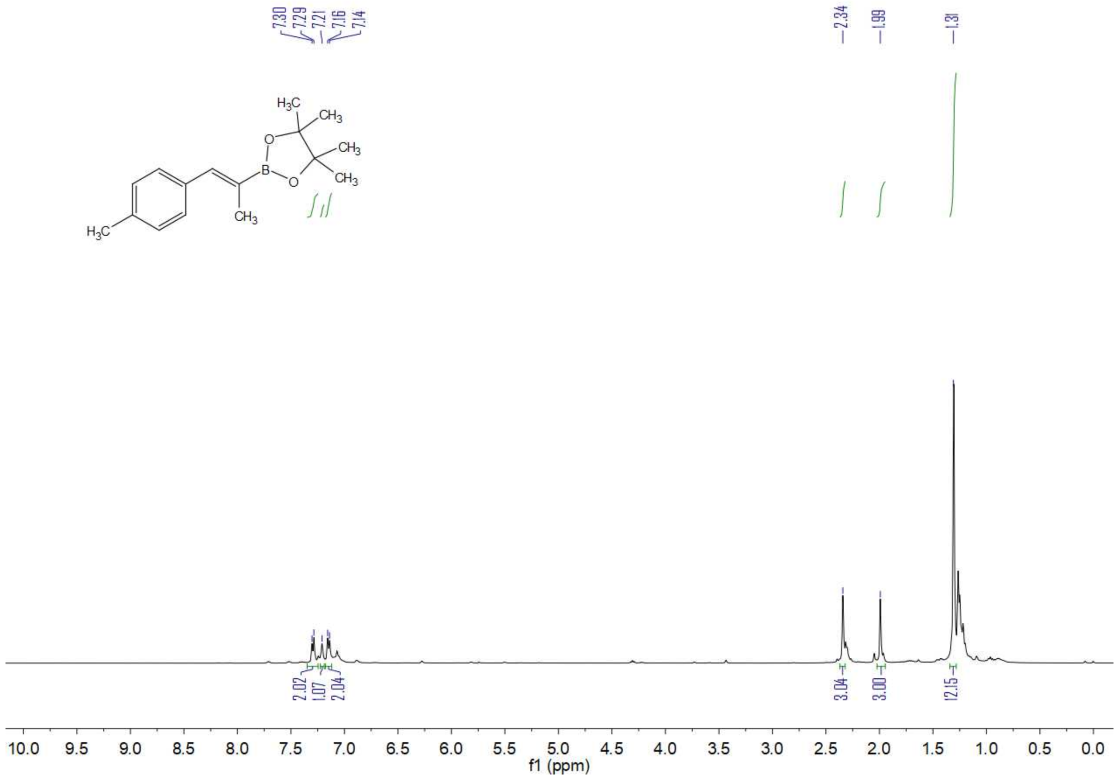

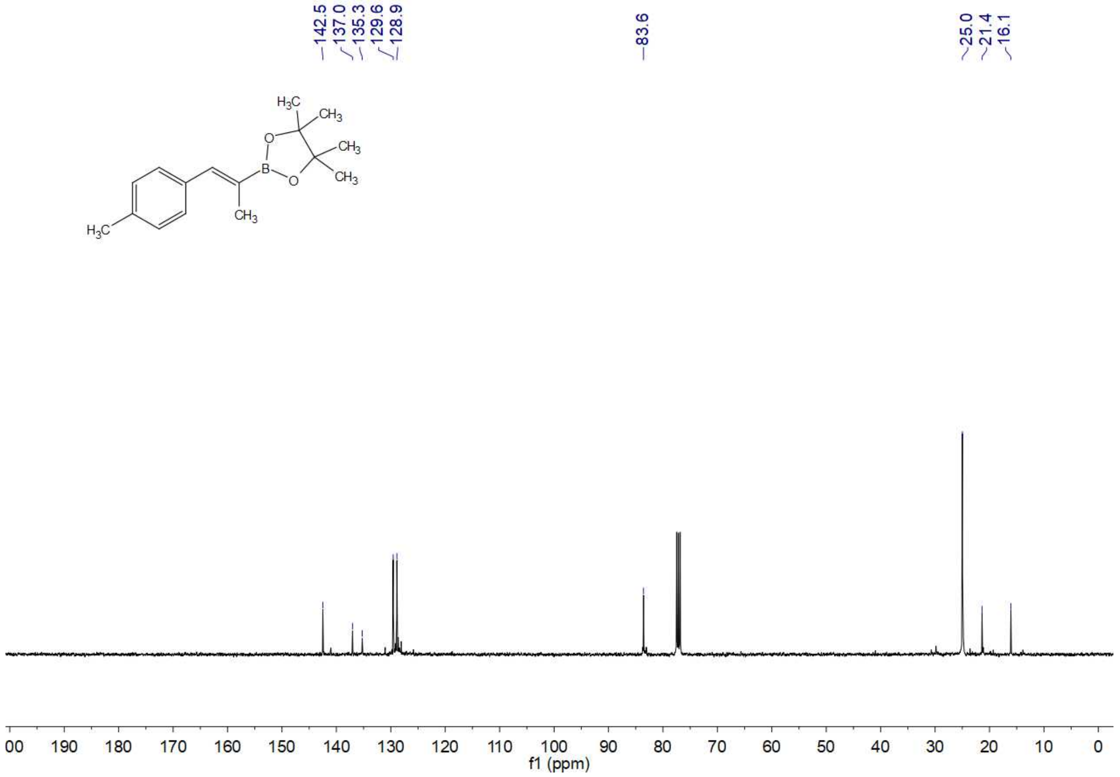

Supplementary Figure 30. **3t:** ^{1}H NMR (400 MHz, $CDCl_3$) (up) and ^{13}C NMR (101 MHz, $CDCl_3$) (down)

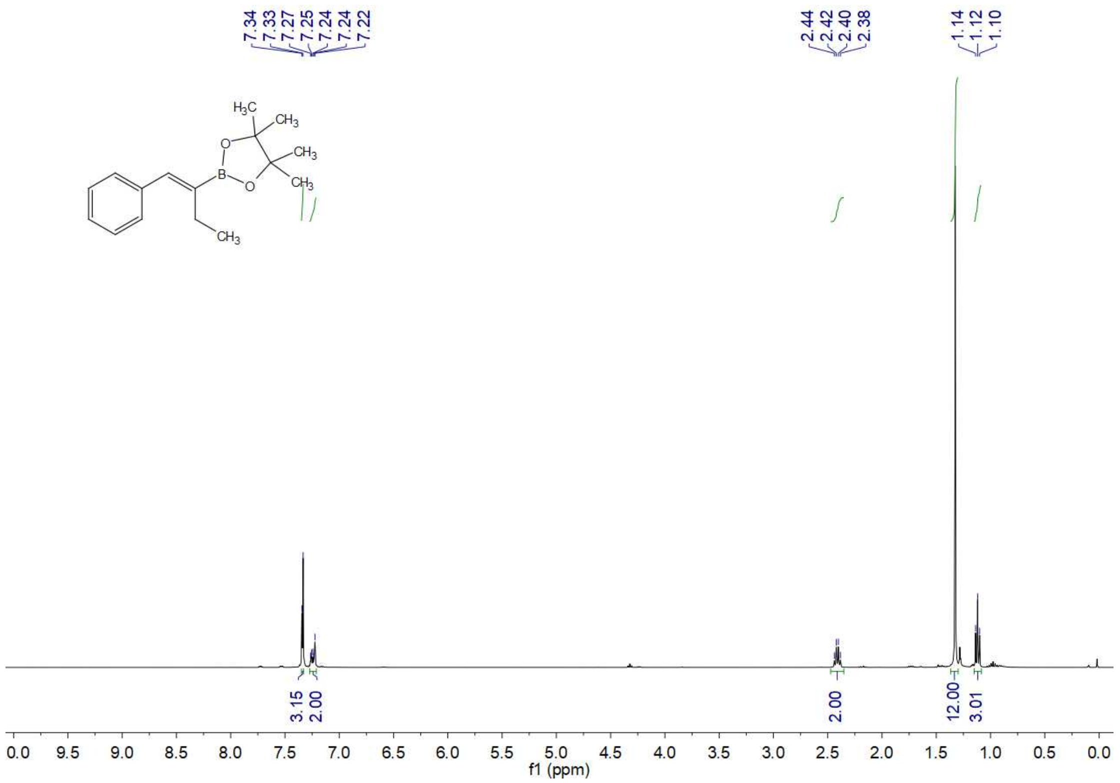

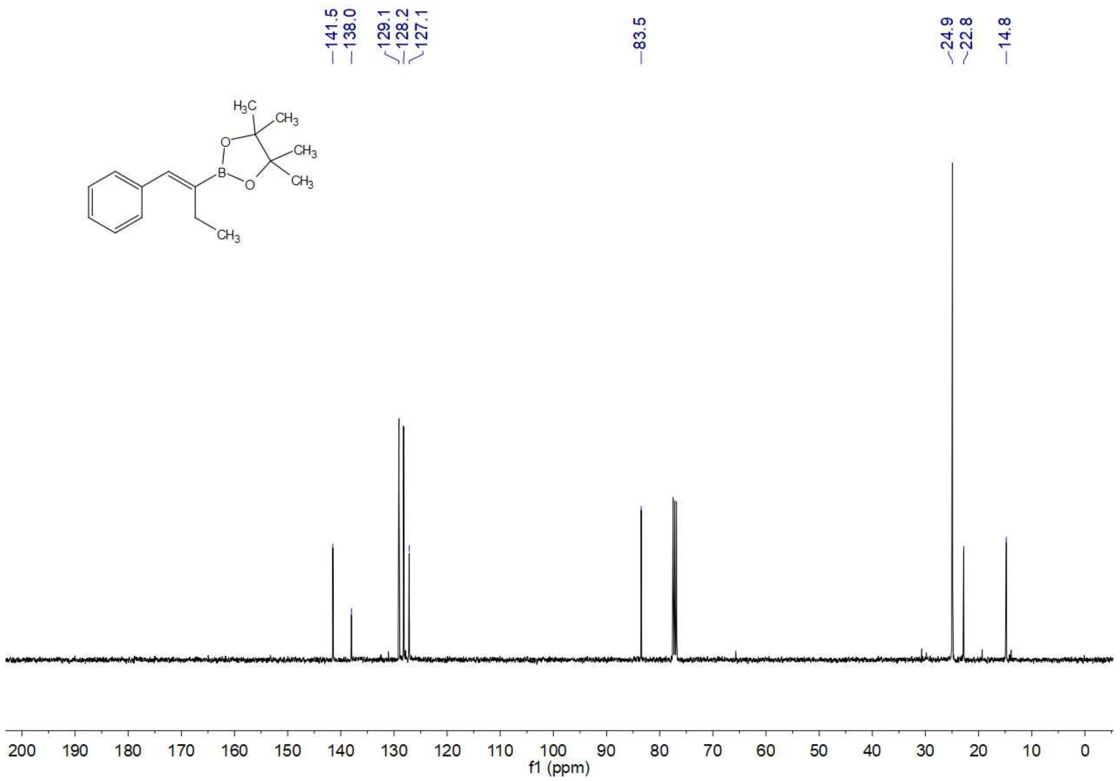

Supplementary Figure 31. **3u:** ^{1}H NMR (400 MHz, $CDCl_3$) (up) and ^{13}C NMR (101 MHz, $CDCl_3$) (down)

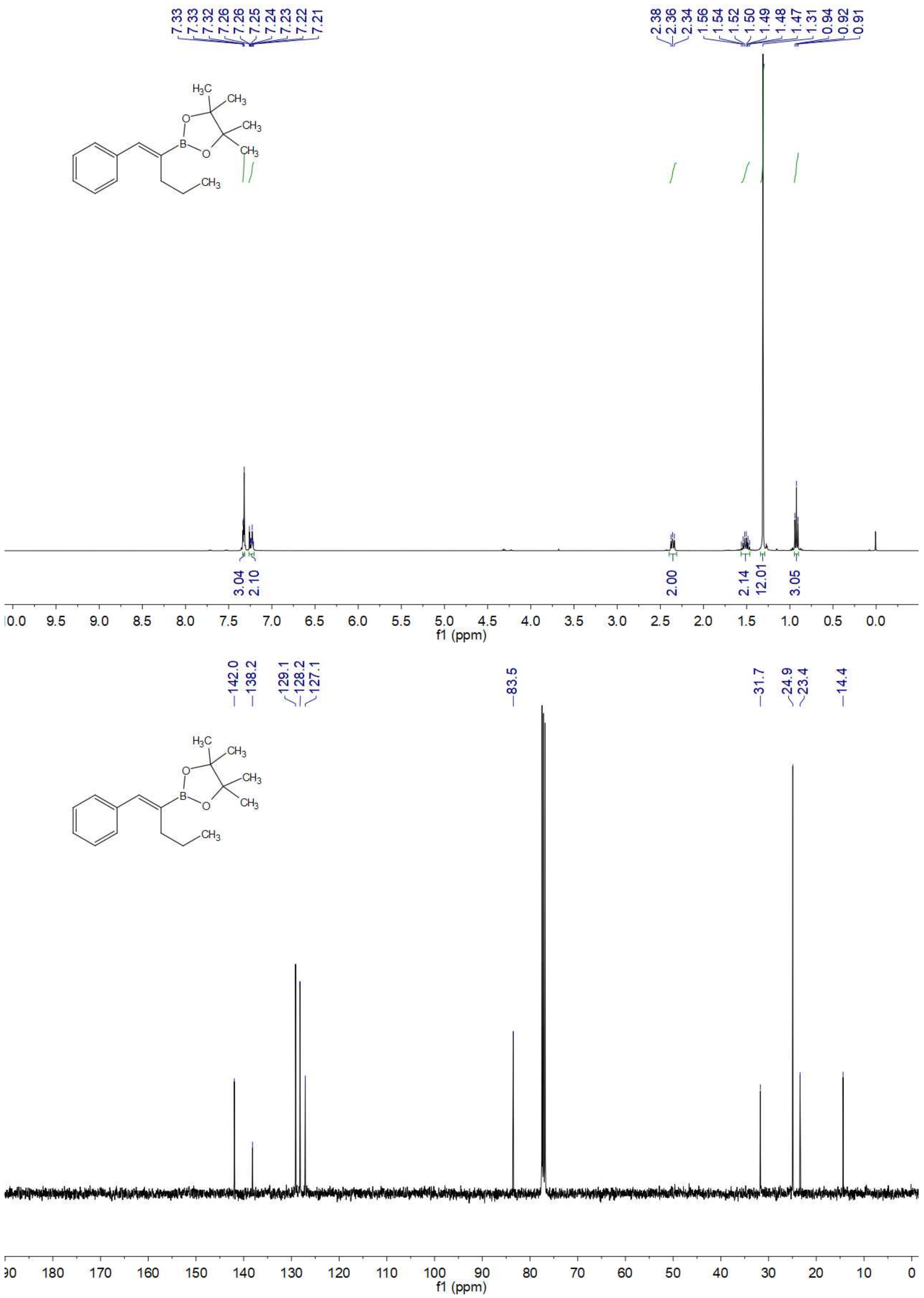

Supplementary Figure 32. **3v:** ^{1}H NMR (400 MHz, $CDCl_3$) (up) and ^{13}C NMR (101 MHz, $CDCl_3$) (down)

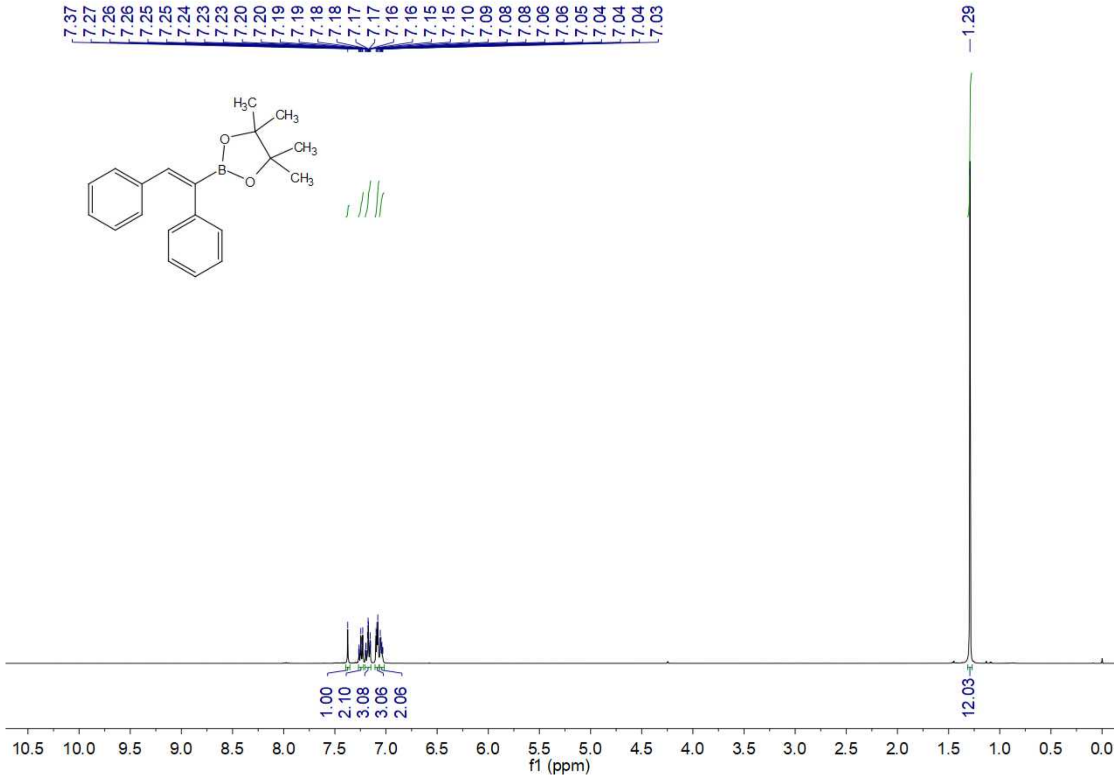

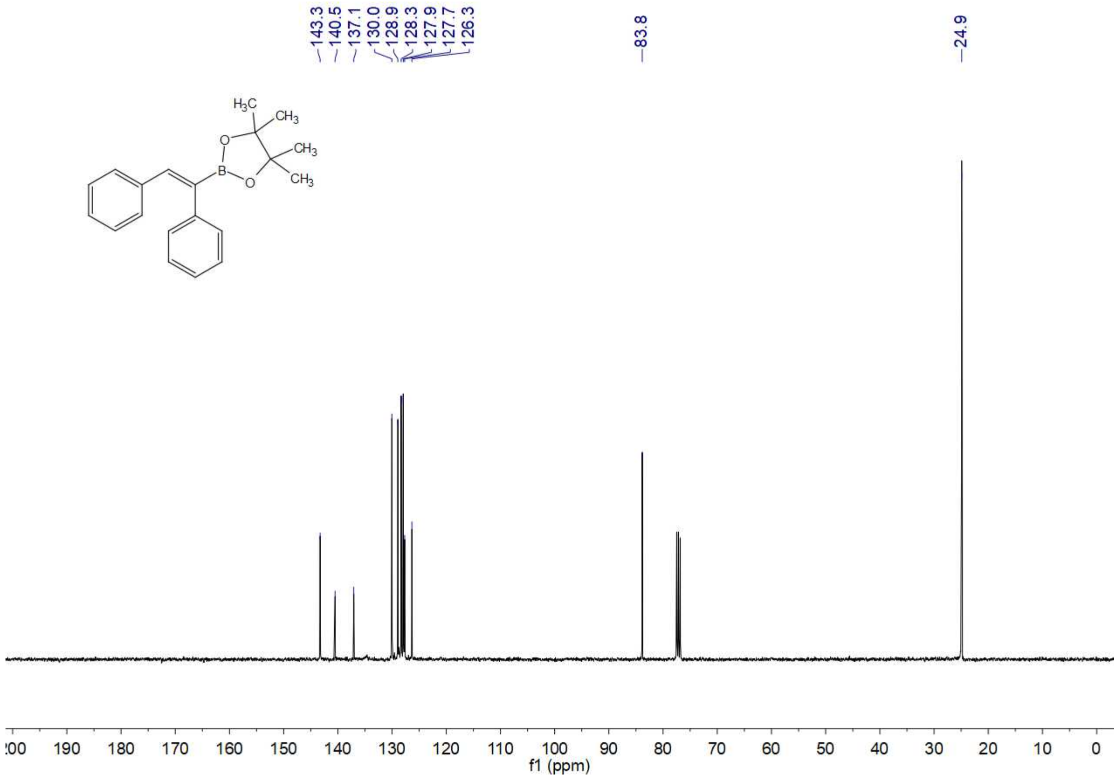

Supplementary Figure 33. **3w:** ^{1}H NMR (400 MHz, $CDCl_3$) (up) and ^{13}C NMR (101 MHz, $CDCl_3$) (down)

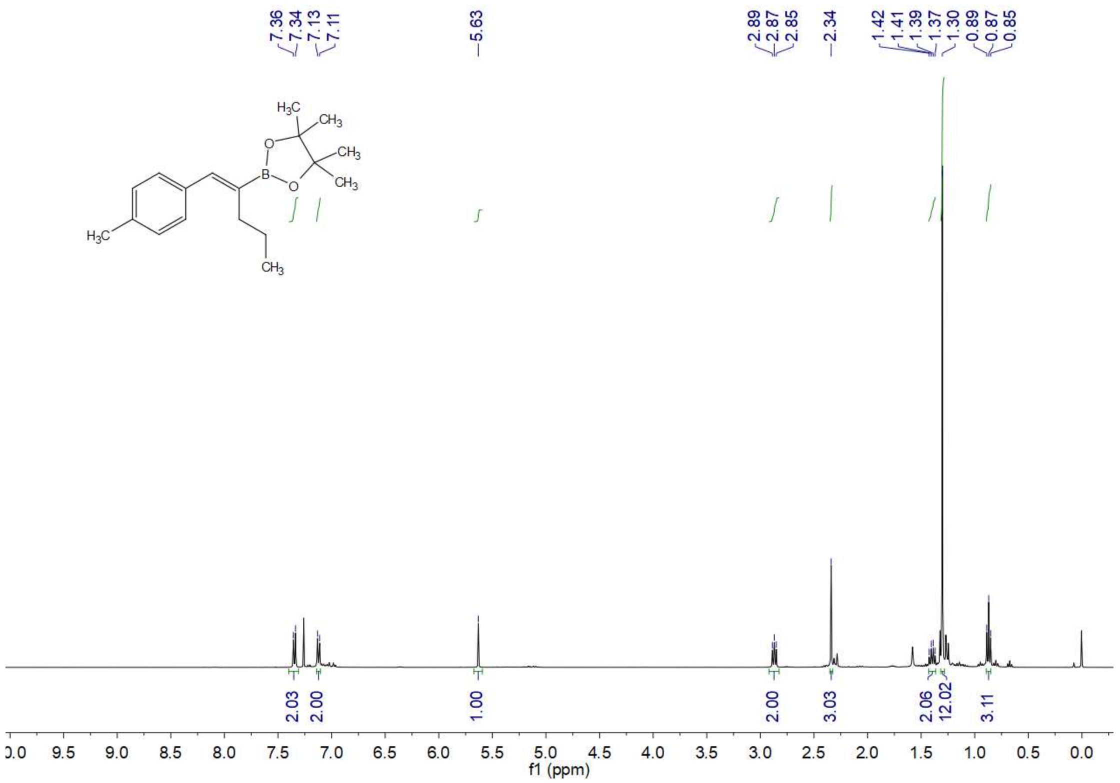

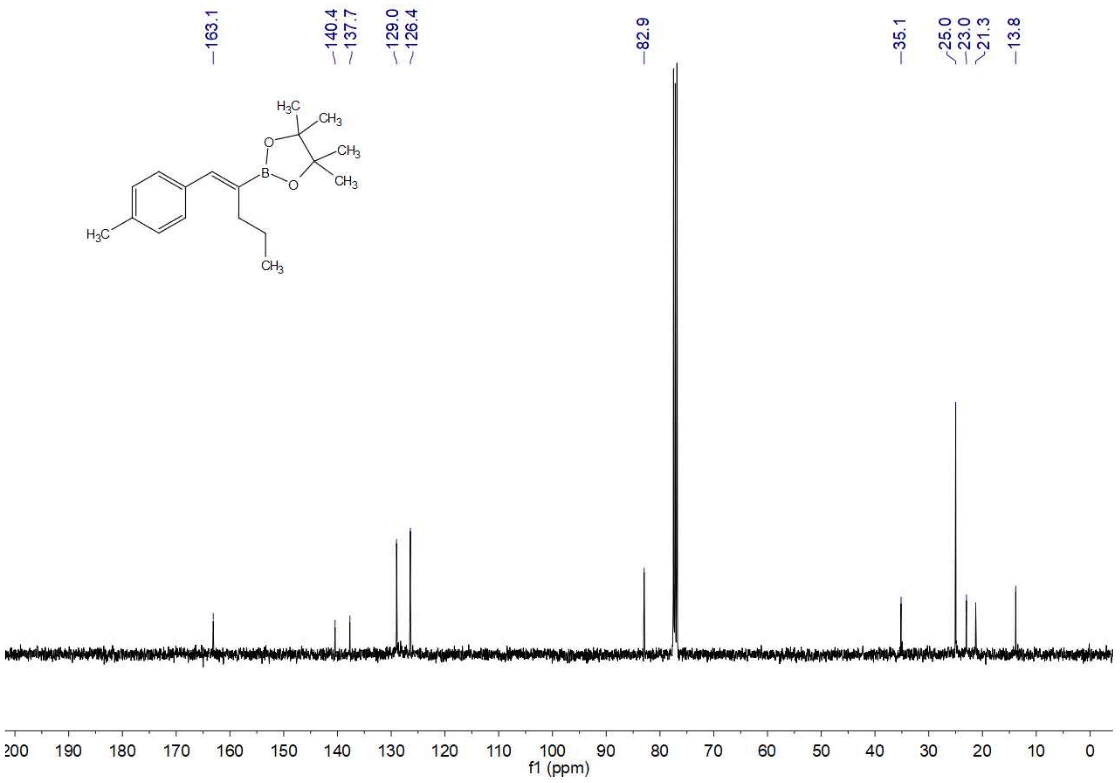

Supplementary Figure 34. **3x:** ^{1}H NMR (400 MHz, $CDCl_3$) (up) and ^{13}C NMR (101 MHz, $CDCl_3$) (down)

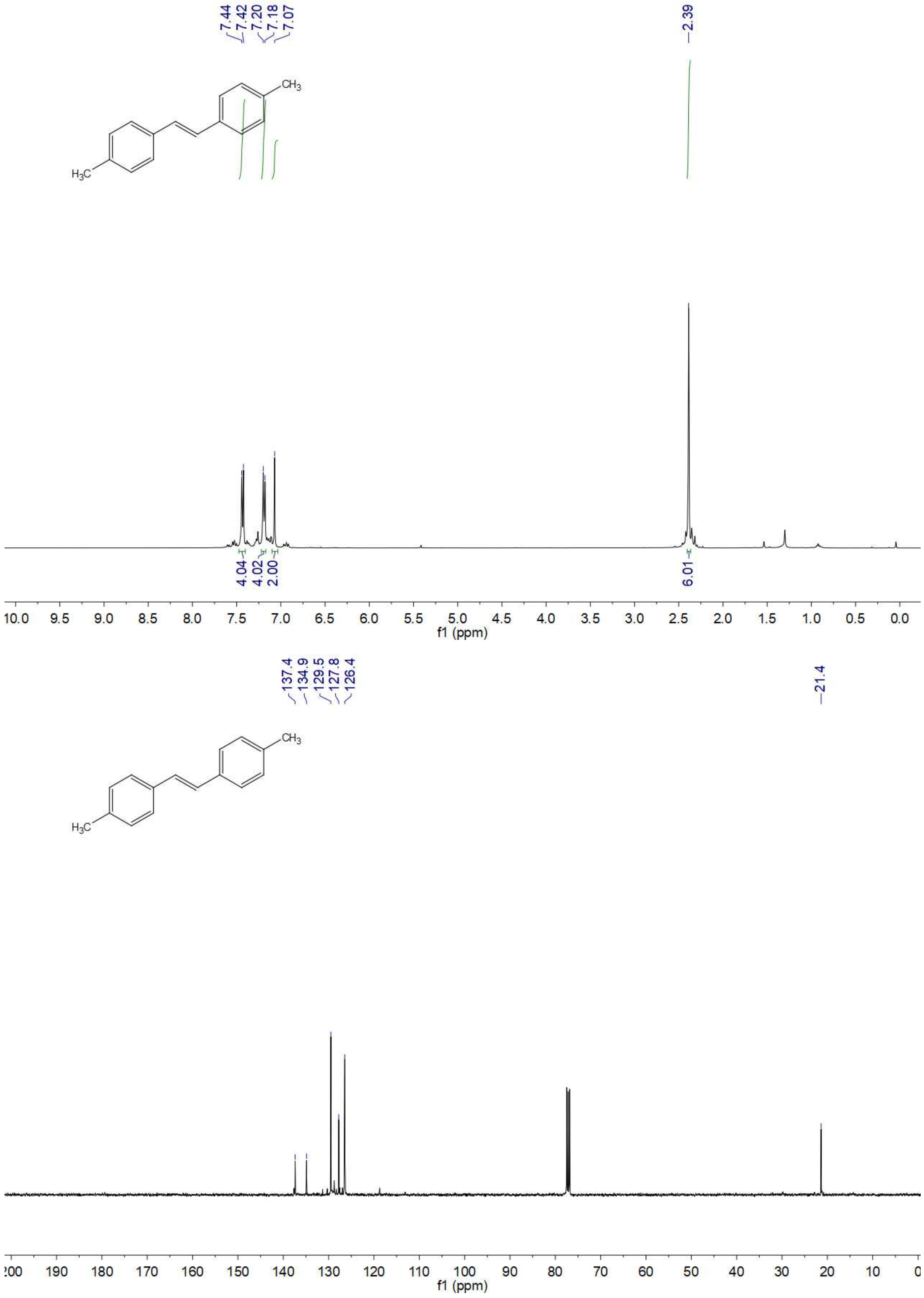

Supplementary Figure 35. **5a:** ^{1}H NMR (400 MHz, $CDCl_3$) (up) and ^{13}C NMR (101 MHz, $CDCl_3$) (down)

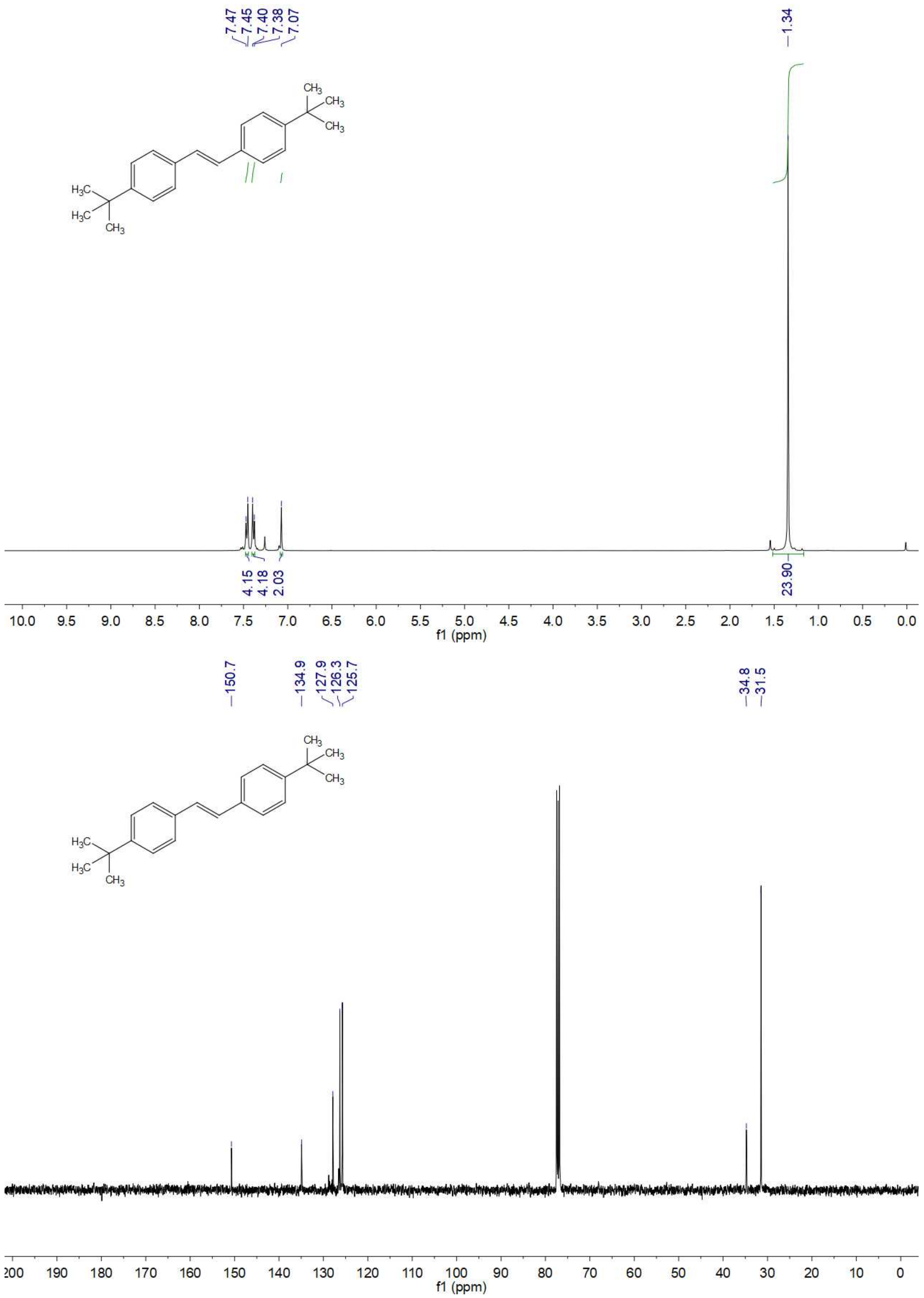

Supplementary Figure 36. **5b:** ^{1}H NMR (400 MHz, $CDCl_3$) (up) and ^{13}C NMR (101 MHz, $CDCl_3$) (down)

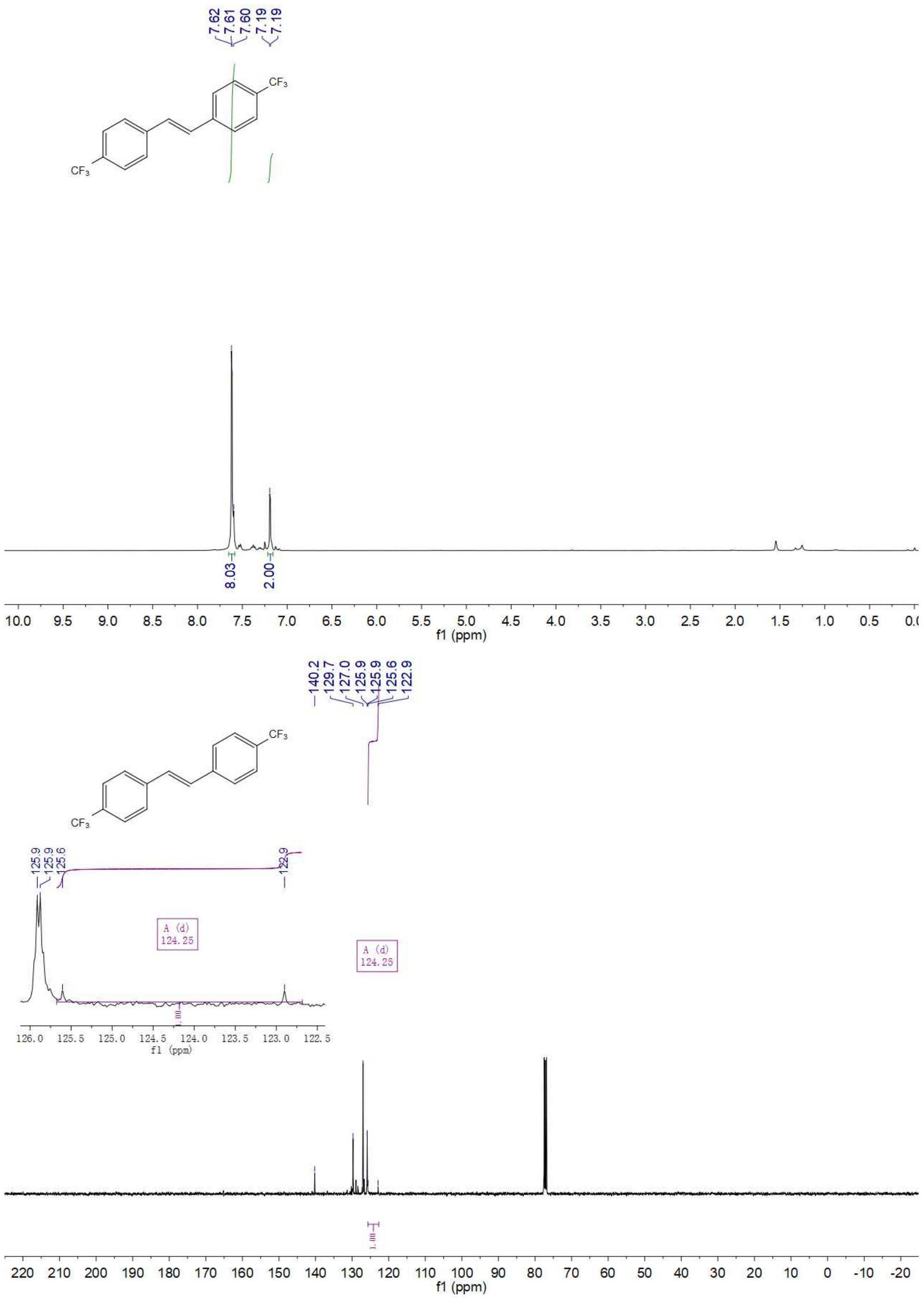

Supplementary Figure 37. **5c:** ^{1}H NMR (400 MHz, $CDCl_3$) (up) and ^{13}C NMR (101 MHz, $CDCl_3$) (down)

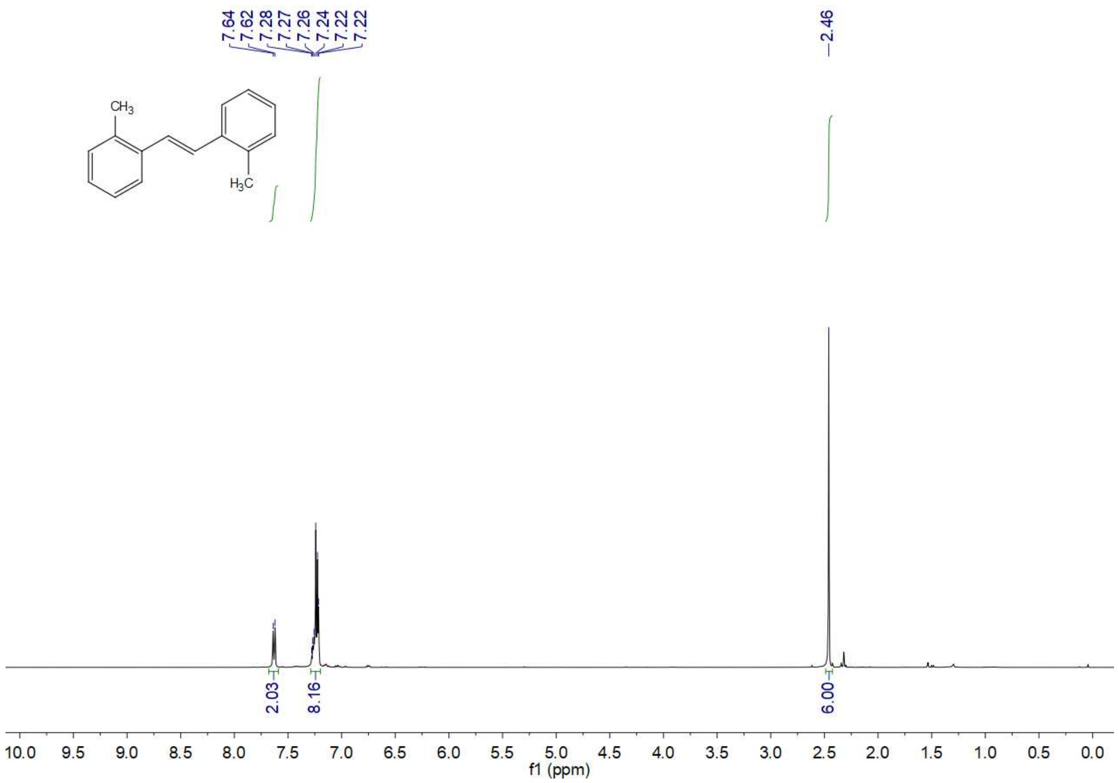

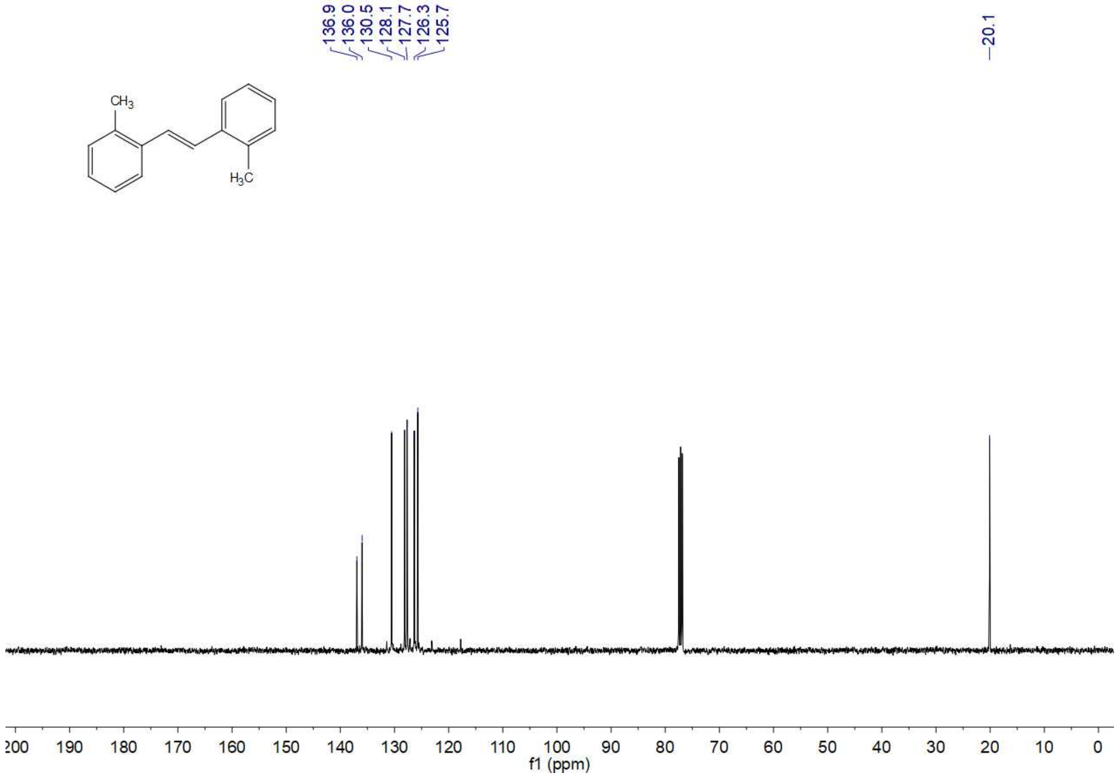

Supplementary Figure 38. **5d:** ^{1}H NMR (400 MHz, $CDCl_3$) (up) and ^{13}C NMR (101 MHz, $CDCl_3$) (down)

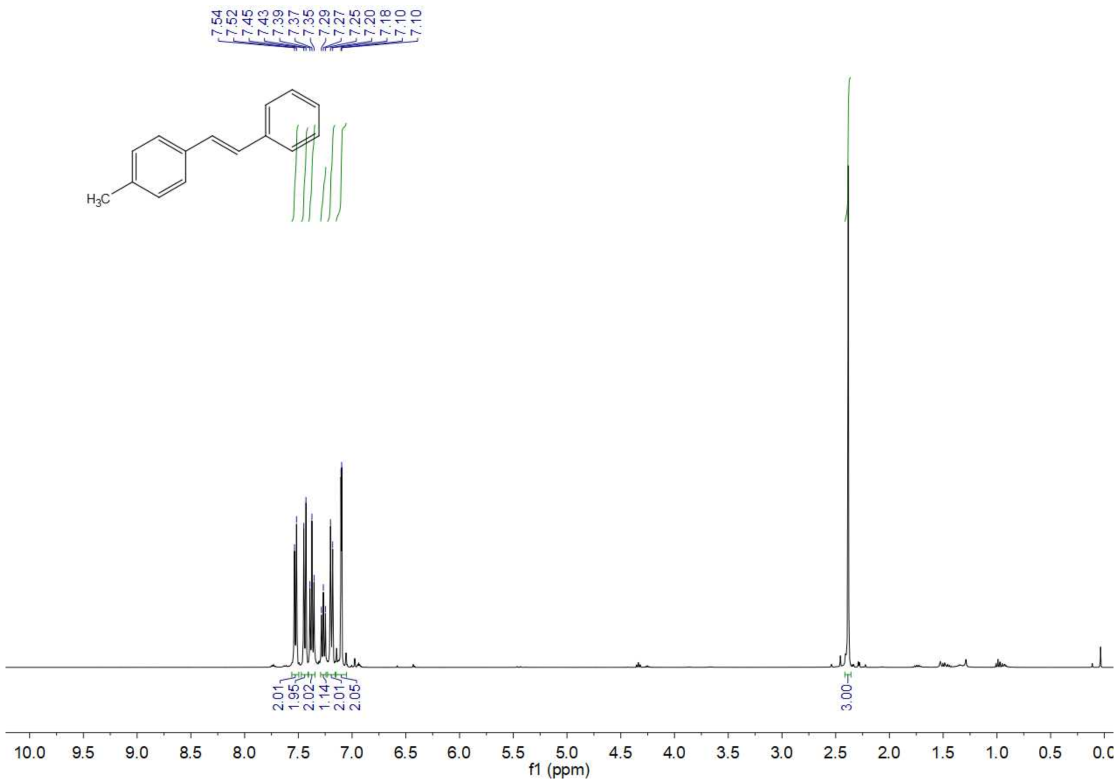

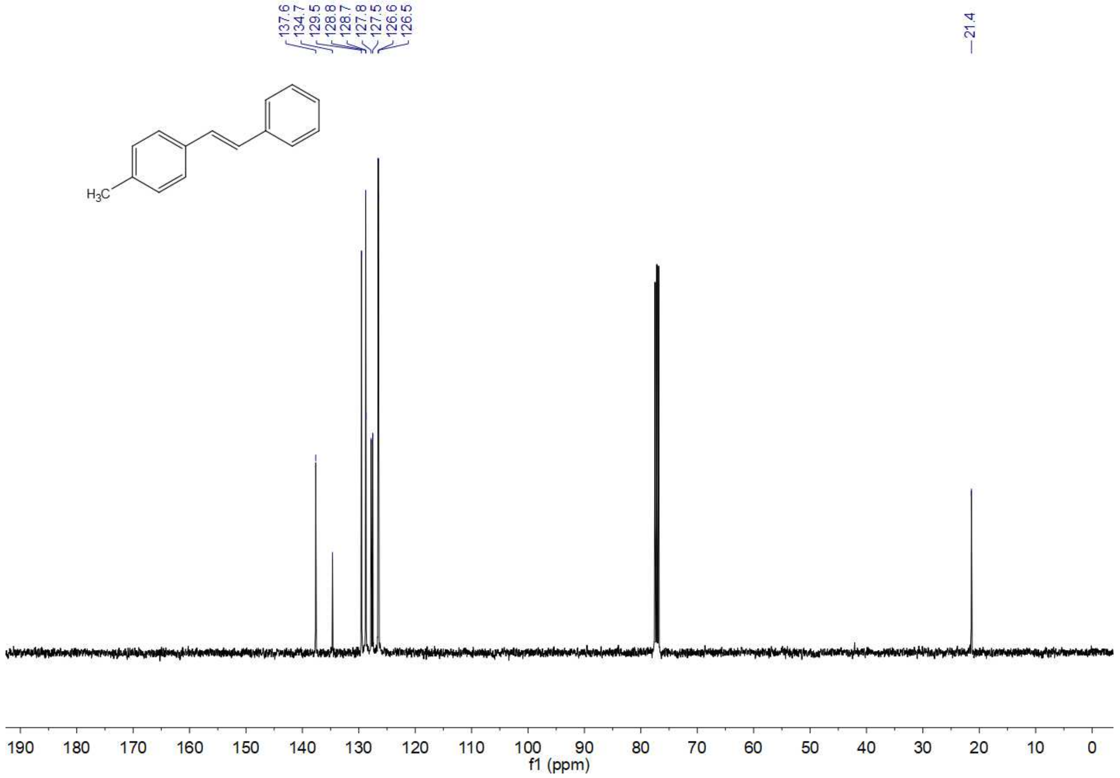

Supplementary Figure 39. **5e:** ^{1}H NMR (400 MHz, $CDCl_3$) (up) and ^{13}C NMR (101 MHz, $CDCl_3$) (down)

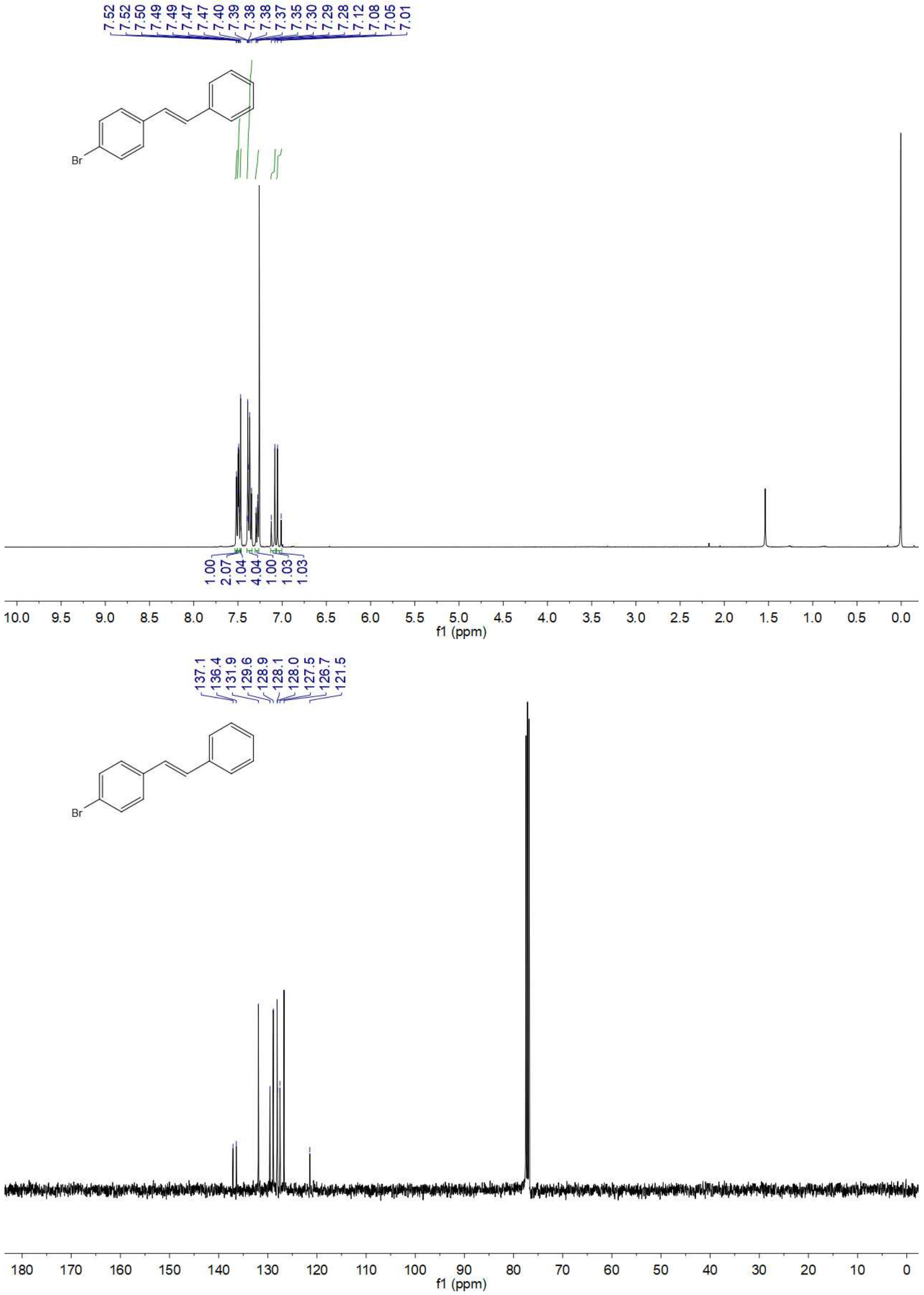

Supplementary Figure 40. **5f:** ^{1}H NMR (400 MHz, $CDCl_3$) (up) and ^{13}C NMR (101 MHz, $CDCl_3$) (down)

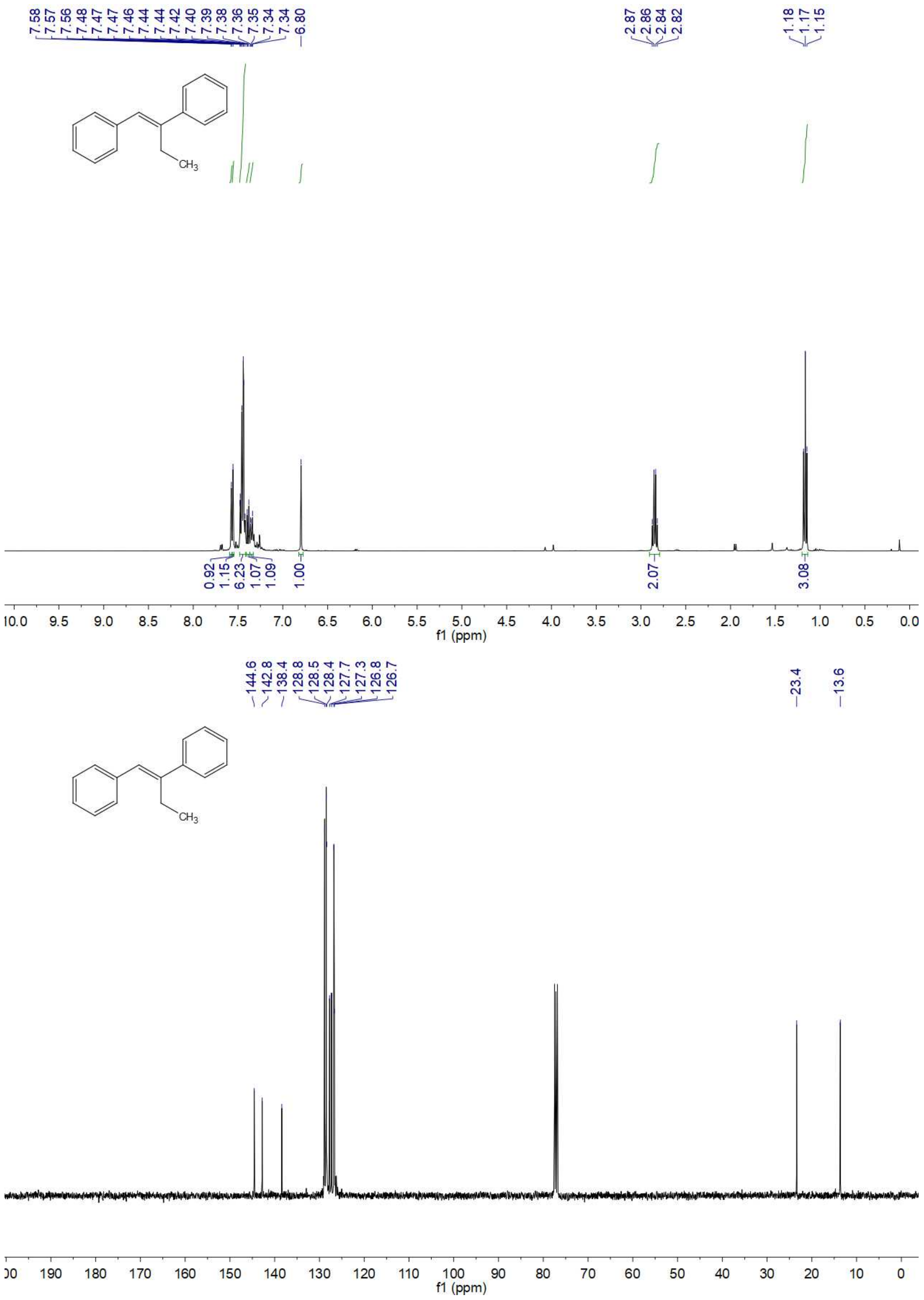

Supplementary Figure 41. **5g:** ^{1}H NMR (400 MHz, $CDCl_3$) (up) and ^{13}C NMR (101 MHz, $CDCl_3$) (down)

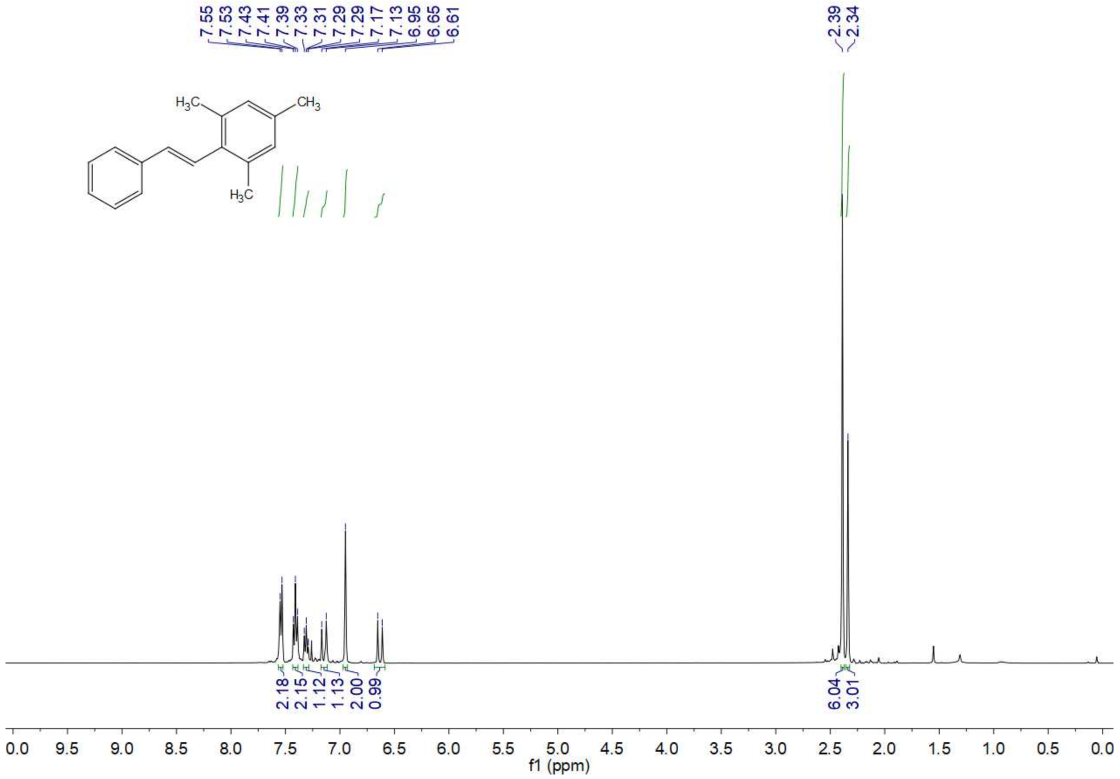

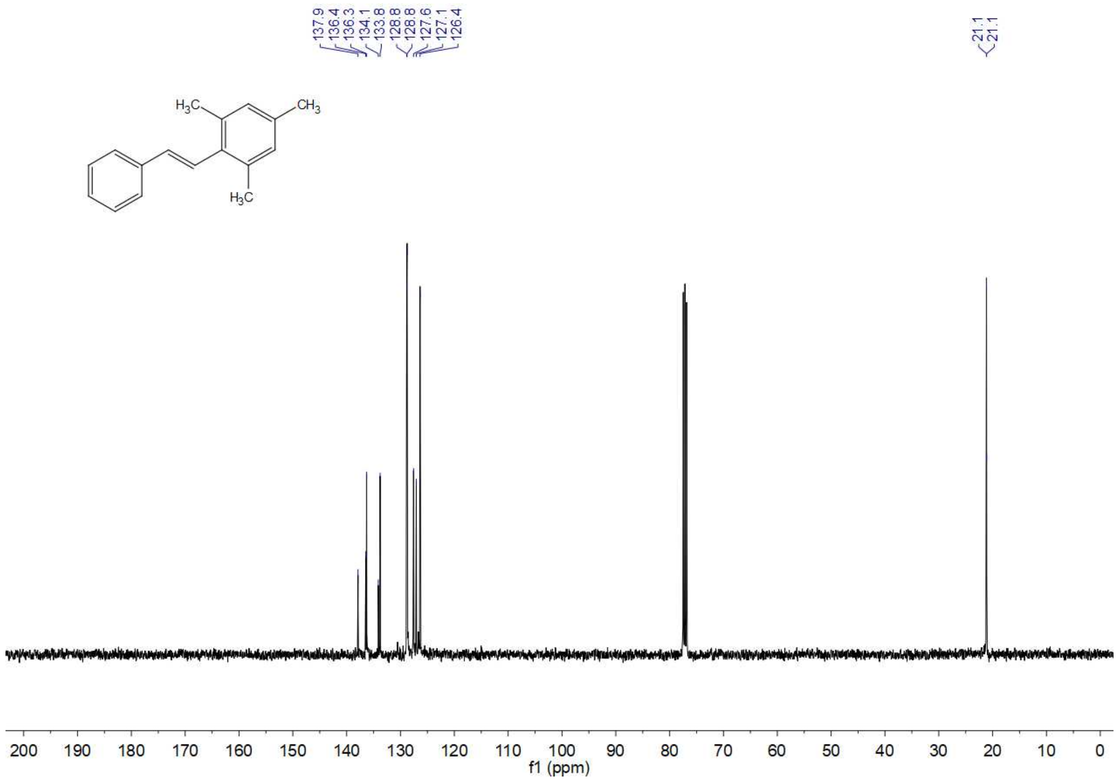

Supplementary Figure 42. **5h:** ^{1}H NMR (400 MHz, $CDCl_3$) (up) and ^{13}C NMR (101 MHz, $CDCl_3$) (down)

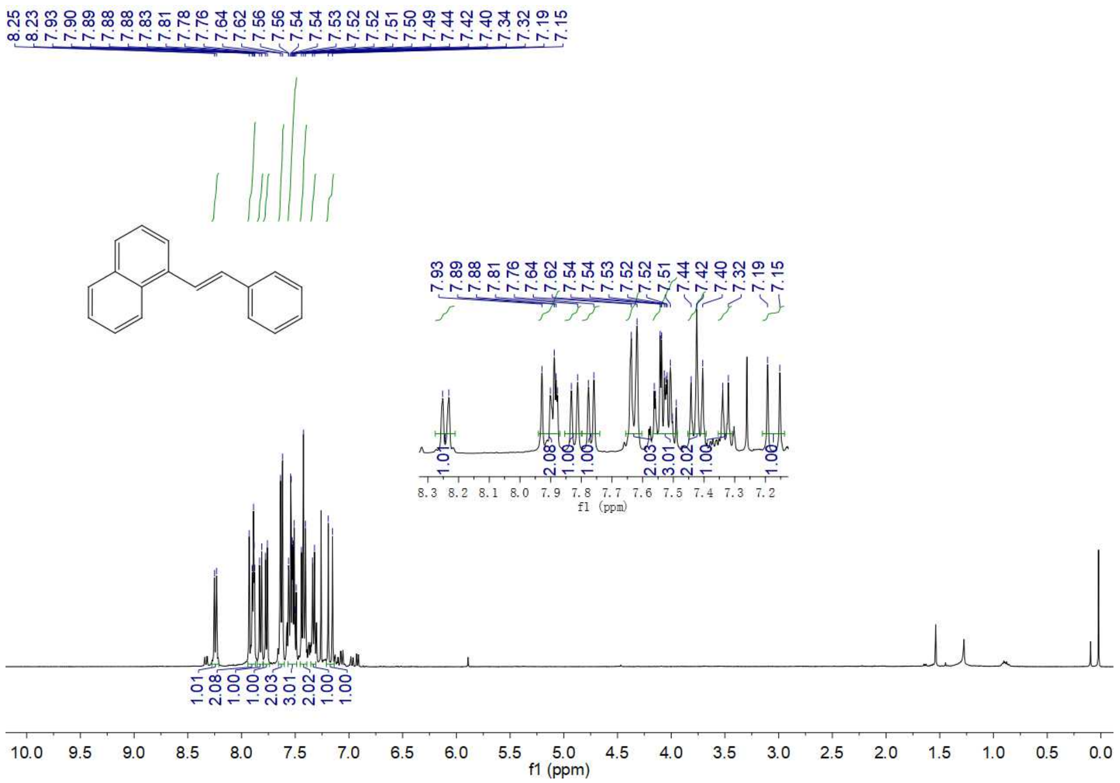

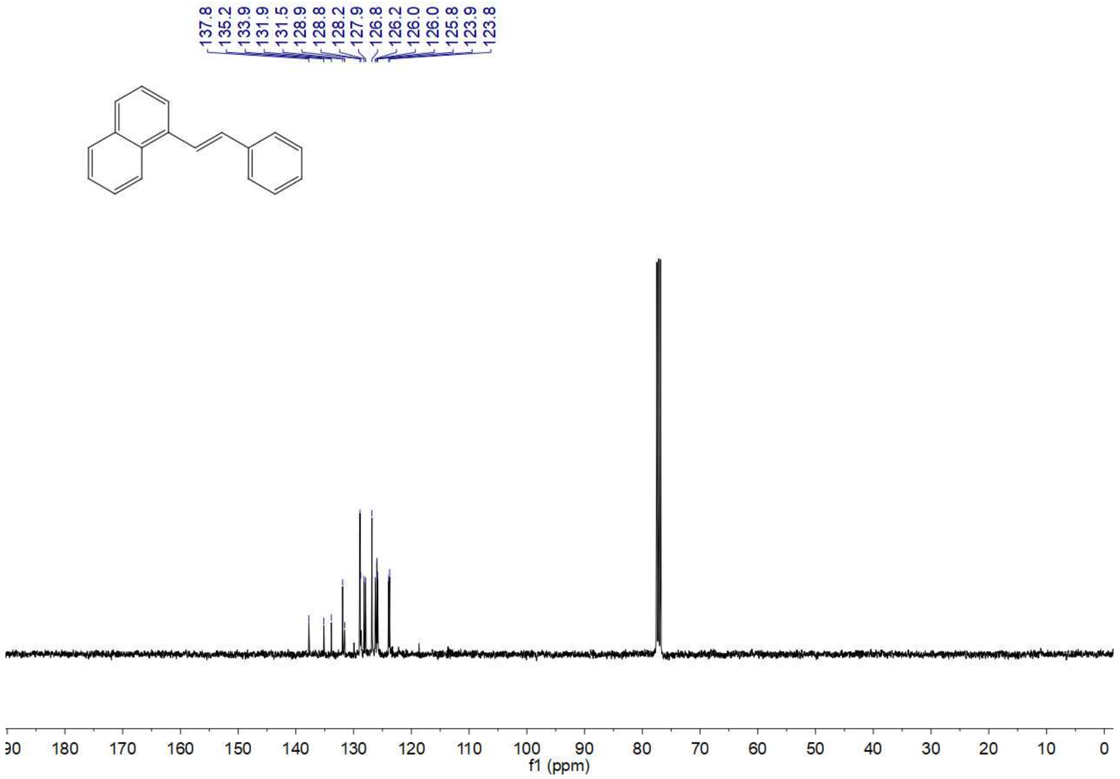

Supplementary Figure 43. **5i:** ^{1}H NMR (400 MHz, $CDCl_3$) (up) and ^{13}C NMR (101 MHz, $CDCl_3$) (down)

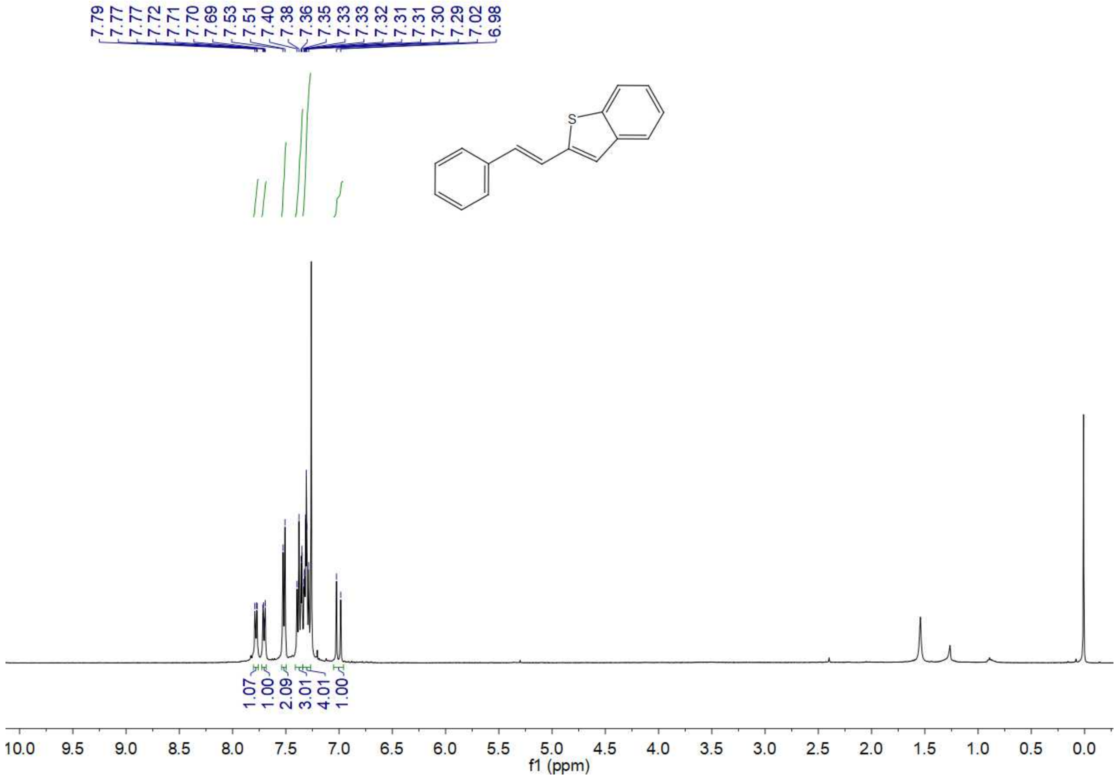

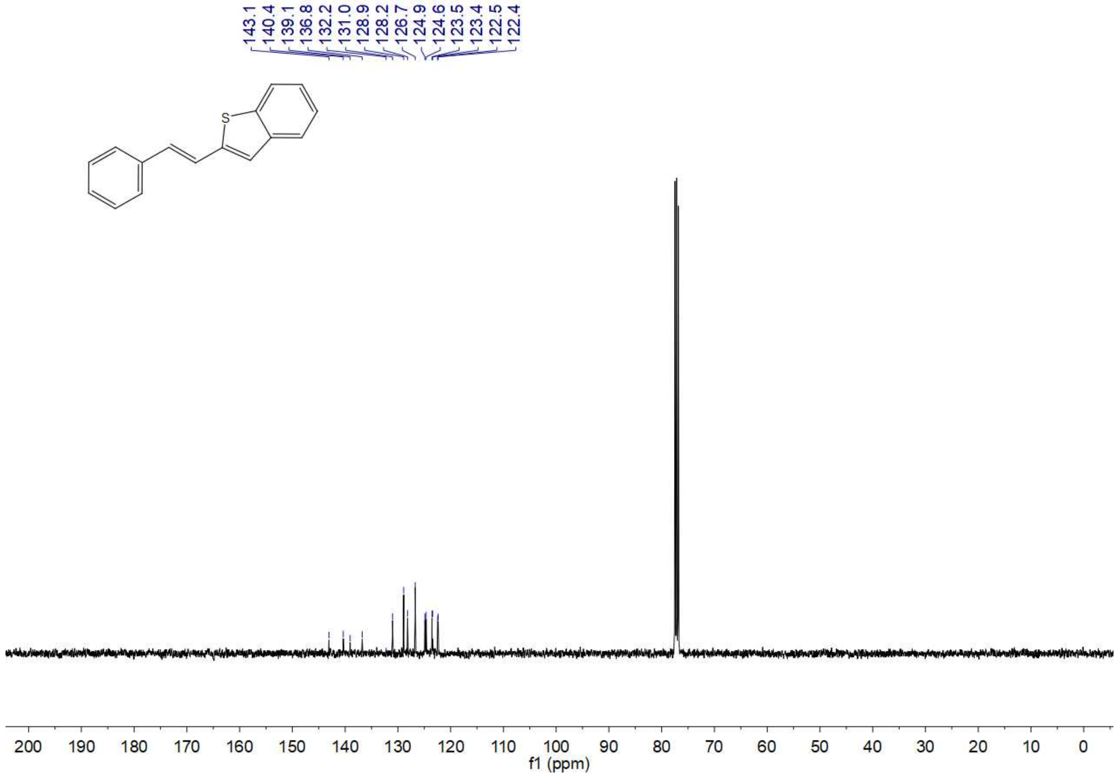

Supplementary Figure 44. **5j:** ^{1}H NMR (400 MHz, $CDCl_3$) (up) and ^{13}C NMR (101 MHz, $CDCl_3$) (down)

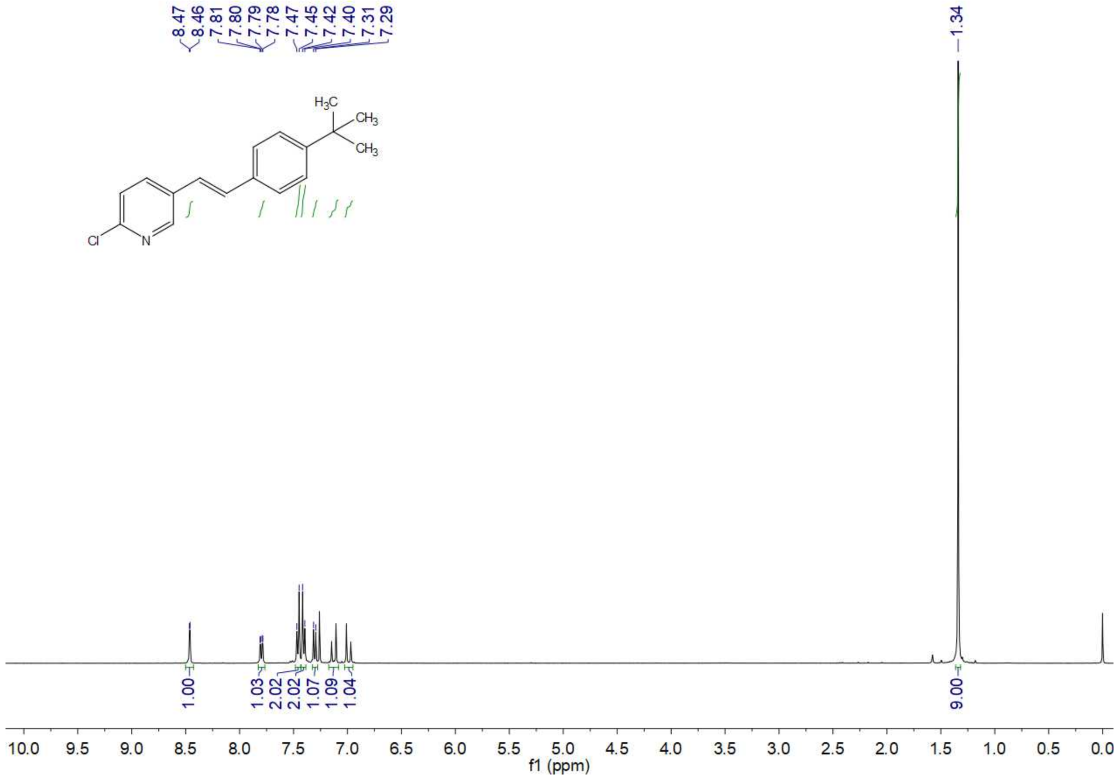

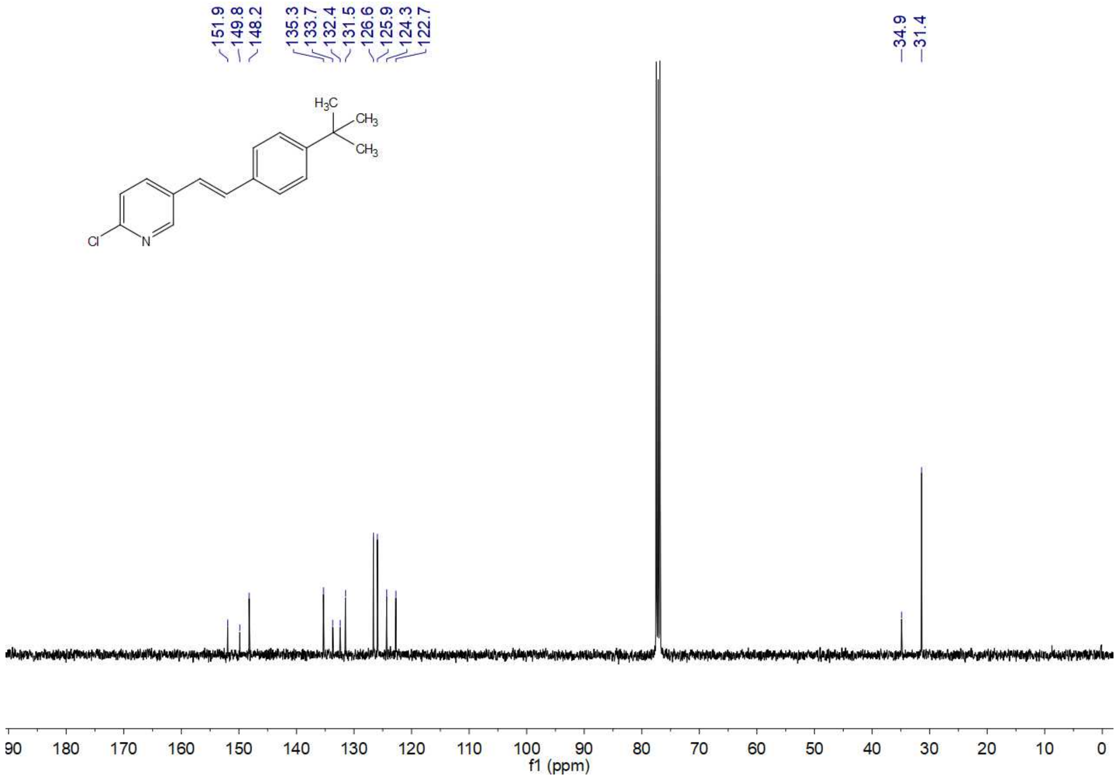

Supplementary Figure 45. **5k:** ^{1}H NMR (400 MHz, $CDCl_3$) (up) and ^{13}C NMR (101 MHz, $CDCl_3$) (down)

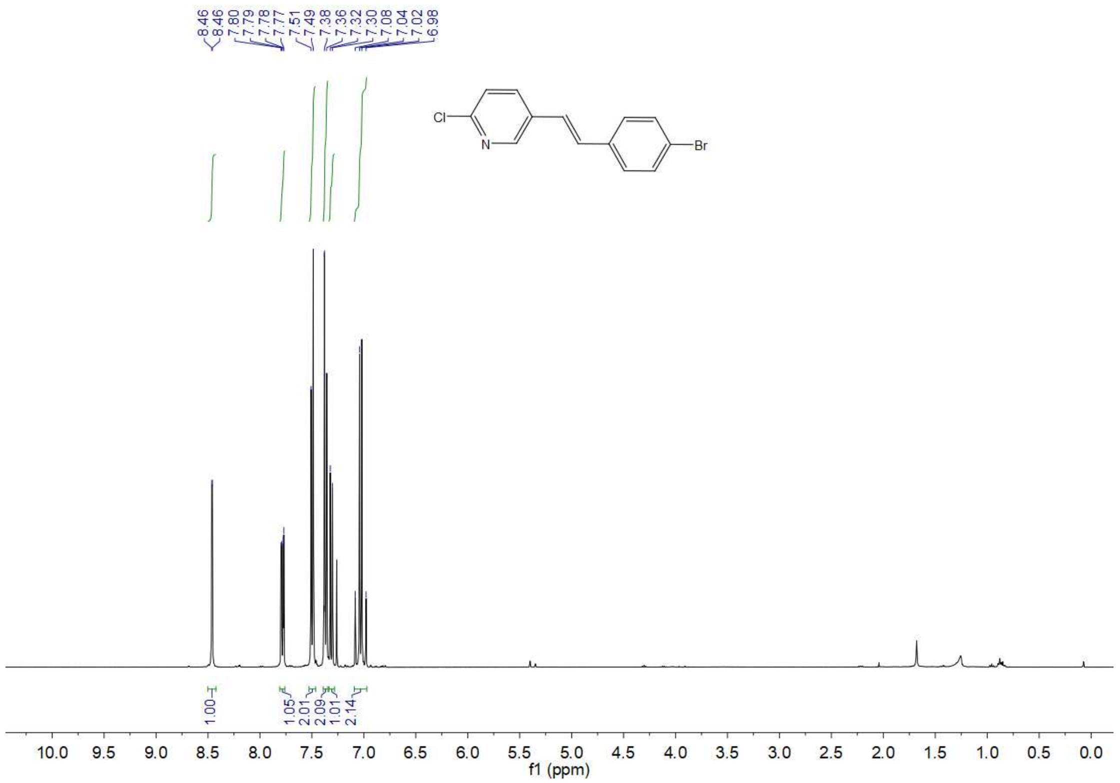

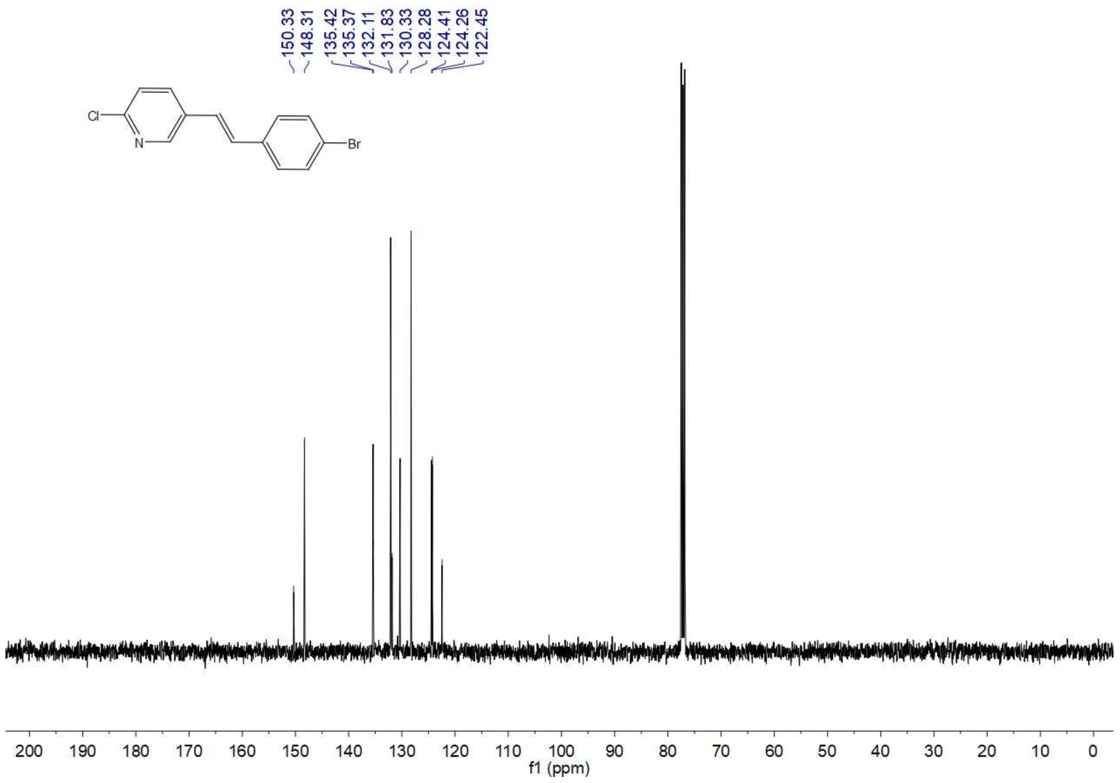

Supplementary Figure 46. **5l:** ^{1}H NMR (400 MHz, $CDCl_3$) (up) and ^{13}C NMR (101 MHz, $CDCl_3$) (down)

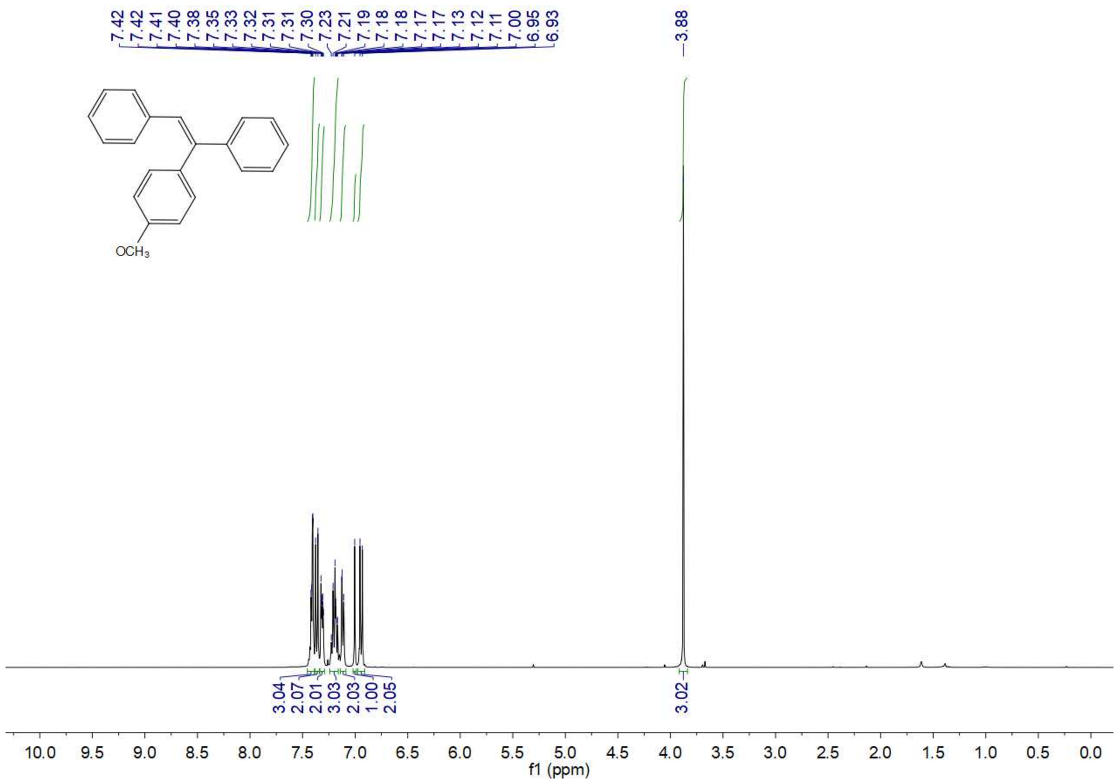

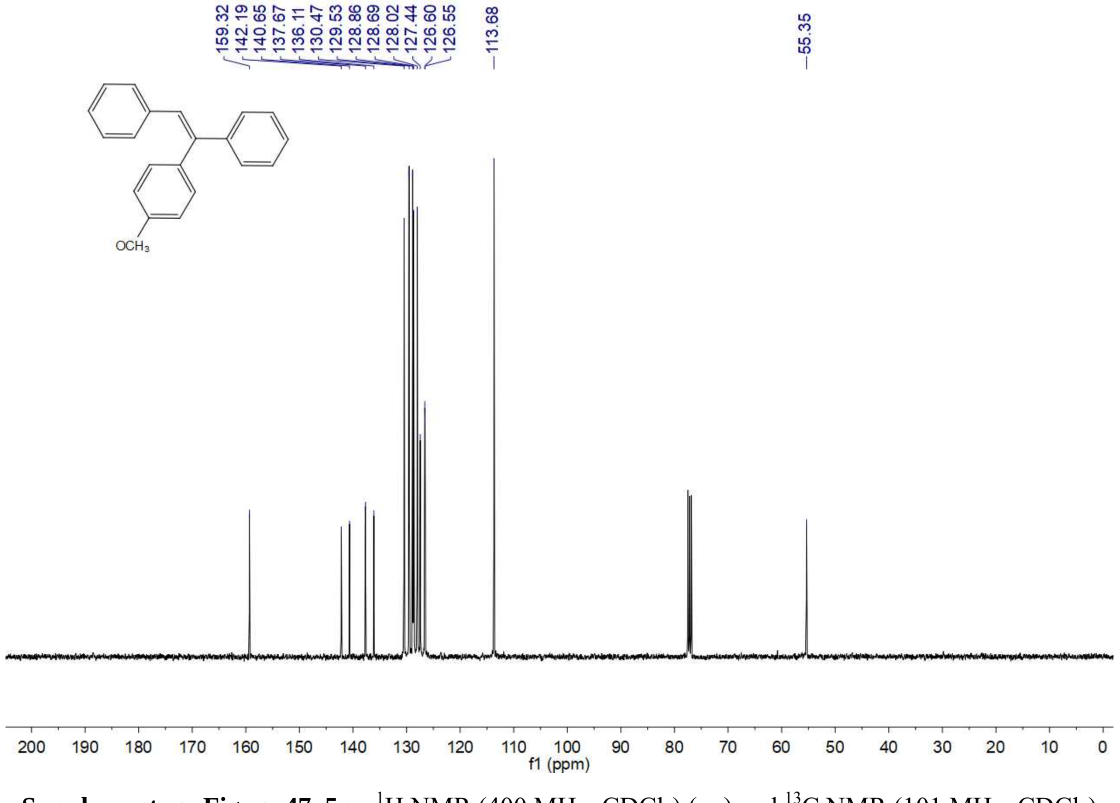

Supplementary Figure 47. **5m:** ^{1}H NMR (400 MHz, $CDCl_3$) (up) and ^{13}C NMR (101 MHz, $CDCl_3$) (down)

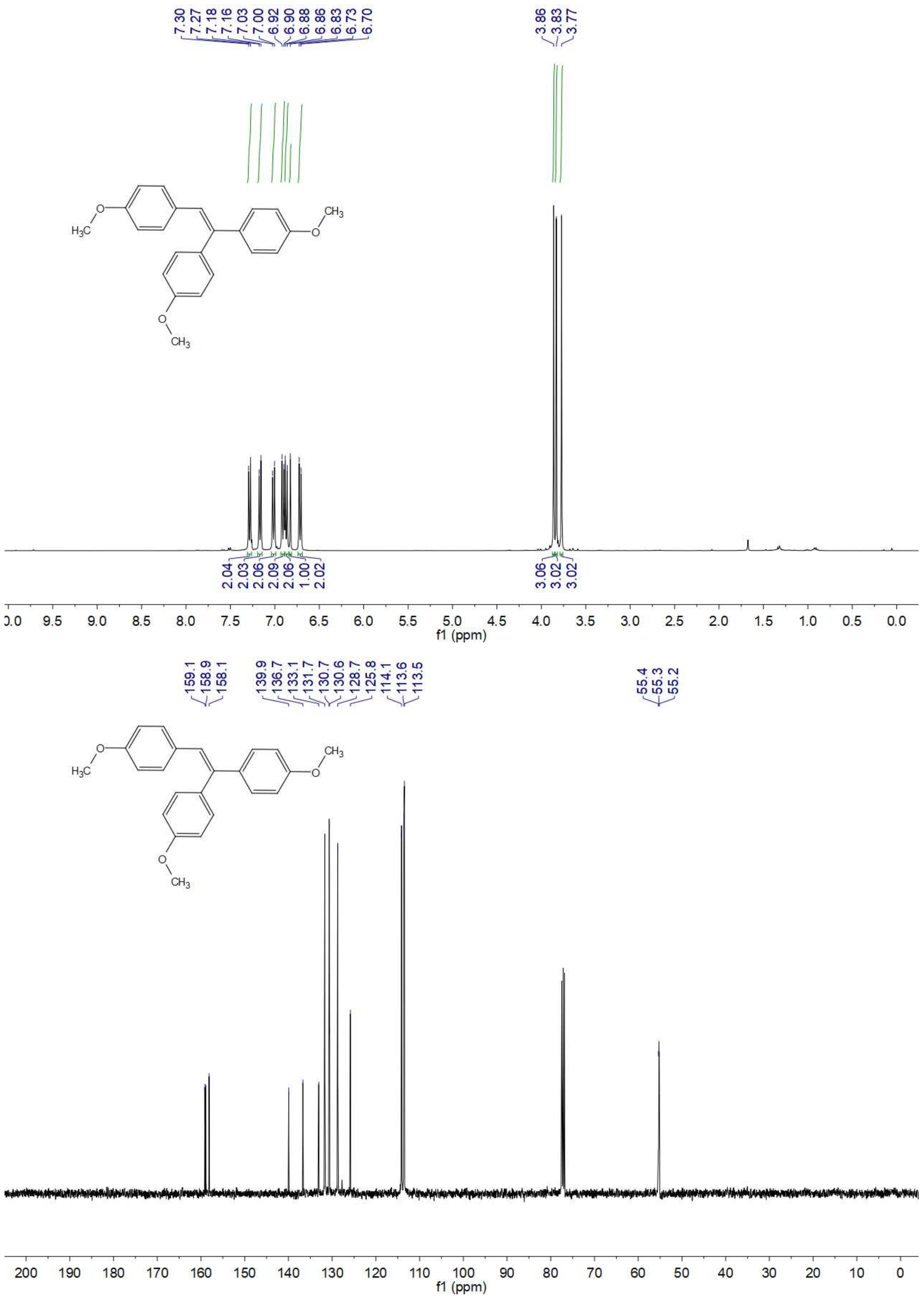

Supplementary Figure 48. **5n:** ^{1}H NMR (400 MHz, $CDCl_3$) (up) and ^{13}C NMR (101 MHz, $CDCl_3$) (down)

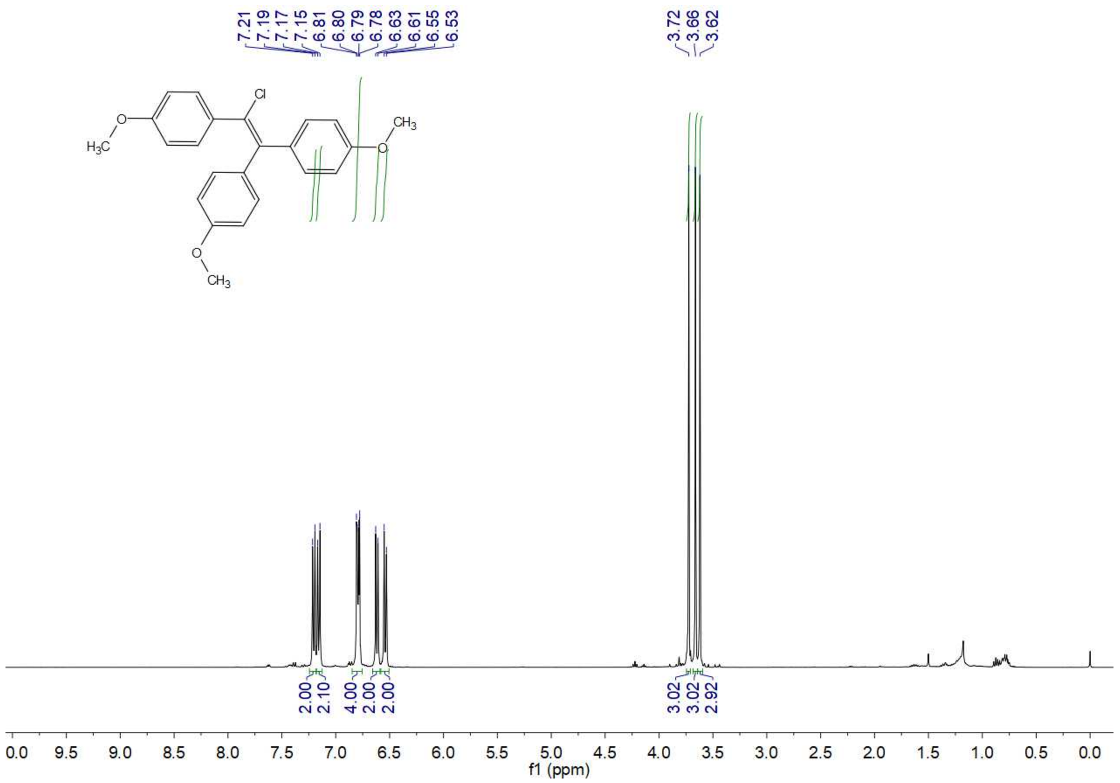
7.21 7.19 7.17 7.15 6.81 6.80 6.79 6.78 6.63 6.61 6.55 6.53
3.72 3.66 3.62
Cl
H_3C
O
CH_3
2.00 2.10 4.00 2.00 2.00
3.02 3.02 2.92
0.0 9.5 9.0 8.5 8.0 7.5 7.0 6.5 6.0 5.5 5.0 4.5 4.0 3.5 3.0 2.5 2.0 1.5 1.0 0.5 0.0
f1 (ppm)

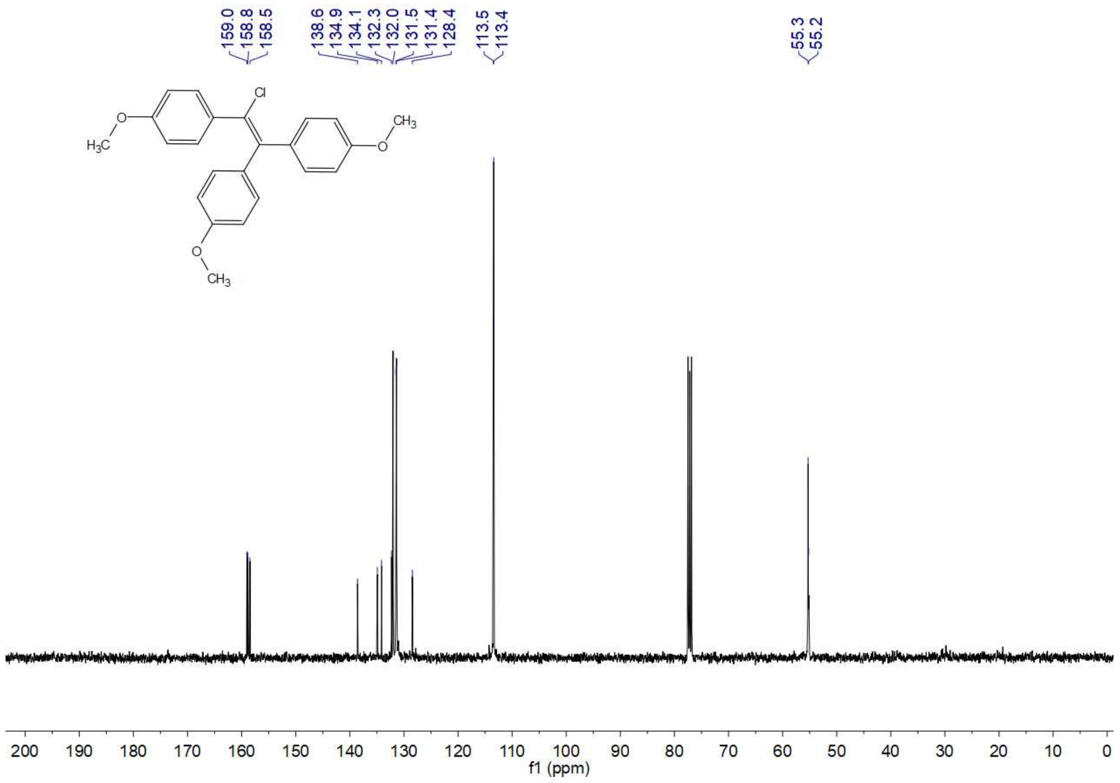
159.0 158.8 158.5
138.6 134.9 134.1 132.3 132.0 131.5 131.4 128.4
113.5 113.4
55.3 55.2
Cl
H_3C
O
CH_3
200 190 180 170 160 150 140 130 120 110 100 90 80 70 60 50 40 30 20 10 0
f1 (ppm)

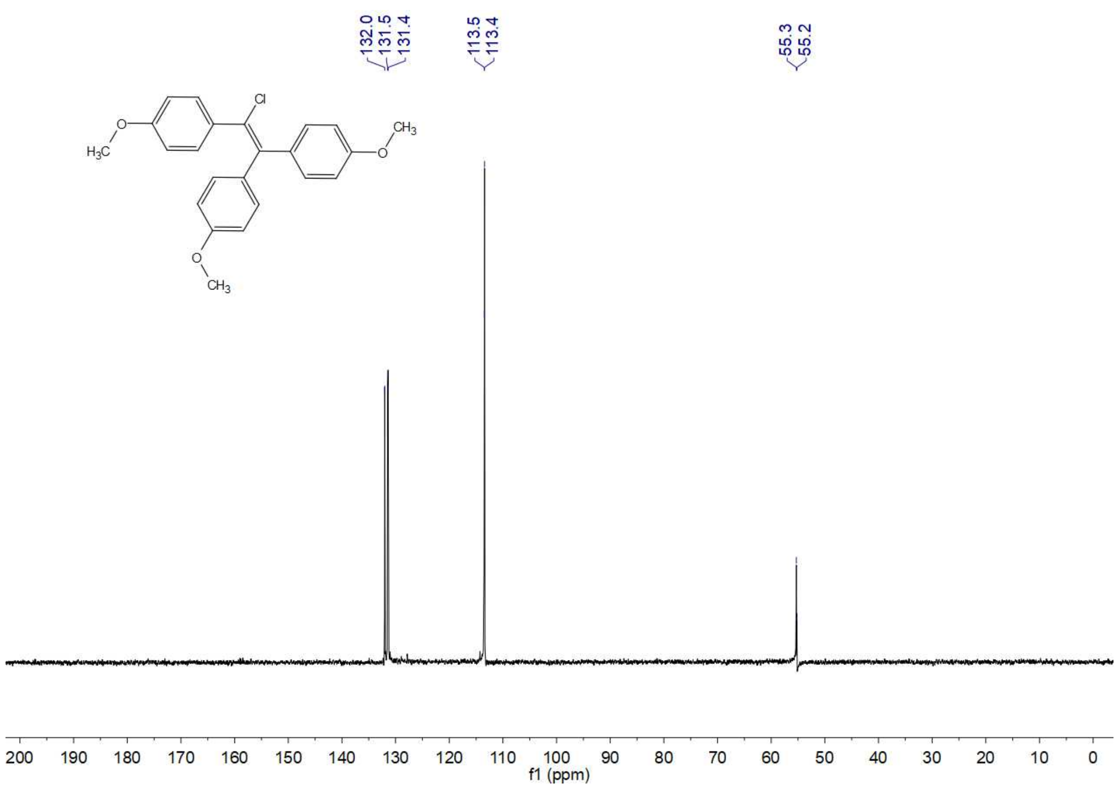

Supplementary Figure 49. The compound **10:** ^{1}H NMR (400 MHz, $CDCl_3$) (up) and ^{13}C NMR (101 MHz, $CDCl_3$) (middle) DEPT-135: (101 MHz, $CDCl_3$) (down)

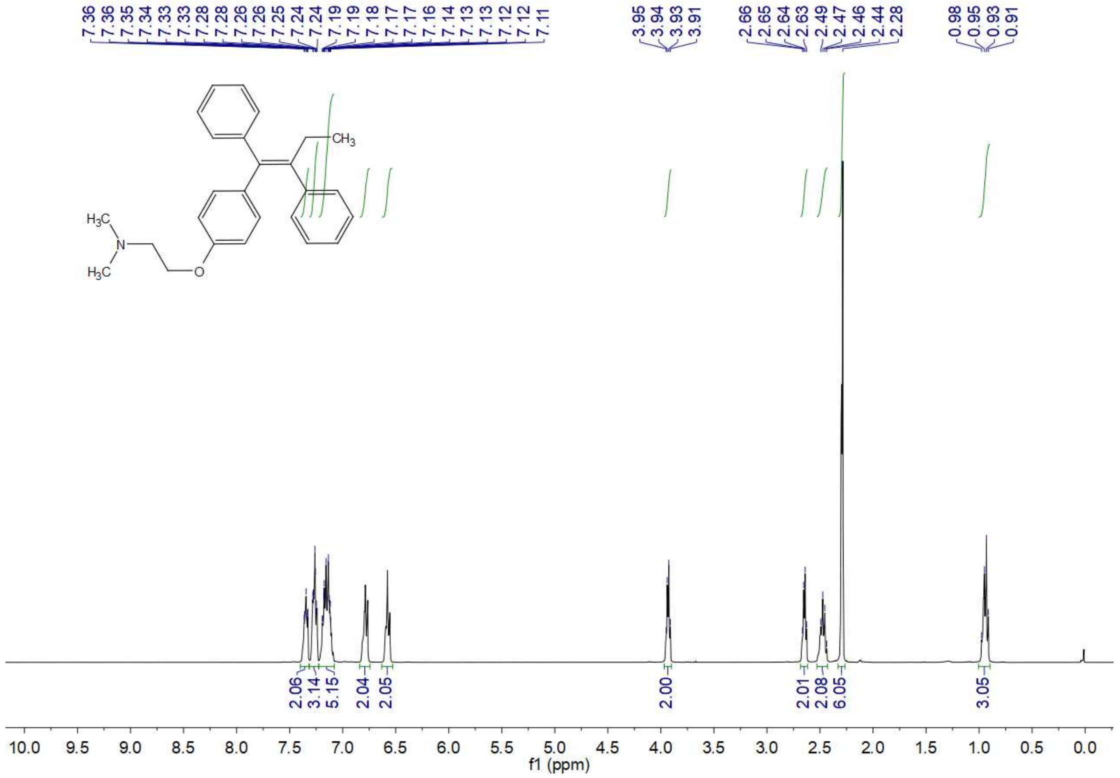

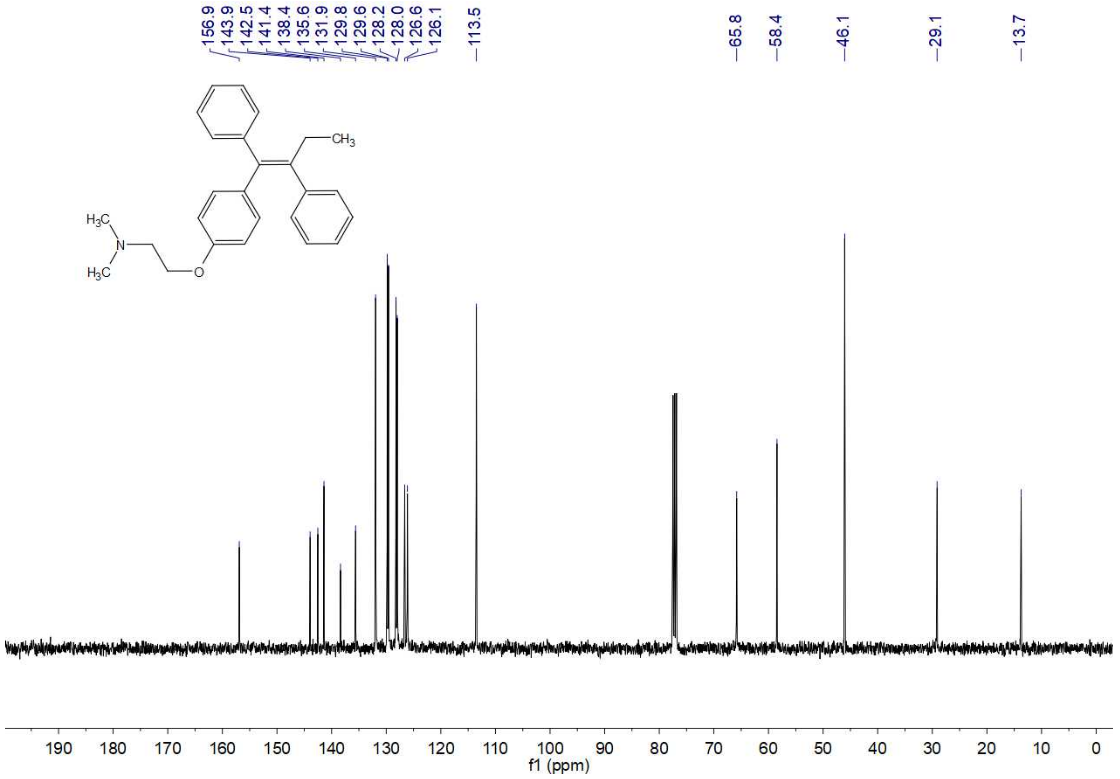

Supplementary Figure 50. **Tamoxifen (12):** ^{1}H NMR (400 MHz, $CDCl_3$) (up) and ^{13}C NMR (101 MHz, $CDCl_3$) (down)

Supplementary References

[1] M. Bielawski, M. Zhu, B. Olofsson, *Adv. Synth. Catal.* **2007**, 349, 2610.

[2] V. V.Zhdankin, P. J. Stang, *Chem. Rev*. **2008**, 108, 5299.

[3] E. A. Merritt, B. Olofsson, *Angew. Chem.* **2009**, 121, 9214; *Angew. Chem. Int. Ed.* **2009**, 48, 9052.

[4] E. Skucas, D. W. C MacMillan, *J. Am. Chem. Soc*. **2012**, 134, 9090.

[5] D. W. Johnson, G. Phillipou, C. J. Seaborn, *Aust. J. Chem.* **1980,** 33, 461.

[6] C. M. Nunes, J. Limberger, S. Poersh, M. Seferin, A. L. Monteiro, *Synthesis* **2009,** 2761

[7] J. Wen, J. Zhang, X. Q. Yu, *J. Org. Chem.* **2012**, 77, 766−771.

[8] J. Peng, M. Li, C. J. Xi, C. Chen, *Angew. Chem. Int. Ed.* **2013**, 52, 7574-7578.

[9] A. D. Becke, *Phys. Rev. A.* **1988**, *38*, 3098−3100.

[10] C. T. Lee, W. T. Yang, R. G. Parr, *Phys. Rev. B.* **1988**, *37*, 785−789.

[11] A. D. Becke, *J. Chem. Phys.* **1993**, *98*, 5648−5652.

[12] M. J. Frisch, G. W. Trucks, H. B. Schlegel, G. E. Scuseria, M. A. Robb, J. R. Cheeseman, G. Scalmani, O. Farkas, J. B. Foresman, J. V. Ortiz, J. Cioslowski, D. J. Fox, Gaussian 09, revision C.01; Gaussian, Inc.: Wallingford, CT, 2009.

[13] F. Weigend, R. Ahlrichs, *Phys. Chem. Chem. Phys.* **2005**, *7*, 3297−3305.

[14] A. V. Marenich, C. J. Cramer, D. G. Truhlar, *J. Phys. Chem. B* **2009**, *113*, 6378−6396.
