# Supplementary material for: Wet carbonate-promoted radical arylation of vinyl pinacolboronates with diaryliodonium salts yields substituted olefins
Source: Commun Chem. 2020 Jul 22;3:92. doi: 10.1038/s42004-020-00343-8 (PMC9814134; doi:10.1038/s42004-020-00343-8)
Supplement: Supplementary file 2 — Description of Additional Supplementary Files [file 42004_2020_343_MOESM2_ESM.pdf]

Description of Additional Supplementary Files

File Name: Supplementary Data 1
Description: It's the Cartesian coordinates file at the level of 3LYP/def2TZVP(SMD)//B3LYP/def2SV (P) for compounds including Vinyl Pinacol Boronates(2a)\ PhIPh\ PhIPh-TfO\ PhIPh-K_2CO_3\ PhIPh-KCO_3^{-} \PhIPh-CO_3^{2-}\ Phenyl-raidcal\ decomposed Ph_2I^{+} -CO_3^{2-}\ decomposed Ph_2I^{+} - KCO_3^{-}\ PhI-TfO\ PhI-K_2CO_3\ PhI- CO_3^{2-}\ PhI\ Intermediate II\ Intermediate III\ Intermediate IV\ TS1\ Intermediate V\ TS2\ Intermediate VI\ TS3\ *trans*-phenylvinylboronate(3e)\ KCO_3^{-} \ HCO_3 - \ CO_3^{2-}\ Intermediate II-K\ Intermediate III-K\ Intermediate IV-K\ TS1-K\ Intermediate V-K\ TS2-K\ Intermediate VI-K\ TS3-K\ HCO_3^{-} -KCO_3^{-}
