# Supplementary material for: Wet carbonate-promoted radical arylation of vinyl pinacolboronates with diaryliodonium salts yields substituted olefins
Source: Commun Chem. 2020 Jul 22;3:92. doi: 10.1038/s42004-020-00343-8 (PMC9814134; doi:10.1038/s42004-020-00343-8)
Supplement: Supplementary file 3 — Supplementary Data 1 [file 42004_2020_343_MOESM3_ESM.pdf]

**DFT detail**

Coodrdinates

Vinyl Pinacol Boronates(**2a**)

| | | | |
|---|---|---|---|
| C | -6.46928200 | 2.42200700 | -0.42983200 |
| C | -6.12131800 | 2.00343000 | -1.91992700 |
| C | -6.79843200 | 1.22822300 | 0.47733200 |
| H | -6.82689300 | 1.57351300 | 1.52482900 |
| H | -7.77799400 | 0.78395600 | 0.23097900 |
| H | -6.02835100 | 0.44201900 | 0.40302800 |
| C | -7.54907200 | 3.49457900 | -0.29148000 |
| H | -8.51333300 | 3.14082400 | -0.69771700 |
| H | -7.69511100 | 3.73433900 | 0.77582600 |
| H | -7.26911100 | 4.42529700 | -0.80829600 |
| C | -6.77393500 | 0.70733000 | -2.39933500 |
| H | -7.87500900 | 0.79467600 | -2.39833000 |
| H | -6.44917000 | 0.49128700 | -3.43172200 |
| H | -6.48770900 | -0.15044100 | -1.77164500 |
| C | -6.37125300 | 3.12198600 | -2.94142400 |
| H | -5.92164500 | 2.83015400 | -3.90578500 |
| H | -7.44871400 | 3.29960600 | -3.10001500 |
| H | -5.90306400 | 4.06932600 | -2.62528500 |
| O | -5.21170700 | 2.96794700 | 0.03706500 |
| O | -4.68894600 | 1.80497900 | -1.85611000 |
| B | -4.21013300 | 2.48634200 | -0.76565100 |
| C | -2.69710800 | 2.69356400 | -0.46558000 |
| H | -2.41160600 | 3.22635100 | 0.45272500 |
| C | -1.72372100 | 2.24669600 | -1.27648000 |
| H | -1.96013800 | 1.70909600 | -2.20451500 |
| H | -0.65746000 | 2.39303800 | -1.05496900 |

PhIPh

| | | | |
|---|---|---|---|
| C | 3.27523400 | -1.27670100 | -1.21461600 |
| C | 2.18273300 | -0.40266500 | -1.23123600 |
| C | 1.66670700 | 0.00365900 | 0.00003000 |
| C | 2.18248800 | -0.40293000 | 1.23131100 |
| C | 3.27499000 | -1.27696700 | 1.21471900 |
| C | 3.81574500 | -1.71108900 | 0.00005900 |
| H | 3.70302600 | -1.61318500 | -2.16179500 |
| H | 1.76418900 | -0.05900800 | -2.17850200 |
| H | 1.76375500 | -0.05947500 | 2.17856700 |
| H | 3.70259000 | -1.61365600 | 2.16191100 |
| H | 4.66995700 | -2.39173400 | 0.00007000 |
| I | 0.00000000 | 1.36366800 | -0.00000100 |
| C | -1.66671700 | 0.00367100 | -0.00002800 |

| | | | |
|---|---|---|---|
| C | -2.18274000 | -0.40265800 | 1.23123800 |
| C | -2.18249100 | -0.40292500 | -1.23131000 |
| C | -3.27523000 | -1.27670700 | 1.21461600 |
| H | -1.76420200 | -0.05899100 | 2.17850300 |
| C | -3.27498300 | -1.27697300 | -1.21471900 |
| H | -1.76375900 | -0.05946300 | -2.17856300 |
| C | -3.81573800 | -1.71109500 | -0.00006000 |
| H | -3.70302000 | -1.61319600 | 2.16179400 |
| H | -3.70258000 | -1.61366600 | -2.16191200 |
| H | -4.66993900 | -2.39175400 | -0.00007200 |

PhIPh-TfO

| | | | |
|---|---|---|---|
| C | 4.57164200 | 1.89176500 | -0.91041500 |
| C | 3.25680500 | 1.45874300 | -1.12321200 |
| C | 2.48480800 | 1.05553300 | -0.02811200 |
| C | 3.00123700 | 1.08766900 | 1.27235500 |
| C | 4.31737400 | 1.51700100 | 1.47175300 |
| C | 5.10088000 | 1.91827800 | 0.38337400 |
| H | 5.18138400 | 2.20804100 | -1.76230800 |
| H | 2.84598700 | 1.43995400 | -2.13721300 |
| H | 2.39016800 | 0.78119300 | 2.12633200 |
| H | 4.72944400 | 1.54211200 | 2.48521000 |
| H | 6.12860100 | 2.25650900 | 0.54619400 |
| I | 0.43254400 | 0.45721900 | -0.35338600 |
| C | 0.86670800 | -1.63495900 | -0.05237400 |
| C | 0.08127600 | -2.34208800 | 0.85335100 |
| C | 1.91087700 | -2.20497100 | -0.77781300 |
| C | 0.37594400 | -3.69966200 | 1.03753600 |
| H | -0.74233100 | -1.86257100 | 1.39130300 |
| C | 2.18367900 | -3.56259200 | -0.57311900 |
| H | 2.50668800 | -1.62248600 | -1.48438500 |
| C | 1.41868700 | -4.30683600 | 0.33121500 |
| H | -0.22613900 | -4.27997500 | 1.74301900 |
| H | 2.99573900 | -4.03580200 | -1.13342100 |
| H | 1.63528700 | -5.36844900 | 0.48279700 |
| O | -1.86071600 | -0.38537400 | -0.78925300 |
| S | -2.90568600 | -0.31124200 | 0.31632400 |
| O | -4.06634000 | -1.16371000 | 0.08483600 |
| O | -2.27803200 | -0.30558900 | 1.65523500 |
| C | -3.52512400 | 1.44378500 | 0.11074700 |
| F | -2.48396900 | 2.29823000 | 0.13818600 |
| F | -4.35839200 | 1.77182400 | 1.09431600 |
| F | -4.15252600 | 1.59638400 | -1.05317700 |

PhIPh-$K_2CO_3$

| | | | |
|---|---|---|---|
| C | -0.85986600 | 3.72713800 | 1.06341800 |
| C | -0.47872200 | 2.37951400 | 1.03728800 |
| C | -1.00080900 | 1.55919700 | 0.03570800 |
| C | -1.88052900 | 2.03858600 | -0.93410300 |
| C | -2.25065200 | 3.38882700 | -0.89280600 |
| C | -1.74237900 | 4.23107500 | 0.10144800 |
| H | -0.47332500 | 4.38108400 | 1.85150900 |
| H | 0.20933100 | 1.98056200 | 1.78614500 |
| H | -2.28718400 | 1.38202900 | -1.70746600 |
| H | -2.94326100 | 3.77849600 | -1.64486000 |
| H | -2.04143600 | 5.28286800 | 0.13139600 |
| I | -0.40181000 | -0.50943700 | 0.01349700 |
| C | -2.47160400 | -1.18689000 | 0.01501500 |
| C | -3.00100400 | -1.73349600 | -1.15963300 |
| C | -3.22899900 | -1.13856100 | 1.19132100 |
| C | -4.31217000 | -2.22644900 | -1.15685500 |
| H | -2.40452100 | -1.78197300 | -2.07615900 |
| C | -4.53891300 | -1.62922400 | 1.18222300 |
| H | -2.81090200 | -0.72090000 | 2.11222300 |
| C | -5.07955900 | -2.17217900 | 0.01055300 |
| H | -4.73203800 | -2.65409500 | -2.07245200 |
| H | -5.13770500 | -1.59073000 | 2.09735300 |
| H | -6.10328500 | -2.55793300 | 0.00965200 |
| C | 2.67513800 | -0.50231900 | 0.01316200 |
| O | 3.94097600 | -0.25046500 | -0.04876200 |
| O | 1.83911500 | 0.51283500 | -0.03014700 |
| O | 2.22179300 | -1.68669700 | 0.10627700 |
| K | 3.65744400 | 2.25470300 | -0.39932100 |
| K | 4.50179900 | -2.75554000 | 0.16447500 |

PhIPh-$KCO_3^-$

| | | | |
|---|---|---|---|
| C | 0.85866600 | 3.47073000 | 0.85631500 |
| C | 0.78575900 | 2.07240300 | 0.88740200 |
| C | -0.08115400 | 1.41647900 | 0.00915100 |
| C | -0.86651100 | 2.12931500 | -0.89779100 |
| C | -0.78355200 | 3.52747000 | -0.92161300 |
| C | 0.07662200 | 4.19920200 | -0.04698900 |
| H | 1.52554700 | 3.99277600 | 1.55074600 |
| H | 1.39955600 | 1.49563000 | 1.58323800 |
| H | -1.54804200 | 1.61098800 | -1.57754300 |
| H | -1.39936700 | 4.09134500 | -1.62962800 |

| | | | |
|---|---|---|---|
| H | 0.13375500 | 5.29199000 | -0.06567700 |
| I | -0.12533500 | -0.75548800 | 0.07528400 |
| C | -2.35330500 | -0.65625400 | 0.03157100 |
| C | -3.02693700 | -1.11061000 | -1.10891400 |
| C | -3.08620700 | -0.24769800 | 1.15369800 |
| C | -4.42805700 | -1.14280100 | -1.13379600 |
| H | -2.46407900 | -1.44682800 | -1.98709400 |
| C | -4.48499000 | -0.27602300 | 1.12674100 |
| H | -2.57090800 | 0.09702200 | 2.05686700 |
| C | -5.15727500 | -0.72330700 | -0.01720300 |
| H | -4.94908800 | -1.49890100 | -2.02876800 |
| H | -5.05320800 | 0.04738600 | 2.00534100 |
| H | -6.25147900 | -0.74910600 | -0.03495400 |
| C | 2.58052800 | -1.84643300 | 0.14658500 |
| O | 3.82666900 | -2.04485100 | 0.11051300 |
| O | 2.17957400 | -0.53023200 | 0.06407400 |
| O | 1.66980300 | -2.70342600 | 0.23334100 |
| K | 4.53737900 | 0.23483100 | -0.40194400 |

PhIPh-$CO_3^{2-}$

| | | | |
|---|---|---|---|
| C | 0.65218800 | 3.69847100 | -0.00008500 |
| C | 0.72874300 | 2.29861900 | -0.00000500 |
| C | -0.44541400 | 1.53970400 | 0.00008500 |
| C | -1.69735000 | 2.17185000 | 0.00008400 |
| C | -1.75953900 | 3.57122100 | 0.00004000 |
| C | -0.59017400 | 4.33962900 | -0.00004600 |
| H | 1.57689300 | 4.28725500 | -0.00018400 |
| H | 1.70660900 | 1.81275100 | 0.00000300 |
| H | -2.60210900 | 1.55839900 | 0.00005600 |
| H | -2.73923700 | 4.06255200 | 0.00004800 |
| H | -0.64676800 | 5.43388100 | -0.00010400 |
| I | -0.47627100 | -0.69144600 | 0.00000400 |
| C | 1.85759500 | -0.64444100 | 0.00001500 |
| C | 2.57474600 | -0.72544300 | 1.20349000 |
| C | 2.57474500 | -0.72537600 | -1.20346100 |
| C | 3.96970800 | -0.86439000 | 1.20935700 |
| H | 2.03933400 | -0.67932100 | 2.16063900 |
| C | 3.96972400 | -0.86431200 | -1.20935100 |
| H | 2.03933900 | -0.67923800 | -2.16061500 |
| C | 4.67133200 | -0.93237200 | -0.00000600 |
| H | 4.51190300 | -0.92304600 | 2.16093600 |
| H | 4.51189800 | -0.92287600 | -2.16094800 |
| H | 5.76155100 | -1.04339800 | 0.00000400 |
| C | -3.04408100 | -1.84548300 | -0.00002800 |

| | | | |
|---|---|---|---|
| O | -4.24175200 | -2.11432900 | -0.00003300 |
| O | -2.67105300 | -0.52010400 | 0.00009800 |
| O | -2.04849100 | -2.66136800 | -0.00013900 |

Phenyl-raidcal

| | | | |
|---|---|---|---|
| C | 0.00000000 | 1.21672800 | -0.63332400 |
| C | 0.00000000 | 1.22915400 | 0.77372900 |
| C | 0.00000000 | 0.00000000 | 1.40155800 |
| C | 0.00000000 | -1.22915400 | 0.77372900 |
| C | 0.00000000 | -1.21672800 | -0.63332400 |
| C | 0.00000000 | 0.00000000 | -1.32692300 |
| H | 0.00000000 | 2.16382600 | -1.18403000 |
| H | 0.00000000 | 2.17358700 | 1.32833300 |
| H | 0.00000000 | -2.17358700 | 1.32833300 |
| H | 0.00000000 | -2.16382600 | -1.18403000 |
| H | 0.00000000 | 0.00000000 | -2.42128000 |

decomposed $Ph_2I^+$-$CO_3^{2-}$

| | | | |
|---|---|---|---|
| C | -2.54927800 | 5.97508300 | -0.63889600 |
| C | -1.14511200 | 5.89439000 | -0.69463100 |
| C | -0.59019100 | 4.63730200 | -0.54189500 |
| C | -1.28457600 | 3.46204200 | -0.34115400 |
| C | -2.68921200 | 3.56726500 | -0.28899000 |
| C | -3.30933900 | 4.81497100 | -0.43710900 |
| H | -3.04367200 | 6.94762900 | -0.75392200 |
| H | -0.53642800 | 6.79272200 | -0.85173300 |
| H | -0.83068600 | 2.46858100 | -0.22107500 |
| H | -3.26068400 | 2.64431000 | -0.13054300 |
| H | -4.40192400 | 4.88487700 | -0.39484400 |
| I | 1.29810300 | -1.17608900 | 0.24515300 |
| C | 3.21085700 | -2.14646700 | 0.31685300 |
| C | 3.30411800 | -3.51552400 | 0.61334300 |
| C | 4.38898800 | -1.42528900 | 0.06677300 |
| C | 4.55121600 | -4.14924700 | 0.65863400 |
| H | 2.39372400 | -4.08995100 | 0.80987100 |
| C | 5.63495200 | -2.06133600 | 0.11262600 |
| H | 4.33130800 | -0.35757100 | -0.16593000 |
| C | 5.72281600 | -3.42612300 | 0.40856200 |
| H | 4.60544200 | -5.21880200 | 0.89165900 |
| H | 6.54453800 | -1.48283900 | -0.08533700 |
| H | 6.69818600 | -3.92312700 | 0.44411600 |
| C | -2.18831800 | -0.27958300 | 0.24119500 |
| O | -3.26392700 | 0.39867200 | 0.17629200 |
| O | -1.03864100 | 0.22989000 | 0.11838000 |

| | | | |
|---|---|---|---|
| O | -2.36819700 | -1.52839900 | 0.44245600 |

decomposed $Ph_2I^+$-$KCO_3^-$

| | | | |
|---|---|---|---|
| C | 5.35954600 | 0.45600500 | 1.34682500 |
| C | 4.11912500 | -0.14202000 | 1.09590900 |
| C | 3.37611400 | 0.25366500 | -0.02256400 |
| C | 3.86797900 | 1.23662300 | -0.88940300 |
| C | 5.10970600 | 1.82784800 | -0.62909300 |
| C | 5.85733400 | 1.44093900 | 0.48771200 |
| H | 5.93967000 | 0.14407400 | 2.22129100 |
| H | 3.73845500 | -0.91512800 | 1.76886900 |
| H | 3.29078700 | 1.54084100 | -1.76675500 |
| H | 5.49316200 | 2.59535500 | -1.30916200 |
| H | 6.82832400 | 1.90456500 | 0.68684300 |
| I | 1.48517800 | -0.65942100 | -0.41672300 |
| C | -3.88821800 | 2.78084900 | -0.35305100 |
| C | -3.66148000 | 1.90657100 | -1.39961800 |
| C | -4.56057200 | 2.48271700 | 0.81789200 |
| C | -4.15198800 | 0.59374700 | -1.24516900 |
| H | -3.12553600 | 2.20057400 | -2.30828300 |
| C | -5.04647500 | 1.16501400 | 0.94727100 |
| H | -4.72570800 | 3.22410000 | 1.60715300 |
| C | -4.83793000 | 0.23101300 | -0.07750100 |
| H | -3.97254300 | -0.14814400 | -2.02887000 |
| H | -5.58688400 | 0.87212500 | 1.85371300 |
| H | -5.19047500 | -0.79609100 | 0.04507300 |
| C | -1.93996000 | -2.33202100 | 0.10353100 |
| O | -2.44035400 | -1.97404000 | 1.20736900 |
| O | -1.34856600 | -1.53690100 | -0.69333100 |
| O | -2.04027800 | -3.56284800 | -0.23672100 |
| K | -1.70469500 | 0.50061500 | 0.94744500 |

PhI-TfO

| | | | |
|---|---|---|---|
| C | 4.37511600 | 1.35058300 | -0.75887700 |
| C | 3.17948600 | 1.86911900 | -1.26485700 |
| C | 2.07140500 | 1.94136000 | -0.41263700 |
| C | 2.11453600 | 1.49877900 | 0.91383600 |
| C | 3.31860700 | 0.97294500 | 1.39305800 |
| C | 4.44541100 | 0.90382900 | 0.56558200 |
| H | 5.25241800 | 1.28998000 | -1.40992100 |
| H | 3.11820500 | 2.20704300 | -2.30201300 |
| H | 1.22273200 | 1.53030800 | 1.54500100 |
| H | 3.36935700 | 0.61281000 | 2.42492000 |
| H | 5.38269700 | 0.49342800 | 0.95292200 |

| | | | |
|---|---|---|---|
| I | 0.26145700 | 2.74786000 | -1.15757000 |
| O | -0.94311100 | 0.46069400 | -0.72002900 |
| S | -1.63627600 | 0.28153400 | 0.64982800 |
| O | -1.89128100 | -1.14149000 | 0.86365700 |
| O | -1.03479200 | 1.09119100 | 1.71187300 |
| C | -3.30953700 | 1.06227000 | 0.33572900 |
| F | -3.14482700 | 2.31260800 | -0.11290200 |
| F | -4.00869800 | 1.10087800 | 1.46424300 |
| F | -3.98305700 | 0.36816500 | -0.57357600 |

PhI-$K_2CO_3$

| | | | |
|---|---|---|---|
| I | 0.84229100 | 0.41342300 | -0.00457800 |
| C | 2.94194300 | 0.08286400 | -0.00166300 |
| C | 3.61548000 | -0.10658600 | -1.21773100 |
| C | 3.63864300 | 0.05607300 | 1.21587600 |
| C | 4.99858500 | -0.31527700 | -1.20965500 |
| H | 3.06895000 | -0.08848300 | -2.16446900 |
| C | 5.02171600 | -0.15262200 | 1.20946100 |
| H | 3.11015600 | 0.19926600 | 2.16221400 |
| C | 5.70297200 | -0.33892800 | 0.00036700 |
| H | 5.52736400 | -0.46009600 | -2.15704600 |
| H | 5.56818300 | -0.17056700 | 2.15769200 |
| H | 6.78468700 | -0.50423200 | 0.00115300 |
| C | -2.47249200 | -0.03297600 | 0.00282200 |
| O | -3.71111000 | -0.34630700 | 0.00426100 |
| O | -1.64472500 | -1.03627700 | 0.02873000 |
| O | -2.04687400 | 1.15040300 | -0.02142100 |
| K | -3.46912900 | -3.00429700 | 0.03412900 |
| K | -4.43404000 | 2.25771000 | -0.02604100 |

PhI- $CO_3^{2-}$

| | | | |
|---|---|---|---|
| I | 0.33977800 | -0.00001600 | -0.21050800 |
| C | -1.80710500 | 0.00000000 | -0.00872300 |
| C | -2.52197300 | -1.20725900 | 0.05606000 |
| C | -2.52191300 | 1.20727400 | 0.05646000 |
| C | -3.91612900 | -1.20711100 | 0.18094100 |
| H | -1.98148400 | -2.15806600 | 0.00832000 |
| C | -3.91606900 | 1.20715500 | 0.18134200 |
| H | -1.98137500 | 2.15806900 | 0.00903500 |
| C | -4.62131400 | 0.00002900 | 0.24389300 |
| H | -4.45518100 | -2.16044500 | 0.22951300 |
| H | -4.45507300 | 2.16050000 | 0.23022800 |
| H | -5.71238300 | 0.00004100 | 0.34156300 |
| C | 3.84989100 | 0.00001200 | 0.18292600 |

| | | | |
|---|---|---|---|
| O | 5.08892300 | 0.00024700 | -0.12859700 |
| O | 2.93692600 | 0.00036800 | -0.68875600 |
| O | 3.63726800 | -0.00060000 | 1.43996000 |

PhI

| | | | |
|---|---|---|---|
| C | 2.65610000 | 1.20927900 | 0.00003500 |
| C | 1.25652300 | 1.21765900 | 0.00005900 |
| C | 0.56771100 | 0.00039200 | 0.00030600 |
| C | 1.25593600 | -1.21703300 | 0.00018500 |
| C | 2.65565300 | -1.20963100 | -0.00025100 |
| C | 3.35787500 | -0.00045400 | -0.00003400 |
| H | 3.19820900 | 2.16027800 | -0.00002500 |
| H | 0.71248400 | 2.16535600 | -0.00026300 |
| H | 0.71113700 | -2.16418600 | 0.00057800 |
| H | 3.19708800 | -2.16103500 | -0.00033400 |
| H | 4.45206200 | -0.00086700 | -0.00009000 |
| I | -1.56169400 | -0.00001500 | -0.00003100 |

Intermediate II

| | | | |
|---|---|---|---|
| C | -6.42162100 | 2.32710500 | -0.34543600 |
| C | -5.98387800 | 1.80060700 | -1.78213600 |
| C | -7.04814000 | 1.20284100 | 0.52065700 |
| H | -7.16069000 | 1.58981600 | 1.55040600 |
| H | -8.04481700 | 0.87491100 | 0.15609300 |
| H | -6.35335500 | 0.34871600 | 0.57646700 |
| C | -7.39351700 | 3.52353000 | -0.36913500 |
| H | -8.34653400 | 3.27998400 | -0.88275500 |
| H | -7.63001600 | 3.81889800 | 0.67110900 |
| H | -6.93626400 | 4.39745200 | -0.86326300 |
| C | -6.90461800 | 0.71536900 | -2.37723900 |
| H | -7.94764700 | 1.07061300 | -2.51190600 |
| H | -6.51601400 | 0.40695200 | -3.36800000 |
| H | -6.91190400 | -0.17793900 | -1.73238000 |
| C | -5.87186100 | 2.96173300 | -2.80602600 |
| H | -5.36923900 | 2.57604200 | -3.71319700 |
| H | -6.85248400 | 3.38907800 | -3.10328000 |
| H | -5.23798300 | 3.76152700 | -2.38824700 |
| O | -5.21228800 | 2.73398300 | 0.20684700 |
| O | -4.72117000 | 1.26497300 | -1.56322600 |
| B | -4.12610800 | 1.76690800 | -0.25064000 |
| C | -2.74407700 | 2.60116600 | -0.49288800 |
| H | -2.26670400 | 2.94736300 | 0.43642100 |
| C | -2.12569400 | 2.85427400 | -1.65977700 |
| H | -2.54660300 | 2.49858600 | -2.61482200 |

| | | | |
|---|---|---|---|
| H | -1.16698800 | 3.40563600 | -1.73596100 |
| O | -3.97761200 | 0.60962400 | 0.60930200 |
| C | -3.45250100 | 0.56954600 | 1.92417900 |
| O | -2.87822000 | 1.59560500 | 2.35648000 |
| O | -3.63651100 | -0.52329800 | 2.50164700 |

Intermediate III

| | | | |
|---|---|---|---|
| C | 2.64803800 | -0.96722300 | 0.53062500 |
| C | 1.56201200 | -1.73308600 | -0.34384400 |
| C | 3.76140900 | -0.34151200 | -0.34887800 |
| H | 4.37358700 | 0.31901300 | 0.29287100 |
| H | 4.42708800 | -1.10030800 | -0.81116600 |
| H | 3.30631300 | 0.29743200 | -1.12307500 |
| C | 3.31303800 | -1.82393300 | 1.62547800 |
| H | 3.85164100 | -2.69805300 | 1.20534200 |
| H | 4.04467500 | -1.20319300 | 2.17722800 |
| H | 2.56878100 | -2.18206400 | 2.35654000 |
| C | 2.13016300 | -2.46616600 | -1.57555300 |
| H | 2.87886500 | -3.23756400 | -1.29976500 |
| H | 1.30572100 | -2.96761800 | -2.11964800 |
| H | 2.59607400 | -1.74817400 | -2.26924600 |
| C | 0.76560400 | -2.75457300 | 0.50978600 |
| H | -0.10556000 | -3.10122400 | -0.07722900 |
| H | 1.36103200 | -3.64346600 | 0.80441900 |
| H | 0.38262400 | -2.26126000 | 1.41885000 |
| O | 1.89502500 | 0.03504700 | 1.13487200 |
| O | 0.71086500 | -0.71637600 | -0.76240700 |
| B | 0.85384700 | 0.53369000 | 0.15374400 |
| C | -0.52254600 | 0.85085900 | 0.92994900 |
| H | -0.67272400 | 0.59689000 | 1.99392900 |
| C | -1.71280700 | 1.48623300 | 0.26554100 |
| H | -1.40128900 | 1.85420400 | -0.73027500 |
| H | -2.07053800 | 2.38092200 | 0.81761900 |
| O | 1.31789100 | 1.59543000 | -0.71837800 |
| C | 1.32708600 | 2.96359200 | -0.36953800 |
| O | 0.68636100 | 3.29701500 | 0.65944700 |
| O | 1.97171100 | 3.69037500 | -1.15047100 |
| C | -2.66869600 | -0.70578300 | -0.58790400 |
| C | -2.87218500 | 0.51943700 | 0.08519400 |
| C | -4.16226900 | 0.78475000 | 0.57950700 |
| C | -5.21586100 | -0.12697700 | 0.41105800 |
| C | -5.00269200 | -1.32766500 | -0.27058700 |
| C | -3.71871100 | -1.60754100 | -0.77178700 |
| H | -1.65262500 | -0.93054300 | -0.93760100 |

| | | | |
|---|---|---|---|
| H | -4.33920100 | 1.73008300 | 1.10763800 |
| H | -6.20974900 | 0.10584800 | 0.81674700 |
| H | -5.82249200 | -2.04490300 | -0.40518400 |
| H | -3.53577900 | -2.54887000 | -1.30550100 |

Intermediate IV

| | | | |
|---|---|---|---|
| C | -2.87439700 | -0.90436100 | -0.29274300 |
| C | -2.51768200 | -0.45580300 | 1.22151500 |
| C | -4.21899400 | -0.32416700 | -0.80209800 |
| H | -4.34679900 | -0.63784600 | -1.85863200 |
| H | -5.10145800 | -0.68618000 | -0.23057600 |
| H | -4.14860300 | 0.77823100 | -0.77417700 |
| C | -2.88148000 | -2.43876600 | -0.50265900 |
| H | -3.66964400 | -2.94830500 | 0.09277200 |
| H | -3.08539500 | -2.63821800 | -1.57366500 |
| H | -1.89842000 | -2.88042600 | -0.26929100 |
| C | -3.65490500 | 0.33368400 | 1.91580800 |
| H | -4.59763700 | -0.24581900 | 2.01988900 |
| H | -3.31361700 | 0.61313400 | 2.93420100 |
| H | -3.82500100 | 1.26236100 | 1.34563700 |
| C | -2.09437700 | -1.64221900 | 2.12454000 |
| H | -1.78016400 | -1.23005800 | 3.10497600 |
| H | -2.91669500 | -2.36703400 | 2.30596400 |
| H | -1.22682600 | -2.16266500 | 1.68695700 |
| O | -1.85543900 | -0.36704600 | -1.07300600 |
| O | -1.43172300 | 0.40308200 | 1.09867300 |
| B | -0.94778800 | 0.53063300 | -0.30927300 |
| C | 0.61804900 | -0.06500900 | -0.41316900 |
| H | 0.94684700 | -0.09160200 | -1.46776100 |
| C | 1.55612700 | 0.83330800 | 0.40351700 |
| H | 1.25427700 | 0.74311800 | 1.46724400 |
| H | 1.30590900 | 1.87388700 | 0.11829400 |
| O | -0.89775500 | 1.92353400 | -0.77966200 |
| C | -1.88774800 | 2.90511100 | -0.78865700 |
| O | -1.49518500 | 4.03679500 | -1.15677200 |
| O | -3.06400900 | 2.59516300 | -0.44448200 |
| C | 3.89162200 | 1.54878800 | 1.07636100 |
| C | 3.06740300 | 0.67327900 | 0.33534000 |
| C | 3.70887400 | -0.30675700 | -0.45149700 |
| C | 5.10793300 | -0.38739100 | -0.48632700 |
| C | 5.91113200 | 0.49401600 | 0.24978400 |
| C | 5.28815500 | 1.47063900 | 1.03826600 |
| H | 3.41073200 | 2.31684200 | 1.69633600 |
| H | 3.09070800 | -1.02010600 | -1.02835900 |

| | | | |
|---|---|---|---|
| H | 5.57831300 | -1.16543700 | -1.10323200 |
| H | 7.00749400 | 0.42158300 | 0.21280200 |
| H | 5.89396200 | 2.17629500 | 1.62523000 |
| O | 0.66462600 | -1.41566000 | 0.10767800 |
| C | 1.08803300 | -2.52196400 | -0.64530900 |
| O | 0.73286900 | -3.61663800 | -0.16143100 |
| O | 1.78592900 | -2.31413700 | -1.67278300 |

TS1

| | | | |
|---|---|---|---|
| C | -6.54899300 | 1.79311600 | -0.09494000 |
| C | -6.45492700 | 1.89113100 | -1.69123600 |
| C | -6.99430100 | 0.38617800 | 0.38909000 |
| H | -6.86327900 | 0.34910100 | 1.48717600 |
| H | -8.05418000 | 0.14738300 | 0.15468300 |
| H | -6.33949500 | -0.38861300 | -0.04416700 |
| C | -7.45796100 | 2.86018000 | 0.55016900 |
| H | -8.50161400 | 2.81190600 | 0.17197900 |
| H | -7.48503400 | 2.69370700 | 1.64577900 |
| H | -7.03931800 | 3.86762400 | 0.38179200 |
| C | -7.33747300 | 0.86705800 | -2.44139500 |
| H | -8.41827400 | 1.00850100 | -2.23167400 |
| H | -7.18100100 | 0.99334300 | -3.53055600 |
| H | -7.05681100 | -0.16841100 | -2.18636100 |
| C | -6.79959800 | 3.30343500 | -2.22406400 |
| H | -6.56171700 | 3.32484200 | -3.30618400 |
| H | -7.87373300 | 3.55599000 | -2.09968900 |
| H | -6.16770400 | 4.05948600 | -1.72487500 |
| O | -5.24507600 | 2.00531200 | 0.33718300 |
| O | -5.11774400 | 1.59717000 | -1.96017500 |
| B | -4.26095000 | 1.85990300 | -0.75765600 |
| C | -3.31368200 | 3.20224500 | -0.87645000 |
| H | -2.69211700 | 3.28050700 | 0.03381200 |
| C | -2.42576700 | 3.18434700 | -2.10443700 |
| H | -2.95201900 | 2.90454400 | -3.03544500 |
| H | -1.89236600 | 1.74138900 | -1.87920600 |
| O | -3.49806300 | 0.56541100 | -0.45016100 |
| C | -2.42657300 | -0.02883300 | -0.94633400 |
| O | -1.62300900 | 0.66256100 | -1.73966100 |
| O | -2.17067600 | -1.20322300 | -0.66585900 |
| C | -0.63748400 | 4.12934400 | -3.58947700 |
| C | -1.32114100 | 4.06338000 | -2.32054200 |
| C | -0.76523100 | 4.92682500 | -1.30504000 |
| C | 0.37068600 | 5.69520800 | -1.53373300 |
| C | 1.04673700 | 5.70029000 | -2.77513500 |

| | | | |
|---|---|---|---|
| C | 0.49907500 | 4.90339400 | -3.80074200 |
| H | -1.03394800 | 3.52149200 | -4.41506800 |
| H | -1.30153800 | 5.01047200 | -0.35233300 |
| H | 0.73835600 | 6.34124400 | -0.72022400 |
| H | 1.94194200 | 6.31615400 | -2.94173600 |
| H | 0.97602100 | 4.89226900 | -4.79565000 |
| O | -4.20209400 | 4.36828900 | -0.85843800 |
| C | -4.26432700 | 5.24306000 | 0.24469200 |
| O | -5.31918700 | 5.92374000 | 0.26489100 |
| O | -3.29867900 | 5.27358600 | 1.03912300 |

Intermediate V

| | | | |
|---|---|---|---|
| C | -3.17449000 | -0.36742100 | -0.22001100 |
| C | -2.67208300 | 0.00174700 | 1.25530600 |
| C | -4.26921100 | 0.60525500 | -0.73528200 |
| H | -4.43105100 | 0.39485400 | -1.80937000 |
| H | -5.24169700 | 0.50270700 | -0.20725400 |
| H | -3.91671400 | 1.64750800 | -0.65586800 |
| C | -3.67128100 | -1.81962100 | -0.37253900 |
| H | -4.51352100 | -2.05319900 | 0.31326200 |
| H | -4.02655200 | -1.97041400 | -1.41165300 |
| H | -2.83702600 | -2.52339900 | -0.20644700 |
| C | -3.69892700 | 0.79647500 | 2.09320800 |
| H | -4.63275100 | 0.22268900 | 2.26772300 |
| H | -3.25098500 | 1.02971100 | 3.07881400 |
| H | -3.95433600 | 1.75530500 | 1.61200300 |
| C | -2.24397100 | -1.24304600 | 2.06972500 |
| H | -1.76822500 | -0.88865600 | 3.00558000 |
| H | -3.10377300 | -1.89122400 | 2.34083300 |
| H | -1.49013500 | -1.82164800 | 1.50627200 |
| O | -2.04429200 | -0.20473900 | -1.01430800 |
| O | -1.56342200 | 0.82036300 | 1.02788400 |
| B | -0.98903100 | 0.56541100 | -0.32932600 |
| C | 0.44167900 | -0.24916400 | -0.33123500 |
| H | 0.80691800 | -0.32738800 | -1.37095700 |
| C | 1.49671900 | 0.39305200 | 0.54180100 |
| H | 1.13607800 | 0.72450400 | 1.53147000 |
| H | 1.23379900 | 1.94261100 | -0.04885200 |
| O | -0.95002000 | 1.92682900 | -1.06511900 |
| C | -0.20949300 | 3.00832000 | -0.97756900 |
| O | 0.96850700 | 2.92645900 | -0.35569000 |
| O | -0.56767400 | 4.08097600 | -1.46521900 |
| C | 3.81448500 | 0.67318300 | 1.45748700 |
| C | 2.88891900 | 0.13331400 | 0.48333200 |

| | | | |
|---|---|---|---|
| C | 3.52553300 | -0.66143400 | -0.54816500 |
| C | 4.90381800 | -0.81765600 | -0.61446900 |
| C | 5.78493500 | -0.23218000 | 0.32594200 |
| C | 5.19171000 | 0.50621000 | 1.37195800 |
| H | 3.39569600 | 1.25620600 | 2.29031400 |
| H | 2.87960300 | -1.19409600 | -1.25543400 |
| H | 5.32009800 | -1.44402700 | -1.42057000 |
| H | 6.87377000 | -0.36830300 | 0.26133100 |
| H | 5.83221400 | 0.96187100 | 2.14696700 |
| O | 0.14775700 | -1.62429900 | 0.09451700 |
| C | 0.18018900 | -2.70731400 | -0.80742400 |
| O | -0.46348200 | -3.70255700 | -0.39135100 |
| O | 0.83544700 | -2.58565500 | -1.86495400 |

TS2

| | | | |
|---|---|---|---|
| C | -3.10712700 | -0.27740500 | -0.18036700 |
| C | -2.52998300 | 0.17423700 | 1.24833800 |
| C | -4.41334300 | 0.46720100 | -0.56055100 |
| H | -4.64152500 | 0.23407800 | -1.61765600 |
| H | -5.28579100 | 0.16800200 | 0.05807000 |
| H | -4.27511700 | 1.56007900 | -0.48654800 |
| C | -3.31793300 | -1.79707500 | -0.31664100 |
| H | -4.01913100 | -2.19264800 | 0.44947900 |
| H | -3.74935300 | -2.01168600 | -1.31507400 |
| H | -2.33475300 | -2.30779200 | -0.25900300 |
| C | -3.57506400 | 0.82708200 | 2.17863600 |
| H | -4.40051000 | 0.13038500 | 2.42823700 |
| H | -3.07759300 | 1.11996200 | 3.12392200 |
| H | -4.00266100 | 1.74118500 | 1.73220500 |
| C | -1.85135200 | -0.98743900 | 2.00747600 |
| H | -1.30340800 | -0.55728600 | 2.86966000 |
| H | -2.59747300 | -1.71107500 | 2.39773100 |
| H | -1.13407500 | -1.52517100 | 1.34981200 |
| O | -2.12184200 | 0.12118100 | -1.08995100 |
| O | -1.57799500 | 1.15567100 | 0.91992100 |
| B | -1.07516400 | 0.89317900 | -0.44490500 |
| C | 0.41507000 | 0.22159000 | -0.48591600 |
| H | 0.74277200 | -0.32914200 | -1.37505000 |
| C | 1.37088500 | 0.56557000 | 0.45604200 |
| H | 1.04176300 | 1.14995800 | 1.33201400 |
| H | 1.06807800 | 2.32931800 | -0.40850100 |
| O | -1.11500600 | 2.28822600 | -1.20557300 |
| C | -0.37734100 | 3.35800600 | -1.21152200 |
| O | 0.86535700 | 3.27100300 | -0.67104200 |

| | | | |
|---|---|---|---|
| O | -0.73051500 | 4.43220100 | -1.68541600 |
| C | 3.68513000 | 0.75507100 | 1.38966200 |
| C | 2.75762300 | 0.13152900 | 0.50704200 |
| C | 3.25285000 | -0.94494000 | -0.29466800 |
| C | 4.59584900 | -1.31187500 | -0.21939400 |
| C | 5.50814200 | -0.66785600 | 0.64244400 |
| C | 5.02665100 | 0.37116000 | 1.45595800 |
| H | 3.32618200 | 1.57331600 | 2.02967700 |
| H | 2.54800000 | -1.51746000 | -0.94361600 |
| H | 4.94119200 | -2.14987800 | -0.84268600 |
| H | 6.56138200 | -0.98108800 | 0.69278300 |
| H | 5.70688700 | 0.88968200 | 2.15005200 |
| O | -0.11563200 | -2.12861500 | -0.20893000 |
| C | 0.35057700 | -2.98290100 | -1.11590200 |
| O | -0.14791200 | -4.15288300 | -1.15092800 |
| O | 1.28181400 | -2.59056400 | -1.91953000 |

Intermediate VI

| | | | |
|---|---|---|---|
| C | -2.57328500 | -1.11454900 | -0.80977800 |
| C | -2.04387000 | -1.31568600 | 0.66909400 |
| C | -3.84699700 | -0.24103500 | -0.86134500 |
| H | -4.00708800 | 0.07616300 | -1.90631800 |
| H | -4.74464200 | -0.78879800 | -0.51742600 |
| H | -3.73945900 | 0.66923000 | -0.25100100 |
| C | -2.83771900 | -2.41742400 | -1.57668600 |
| H | -3.60132500 | -3.04216200 | -1.07575600 |
| H | -3.20510700 | -2.17494700 | -2.59032000 |
| H | -1.91403200 | -3.00641000 | -1.69196000 |
| C | -3.13318100 | -1.37664900 | 1.74719900 |
| H | -3.82109800 | -2.22580700 | 1.57655700 |
| H | -2.66702500 | -1.51064600 | 2.74055400 |
| H | -3.72443100 | -0.44794400 | 1.77888200 |
| C | -1.12944600 | -2.54751500 | 0.80257800 |
| H | -0.63358500 | -2.51646400 | 1.78918000 |
| H | -1.69251900 | -3.49602600 | 0.72694700 |
| H | -0.34347000 | -2.53561500 | 0.03018900 |
| O | -1.50288900 | -0.43725000 | -1.42896800 |
| O | -1.25182900 | -0.15001100 | 0.87194100 |
| B | -0.80918500 | 0.40012400 | -0.45279400 |
| C | 0.79295300 | 0.37203900 | -0.67208800 |
| H | 1.15241700 | 0.72848800 | -1.65187700 |
| C | 1.71010600 | -0.02966900 | 0.23194900 |
| H | 1.34188900 | -0.37504400 | 1.20838400 |
| H | -1.83038600 | 1.24359200 | 1.47896600 |

| | | | |
|---|---|---|---|
| O | -1.23612700 | 1.85140400 | -0.60058100 |
| C | -1.78325700 | 2.70749300 | 0.24777700 |
| O | -2.09978900 | 2.20539200 | 1.46525600 |
| O | -2.00796500 | 3.86541100 | -0.02281300 |
| C | 3.96822900 | -0.54195900 | 1.15758200 |
| C | 3.17673100 | -0.07006400 | 0.08779900 |
| C | 3.85943400 | 0.34072800 | -1.07938900 |
| C | 5.24991900 | 0.27955400 | -1.16859800 |
| C | 6.01552300 | -0.19406900 | -0.09407900 |
| C | 5.36155800 | -0.60446700 | 1.07212100 |
| H | 3.46650700 | -0.86532800 | 2.07640200 |
| H | 3.28307900 | 0.71630900 | -1.92954600 |
| H | 5.74582900 | 0.60749700 | -2.08940600 |
| H | 7.10750400 | -0.24020300 | -0.16663600 |
| H | 5.94147500 | -0.97644300 | 1.92464400 |

TS3

| | | | |
|---|---|---|---|
| C | -2.93699800 | -1.13662400 | -0.38741000 |
| C | -2.62649900 | -1.18288700 | 1.18606600 |
| C | -3.50429500 | 0.20473600 | -0.87573300 |
| H | -3.59152000 | 0.16466200 | -1.97680400 |
| H | -4.50961200 | 0.39641900 | -0.45871300 |
| H | -2.81553900 | 1.02533700 | -0.61165900 |
| C | -3.81662400 | -2.28543300 | -0.89647200 |
| H | -4.83103000 | -2.23773200 | -0.46090400 |
| H | -3.91223800 | -2.20343700 | -1.99338400 |
| H | -3.38298100 | -3.27319900 | -0.67225500 |
| C | -3.24085600 | -0.02512900 | 1.97829600 |
| H | -4.34487000 | -0.05141400 | 1.93097700 |
| H | -2.94028500 | -0.10639400 | 3.03821900 |
| H | -2.87299800 | 0.93573300 | 1.59046900 |
| C | -2.98055100 | -2.51879400 | 1.85754700 |
| H | -2.61183700 | -2.50002300 | 2.89795800 |
| H | -4.07097900 | -2.69317700 | 1.87900600 |
| H | -2.49729200 | -3.36955100 | 1.34877500 |
| O | -1.63551500 | -1.28906600 | -0.98143900 |
| O | -1.19037900 | -1.06690700 | 1.25169800 |
| B | -0.67324100 | -1.06038600 | -0.02856700 |
| C | 0.84849700 | -1.12241500 | -0.37093300 |
| H | 1.12857600 | -1.66471800 | -1.28750300 |
| C | 1.81594300 | -0.51261700 | 0.34580100 |
| H | 1.51352900 | 0.10554000 | 1.20055600 |
| H | -0.43515700 | 0.66665800 | 2.07079600 |
| O | -0.97168100 | 1.64574200 | 0.17314900 |

| | | | |
|---|---|---|---|
| C | -0.28186100 | 2.28045600 | 1.02246200 |
| O | 0.00637200 | 1.52560400 | 2.20495800 |
| O | 0.17541800 | 3.41638000 | 1.00341300 |
| C | 4.07130500 | 0.45877200 | 0.70380200 |
| C | 3.26261700 | -0.50557600 | 0.06683700 |
| C | 3.89320900 | -1.42113400 | -0.80157200 |
| C | 5.26881200 | -1.36817800 | -1.03009300 |
| C | 6.05566200 | -0.39828600 | -0.39497800 |
| C | 5.44799500 | 0.51552200 | 0.47298500 |
| H | 3.59377700 | 1.18113700 | 1.37371800 |
| H | 3.29396500 | -2.19266400 | -1.29430800 |
| H | 5.73461600 | -2.09303200 | -1.70725500 |
| H | 7.13543600 | -0.35796300 | -0.57510700 |
| H | 6.05050100 | 1.28135700 | 0.97341100 |

*trans*-phenylvinylboronate**(3e)**

| | | | |
|---|---|---|---|
| C | -2.91712000 | -0.75205100 | -0.33659700 |
| C | -2.48730200 | -0.90848900 | 1.18206400 |
| C | -3.41202300 | 0.65858800 | -0.68704600 |
| H | -3.49785300 | 0.74352700 | -1.78376900 |
| H | -4.40055000 | 0.86839900 | -0.24376300 |
| H | -2.70358200 | 1.43156300 | -0.34448300 |
| C | -3.91718300 | -1.79282700 | -0.83877300 |
| H | -4.87028000 | -1.72617800 | -0.28449300 |
| H | -4.12971400 | -1.61421700 | -1.90690000 |
| H | -3.52421500 | -2.81647300 | -0.74261200 |
| C | -3.20549300 | 0.02187600 | 2.15898500 |
| H | -4.29237700 | -0.17463500 | 2.16659800 |
| H | -2.82065300 | -0.14721700 | 3.17947900 |
| H | -3.04040400 | 1.08140500 | 1.91046700 |
| C | -2.56070900 | -2.35548400 | 1.69111300 |
| H | -2.05690200 | -2.41272000 | 2.67101900 |
| H | -3.60309500 | -2.69535500 | 1.81626800 |
| H | -2.04555400 | -3.05106100 | 1.00734500 |
| O | -1.66207300 | -0.94101700 | -1.03205400 |
| O | -1.08686100 | -0.54835000 | 1.14269600 |
| B | -0.63983500 | -0.70464100 | -0.14728800 |
| C | 0.85117600 | -0.62834500 | -0.56321000 |
| H | 1.09800500 | -0.71514700 | -1.62991400 |
| C | 1.84573000 | -0.46444200 | 0.33865200 |
| H | 1.56049700 | -0.38779400 | 1.39668600 |
| C | 4.16567800 | -0.23720500 | 1.19109500 |
| C | 3.29352100 | -0.37475200 | 0.09247400 |
| C | 3.86077700 | -0.42017300 | -1.19845200 |

| | | | |
|---|---|---|---|
| C | 5.24036700 | -0.33302600 | -1.37867900 |
| C | 6.09204100 | -0.19830000 | -0.27389300 |
| C | 5.54843100 | -0.15066100 | 1.01317700 |
| H | 3.74496100 | -0.19888000 | 2.20143200 |
| H | 3.21214000 | -0.52303600 | -2.07268300 |
| H | 5.65818100 | -0.36971200 | -2.38982100 |
| H | 7.17482800 | -0.13022600 | -0.41853800 |
| H | 6.20416900 | -0.04530000 | 1.88327700 |

$KCO_3^-$

| | | | |
|---|---|---|---|
| C | 0.00000000 | 1.10312900 | 0.00000000 |
| O | 1.12992900 | 0.43151700 | 0.00000000 |
| O | -1.12975100 | 0.39198500 | 0.00000000 |
| O | -0.05018700 | 2.35329700 | 0.00000000 |
| K | 0.02105700 | -1.68595600 | 0.00000000 |

$KCO_3\cdot$

| | | | |
|---|---|---|---|
| C | 1.10010500 | 0.00032600 | -0.00001600 |
| O | 0.50328500 | 1.11772700 | 0.00002500 |
| O | 0.50542200 | -1.11858700 | 0.00003200 |
| O | 2.38355200 | 0.00087700 | 0.00009400 |
| K | -1.77572100 | -0.00011000 | -0.00005900 |

$HCO_3^-$

| | | | |
|---|---|---|---|
| C | -0.16074700 | 0.06827400 | -0.00000400 |
| O | 0.10024200 | 1.28958000 | 0.00001700 |
| O | 1.02514900 | -0.76646100 | 0.00001700 |
| H | 1.73182700 | -0.09950200 | 0.00005400 |
| O | -1.22130900 | -0.56188700 | -0.00003800 |

$CO_3^{2-}$

| | | | |
|---|---|---|---|
| C | 0.00000000 | 0.00000000 | -0.00005800 |
| O | 0.00000000 | 0.00000000 | 1.30661800 |
| O | 0.00000000 | 1.13149900 | -0.65328700 |
| O | 0.00000000 | -1.13149900 | -0.65328700 |

Intermediate II-K

| | | | |
|---|---|---|---|
| C | -1.77463100 | -0.59256900 | 0.50973500 |
| C | -1.89204900 | 0.20298000 | -0.86058900 |
| C | -2.26280100 | -2.04973700 | 0.44011700 |
| H | -2.14639000 | -2.53270700 | 1.42990200 |
| H | -3.33187900 | -2.11309400 | 0.16090400 |
| H | -1.68301500 | -2.63447400 | -0.29468600 |
| C | -2.51099100 | 0.11770000 | 1.66634800 |

| | | | |
|---|---|---|---|
| H | -3.61201900 | 0.06849400 | 1.56764100 |
| H | -2.22548600 | -0.37074100 | 2.61649200 |
| H | -2.19918300 | 1.17213600 | 1.72906600 |
| C | -1.62180400 | -0.70008000 | -2.08809500 |
| H | -2.43939400 | -1.42061200 | -2.28557600 |
| H | -1.50684500 | -0.04888100 | -2.97217000 |
| H | -0.67235200 | -1.24677300 | -1.96696100 |
| C | -3.23698500 | 0.91751900 | -1.06632100 |
| H | -3.22516100 | 1.44482800 | -2.03766900 |
| H | -4.08710700 | 0.20802200 | -1.07036200 |
| H | -3.40779600 | 1.67353000 | -0.28328900 |
| O | -0.39092400 | -0.57134500 | 0.75873600 |
| O | -0.87856000 | 1.16177300 | -0.75831700 |
| B | 0.25600400 | 0.67623200 | 0.04564200 |
| C | 0.73920700 | 1.76710800 | 1.14436600 |
| H | 1.64343500 | 1.53682800 | 1.73767400 |
| C | 0.13485000 | 2.93206400 | 1.42153500 |
| H | -0.75231900 | 3.24933600 | 0.85390100 |
| H | 0.50316800 | 3.63569600 | 2.18625900 |
| O | 1.36202000 | 0.09222100 | -0.72262000 |
| C | 2.73990900 | 0.45014200 | -0.67284600 |
| O | 3.06033300 | 1.59851000 | -0.94403500 |
| O | 3.47587700 | -0.54147300 | -0.35064000 |
| K | 1.73390100 | -2.01005300 | 0.60895600 |

Intermediate III-K

| | | | |
|---|---|---|---|
| C | -2.02571800 | -1.38357200 | -0.41651900 |
| C | -1.83516600 | -0.28085700 | -1.54155600 |
| C | -1.81748300 | -2.82895200 | -0.89448300 |
| H | -1.97573900 | -3.52989100 | -0.05199100 |
| H | -2.52894900 | -3.10328200 | -1.69629800 |
| H | -0.79343000 | -2.98027500 | -1.27448600 |
| C | -3.40559300 | -1.27970900 | 0.26876400 |
| H | -4.23419400 | -1.61363500 | -0.38379500 |
| H | -3.39974800 | -1.91432300 | 1.17425700 |
| H | -3.59779700 | -0.24201500 | 0.58706900 |
| C | -0.70617800 | -0.64831200 | -2.53323400 |
| H | -0.98159800 | -1.48089000 | -3.20960500 |
| H | -0.48581900 | 0.24290900 | -3.14658700 |
| H | 0.21849400 | -0.90370100 | -1.99122200 |
| C | -3.10727000 | 0.04100100 | -2.34096200 |
| H | -2.87690800 | 0.81740500 | -3.09296600 |
| H | -3.49914100 | -0.84713500 | -2.87364400 |
| H | -3.89777900 | 0.44176400 | -1.68632100 |

| | | | |
|---|---|---|---|
| O | -1.02400600 | -1.04750900 | 0.51455300 |
| O | -1.46265600 | 0.85198400 | -0.80494500 |
| B | -0.69680900 | 0.46867800 | 0.39995200 |
| C | -1.21555800 | 1.24709800 | 1.71657900 |
| H | -1.64630700 | 0.68923100 | 2.56739200 |
| C | -1.38835700 | 2.74353600 | 1.80680800 |
| O | 0.77249900 | 0.50044800 | 0.20928800 |
| C | 1.70999500 | 1.27780800 | 0.91884600 |
| O | 1.61475600 | 2.50725700 | 0.87525100 |
| O | 2.57369300 | 0.57247300 | 1.52750400 |
| K | 1.22267200 | -1.50883900 | 1.68825000 |
| C | -1.08917900 | 3.30697200 | 3.18396000 |
| C | -2.11053300 | 3.56917900 | 4.11221100 |
| C | 0.24373900 | 3.54656500 | 3.57511100 |
| C | -1.82247200 | 4.05254200 | 5.39519800 |
| H | -3.15331300 | 3.39320400 | 3.82119400 |
| C | 0.53162600 | 4.03257600 | 4.85397800 |
| H | 1.04056500 | 3.34393700 | 2.84784000 |
| C | -0.49667200 | 4.28688300 | 5.77228700 |
| H | -2.63872900 | 4.25019500 | 6.10052200 |
| H | 1.57442500 | 4.21656300 | 5.13795800 |
| H | -0.26515400 | 4.66787800 | 6.77390100 |
| H | -0.72331500 | 3.21812100 | 1.06731800 |
| H | -2.43417900 | 3.01951200 | 1.53415300 |

Intermediate IV-K

| | | | |
|---|---|---|---|
| C | 2.93439600 | -0.65621500 | 0.87729500 |
| C | 2.23603200 | -1.73064000 | -0.05998400 |
| C | 4.04498100 | 0.12636200 | 0.13867700 |
| H | 4.36508200 | 0.97157200 | 0.77699200 |
| H | 4.93532100 | -0.49625000 | -0.08151400 |
| H | 3.64696100 | 0.55375300 | -0.79612100 |
| C | 3.49678200 | -1.22460200 | 2.19004500 |
| H | 4.26240000 | -2.00452700 | 2.00667900 |
| H | 3.97646700 | -0.41360000 | 2.77324900 |
| H | 2.69012400 | -1.65053100 | 2.80756300 |
| C | 3.18595900 | -2.39949700 | -1.07184700 |
| H | 4.00871700 | -2.95122800 | -0.57368300 |
| H | 2.61206900 | -3.12147400 | -1.68358600 |
| H | 3.61851200 | -1.65434500 | -1.75892500 |
| C | 1.52202100 | -2.83562300 | 0.75604100 |
| H | 0.90045600 | -3.42646100 | 0.05707800 |
| H | 2.23377700 | -3.52692300 | 1.25168400 |
| H | 0.84809600 | -2.40154500 | 1.51526800 |

| | | | |
|---|---|---|---|
| O | 1.87677100 | 0.21879400 | 1.17589300 |
| O | 1.29397500 | -0.97445400 | -0.76281000 |
| B | 0.91629100 | 0.26150100 | -0.00507000 |
| C | -0.65686800 | 0.11257500 | 0.56451600 |
| C | -1.66054400 | 0.86161000 | -0.33933900 |
| H | -1.41138600 | 0.62405900 | -1.38764700 |
| H | -1.46575700 | 1.94138800 | -0.20791500 |
| O | 1.16703700 | 1.38123400 | -0.91908600 |
| C | 1.25216500 | 2.73861300 | -0.67009700 |
| O | 0.92282000 | 3.13807800 | 0.49809300 |
| O | 1.64548800 | 3.44156100 | -1.61019200 |
| C | -3.88890900 | -0.15850400 | -1.02282700 |
| C | -3.11953800 | 0.56303500 | -0.09044200 |
| C | -3.76801200 | 0.98688300 | 1.08897900 |
| C | -5.11771100 | 0.70450300 | 1.31617900 |
| C | -5.86789600 | -0.00842500 | 0.37152400 |
| C | -5.24196600 | -0.44108200 | -0.80242900 |
| H | -3.40627300 | -0.50420500 | -1.94435800 |
| H | -3.18190500 | 1.52893800 | 1.83457000 |
| H | -5.59069600 | 1.04158500 | 2.24682800 |
| H | -6.92745700 | -0.22817600 | 0.55173500 |
| H | -5.81047400 | -1.00433500 | -1.55348900 |
| C | -0.71715900 | -0.24558500 | 3.04378300 |
| O | -0.46406200 | 0.41993800 | 4.08373800 |
| O | -0.86062700 | -1.46138600 | 2.88849000 |
| O | -0.84796000 | 0.61046900 | 1.91149500 |
| K | 1.01828500 | 2.14943200 | 2.72880700 |
| H | -0.92124200 | -0.95790400 | 0.58466600 |

TS1-K

| | | | |
|---|---|---|---|
| C | 2.82964700 | -0.99584200 | 0.54580000 |
| C | 1.93563700 | -2.29058800 | 0.71316000 |
| C | 3.82814900 | -1.12076600 | -0.62567400 |
| H | 4.27860900 | -0.12851300 | -0.81827100 |
| H | 4.64969400 | -1.83117400 | -0.40874600 |
| H | 3.30759200 | -1.43069600 | -1.54570400 |
| C | 3.58562200 | -0.57007500 | 1.81306300 |
| H | 4.26816500 | -1.36679100 | 2.16759500 |
| H | 4.20218100 | 0.32655900 | 1.60268600 |
| H | 2.88577300 | -0.31366300 | 2.62387300 |
| C | 2.66329300 | -3.61294200 | 0.42556800 |
| H | 3.52137700 | -3.76913700 | 1.10849600 |
| H | 1.95742100 | -4.45278200 | 0.56264900 |
| H | 3.02244100 | -3.64870400 | -0.61569500 |

| | | | |
|---|---|---|---|
| C | 1.28898200 | -2.36348100 | 2.11628300 |
| H | 0.50247400 | -3.13846400 | 2.09195300 |
| H | 2.01847400 | -2.62808000 | 2.90687900 |
| H | 0.80404400 | -1.40883800 | 2.38011400 |
| O | 1.86236700 | -0.01280300 | 0.23478800 |
| O | 0.93661100 | -2.10508100 | -0.25553100 |
| B | 0.69942600 | -0.67751800 | -0.47387100 |
| C | -0.74988800 | -0.13502200 | 0.05824000 |
| C | -1.87832600 | -0.34629800 | -0.87937300 |
| H | -1.62669800 | -0.55649900 | -1.92697900 |
| H | -0.16587500 | 1.55804300 | -1.34113900 |
| O | 0.92578300 | -0.45459000 | -1.99278300 |
| C | 1.06318200 | 0.67216700 | -2.63114900 |
| O | 0.46879000 | 1.77977900 | -2.07568800 |
| O | 1.69281400 | 0.81294100 | -3.66135600 |
| C | -4.24784000 | -0.77360600 | -1.51662300 |
| C | -3.24118100 | -0.42746300 | -0.53784000 |
| C | -3.75911900 | -0.19004300 | 0.79250600 |
| C | -5.11199000 | -0.30302700 | 1.08361900 |
| C | -6.06911200 | -0.64750400 | 0.10444700 |
| C | -5.59629100 | -0.87786000 | -1.20385900 |
| H | -3.91742400 | -0.95996100 | -2.54731300 |
| H | -3.06076300 | 0.11370400 | 1.57879000 |
| H | -5.44414100 | -0.10432200 | 2.11344200 |
| H | -7.13571400 | -0.72593200 | 0.34878800 |
| H | -6.30843100 | -1.14694800 | -1.99924000 |
| C | -0.53102800 | 1.76752000 | 1.76438700 |
| O | 0.00265400 | 2.91053600 | 1.88768100 |
| O | -0.97508300 | 1.01492800 | 2.63407900 |
| O | -0.56111400 | 1.34661900 | 0.40872800 |
| K | 1.78069800 | 2.62487800 | 0.16533600 |
| H | -0.95614800 | -0.58020400 | 1.04377000 |

Intermediate V-K

| | | | |
|---|---|---|---|
| C | 2.79868100 | -1.04445900 | 0.57167700 |
| C | 1.85251500 | -2.30993800 | 0.66489000 |
| C | 3.79335400 | -1.14335600 | -0.60597800 |
| H | 4.28742600 | -0.16231500 | -0.74071000 |
| H | 4.58293100 | -1.89961800 | -0.42961000 |
| H | 3.26350800 | -1.37574500 | -1.54323600 |
| C | 3.57494200 | -0.72563100 | 1.85853100 |
| H | 4.23244100 | -1.56509000 | 2.15730700 |
| H | 4.22097100 | 0.16103400 | 1.70058800 |
| H | 2.89071900 | -0.49798700 | 2.69136400 |

| | | | |
|---|---|---|---|
| C | 2.52215100 | -3.64070500 | 0.28749400 |
| H | 3.37489700 | -3.88052900 | 0.95275200 |
| H | 1.78003000 | -4.45577700 | 0.37204700 |
| H | 2.87559700 | -3.62240300 | -0.75591600 |
| C | 1.21193300 | -2.44826700 | 2.06548100 |
| H | 0.39535500 | -3.18856800 | 1.99877300 |
| H | 1.93298300 | -2.78486400 | 2.83609300 |
| H | 0.76623800 | -1.49206900 | 2.38541000 |
| O | 1.87305100 | -0.00438500 | 0.32205900 |
| O | 0.85225700 | -2.02453900 | -0.27464100 |
| B | 0.69736900 | -0.57866600 | -0.45173600 |
| C | -0.73827100 | 0.01613600 | 0.05412400 |
| C | -1.87287900 | -0.30945300 | -0.83880600 |
| H | -1.62186100 | -0.54666800 | -1.88110400 |
| H | 0.11775100 | 1.80236100 | -1.22786900 |
| O | 0.96743500 | -0.31981900 | -1.94879800 |
| C | 1.23384100 | 0.80351600 | -2.56289900 |
| O | 0.77450600 | 1.95271700 | -1.98639600 |
| O | 1.87593700 | 0.87837900 | -3.59350600 |
| C | -4.20033900 | -0.96267100 | -1.42017000 |
| C | -3.22247300 | -0.46956800 | -0.47522200 |
| C | -3.75443600 | -0.17854600 | 0.83884000 |
| C | -5.09146100 | -0.38606500 | 1.14980700 |
| C | -6.01806200 | -0.88235300 | 0.20697400 |
| C | -5.53350200 | -1.15979400 | -1.08776900 |
| H | -3.85844600 | -1.19173500 | -2.43822800 |
| H | -3.08478500 | 0.26021000 | 1.58500400 |
| H | -5.43769400 | -0.13779300 | 2.16406400 |
| H | -7.07277400 | -1.03557600 | 0.46688400 |
| H | -6.22341800 | -1.54219500 | -1.85566800 |
| C | -0.65563500 | 2.17092800 | 1.48850800 |
| O | -0.05813000 | 3.28647000 | 1.50767100 |
| O | -1.27137600 | 1.58908400 | 2.37849400 |
| O | -0.51314000 | 1.52686000 | 0.22141700 |
| K | 2.03743900 | 2.62947900 | 0.32699300 |
| H | -0.93344000 | -0.30637100 | 1.08890500 |

TS2-K

| | | | |
|---|---|---|---|
| C | 2.80046900 | -1.05091600 | 0.58777400 |
| C | 1.88227200 | -2.33850800 | 0.64162300 |
| C | 3.80353100 | -1.09791200 | -0.58698100 |
| H | 4.28333300 | -0.10536600 | -0.68917700 |
| H | 4.60454500 | -1.84650300 | -0.43033900 |
| H | 3.28089100 | -1.30800400 | -1.53343100 |

| | | | |
|---|---|---|---|
| C | 3.56492400 | -0.75129000 | 1.88697800 |
| H | 4.23756900 | -1.58493900 | 2.16762000 |
| H | 4.19497500 | 0.15156100 | 1.75601800 |
| H | 2.87207000 | -0.55823400 | 2.72131100 |
| C | 2.58218100 | -3.64387100 | 0.22919900 |
| H | 3.43947000 | -3.88452000 | 0.88873600 |
| H | 1.85766500 | -4.47675500 | 0.28936600 |
| H | 2.93572500 | -3.58861500 | -0.81284200 |
| C | 1.24147700 | -2.53140400 | 2.03602500 |
| H | 0.44283300 | -3.28850600 | 1.94534600 |
| H | 1.96690300 | -2.87135100 | 2.80139600 |
| H | 0.77131400 | -1.59479900 | 2.37798900 |
| O | 1.85549800 | -0.02566200 | 0.36324700 |
| O | 0.87983600 | -2.04841700 | -0.29001100 |
| B | 0.69471000 | -0.59603500 | -0.44297300 |
| C | -0.76999500 | -0.07751500 | 0.03917000 |
| C | -1.87184700 | -0.40077500 | -0.82537100 |
| H | -1.62936300 | -0.64220900 | -1.86935400 |
| H | 0.12334100 | 1.83019300 | -1.16376000 |
| O | 0.98141300 | -0.30919500 | -1.92489700 |
| C | 1.24627100 | 0.82536900 | -2.52604700 |
| O | 0.78252200 | 1.96302200 | -1.95146500 |
| O | 1.90169500 | 0.89969200 | -3.55156400 |
| C | -4.21378600 | -1.03483900 | -1.38760200 |
| C | -3.24013100 | -0.50873000 | -0.46593200 |
| C | -3.77305800 | -0.12278200 | 0.81694800 |
| C | -5.11649700 | -0.29167000 | 1.13142600 |
| C | -6.04211200 | -0.83377900 | 0.21485800 |
| C | -5.55519600 | -1.19262300 | -1.05626800 |
| H | -3.86791300 | -1.32921600 | -2.38751900 |
| H | -3.10213500 | 0.36209500 | 1.53382800 |
| H | -5.46669500 | 0.02852700 | 2.12396600 |
| H | -7.10112500 | -0.95544100 | 0.47547700 |
| H | -6.24548100 | -1.60814800 | -1.80662200 |
| C | -0.67161100 | 2.28520200 | 1.42130600 |
| O | -0.00984700 | 3.37676700 | 1.48749200 |
| O | -1.37085500 | 1.76924900 | 2.29807500 |
| O | -0.50516400 | 1.62152800 | 0.20104600 |
| K | 2.02992900 | 2.58565600 | 0.35382300 |
| H | -0.96227900 | -0.25667200 | 1.10620000 |

Intermediate VI-K

| | | | |
|---|---|---|---|
| C | 2.91500700 | -1.11107700 | 0.63710300 |
| C | 2.29353000 | -1.72733800 | -0.68572200 |
| C | 4.14731900 | -0.22322300 | 0.34348400 |
| H | 4.42084900 | 0.31576400 | 1.26977900 |
| H | 5.02663400 | -0.81078700 | 0.01261800 |
| H | 3.89420900 | 0.53605100 | -0.41340800 |
| C | 3.29474200 | -2.14568000 | 1.70969800 |
| H | 4.04940700 | -2.86650700 | 1.33851200 |
| H | 3.72968200 | -1.62949100 | 2.58780000 |
| H | 2.41198200 | -2.70875000 | 2.05478800 |
| C | 3.32609500 | -2.08705900 | -1.76927300 |
| H | 4.05381400 | -2.84714900 | -1.42061000 |
| H | 2.79885400 | -2.49563800 | -2.65188400 |
| H | 3.87447800 | -1.19071000 | -2.09901100 |
| C | 1.43607300 | -2.98188100 | -0.38860600 |
| H | 0.87511800 | -3.24301400 | -1.30433300 |
| H | 2.03922700 | -3.86141300 | -0.08732000 |
| H | 0.69941500 | -2.76270600 | 0.40180900 |
| O | 1.86261400 | -0.31205400 | 1.11590800 |
| O | 1.46597200 | -0.70910000 | -1.16464400 |
| B | 1.12124700 | 0.28326300 | -0.10810500 |
| C | -0.47527100 | 0.35429100 | 0.20920800 |
| C | -1.45287300 | -0.42875100 | -0.29666300 |
| H | -1.17234100 | -1.23212000 | -0.99488200 |
| H | -0.80190500 | 3.15516100 | 2.10723500 |
| O | 1.66484200 | 1.57739000 | -0.50752300 |
| C | 1.60287000 | 2.77214300 | 0.19643200 |
| O | 1.03204900 | 2.73471600 | 1.34449900 |
| O | 2.12770700 | 3.74717000 | -0.34260200 |
| C | -3.79094100 | -1.28105400 | -0.55539100 |
| C | -2.89711700 | -0.33598100 | -0.00457900 |
| C | -3.45087400 | 0.65956200 | 0.83628400 |
| C | -4.81413600 | 0.68325700 | 1.12828800 |
| C | -5.68405900 | -0.26980200 | 0.57621500 |
| C | -5.16133700 | -1.25041000 | -0.27447000 |
| H | -3.38967600 | -2.06029800 | -1.21516300 |
| H | -2.81605500 | 1.43739400 | 1.27348800 |
| H | -5.17894800 | 1.45852400 | 1.81064800 |
| H | -6.75585500 | -0.24855000 | 0.80853300 |
| H | -5.82449200 | -2.00326900 | -0.72043100 |
| C | -1.98802400 | 2.75338000 | 3.65825600 |
| O | -0.98823800 | 2.21849000 | 4.23193700 |
| O | -3.14221200 | 2.88323600 | 4.06806300 |
| O | -1.74997100 | 3.24789200 | 2.36418400 |

| | | | |
|---|---|---|---|
| K | 0.91837400 | 1.06148800 | 3.14415800 |
| H | -0.79800200 | 1.17028500 | 0.87059600 |

TS3-K

| | | | |
|---|---|---|---|
| C | 3.52448500 | 0.85661100 | -1.09883700 |
| C | 3.76156500 | 1.26161500 | 0.43896000 |
| C | 3.71327100 | -0.63844500 | -1.39274900 |
| H | 3.51035200 | -0.80662600 | -2.46812000 |
| H | 4.75120300 | -0.96094000 | -1.18890200 |
| H | 2.98533400 | -1.26661200 | -0.82454300 |
| C | 4.32411600 | 1.69363800 | -2.10860700 |
| H | 5.41054800 | 1.51523200 | -2.00740000 |
| H | 4.02316500 | 1.40010900 | -3.13010000 |
| H | 4.13344100 | 2.77467200 | -2.00193600 |
| C | 4.31812200 | 0.13003100 | 1.31065300 |
| H | 5.29889300 | -0.22088200 | 0.93633500 |
| H | 4.46107800 | 0.51479300 | 2.33818500 |
| H | 3.57892000 | -0.70151700 | 1.37583400 |
| C | 4.59040700 | 2.54289500 | 0.63191500 |
| H | 4.58729200 | 2.80299000 | 1.70515900 |
| H | 5.63980200 | 2.40496700 | 0.31265400 |
| H | 4.16381700 | 3.39887300 | 0.08090600 |
| O | 2.12860300 | 1.15499300 | -1.30943900 |
| O | 2.43643900 | 1.55586200 | 0.91400900 |
| B | 1.52737400 | 1.35904400 | -0.08850300 |
| C | -0.01417700 | 1.32744700 | 0.14194800 |
| C | -0.94467900 | 2.11783300 | -0.43208300 |
| H | -0.62190600 | 2.93419500 | -1.09898300 |
| H | -2.03809200 | -2.05118200 | 1.71208300 |
| O | 1.26893600 | -2.19526700 | -0.25691700 |
| C | 0.92454800 | -1.80822500 | 0.95346300 |
| O | -0.36326800 | -1.65304200 | 1.19590100 |
| O | 1.79473300 | -1.55531100 | 1.83221300 |
| C | -3.24507000 | 2.99145500 | -0.85666800 |
| C | -2.41083600 | 2.00846400 | -0.28411500 |
| C | -3.02886800 | 0.92789700 | 0.38429400 |
| C | -4.42089900 | 0.82255000 | 0.45867900 |
| C | -5.23000600 | 1.81383800 | -0.11667800 |
| C | -4.63806100 | 2.90165300 | -0.76935800 |
| H | -2.78513600 | 3.83783200 | -1.38263100 |
| H | -2.42985600 | 0.13593700 | 0.84486600 |
| H | -4.85469000 | -0.06396400 | 0.93991100 |
| H | -6.32210100 | 1.72981900 | -0.06426600 |
| H | -5.26370600 | 3.68099400 | -1.22348500 |

| | | | |
|---|---|---|---|
| C | -3.84100200 | -2.57169500 | 1.01647000 |
| O | -3.28145900 | -3.16675300 | 0.04333600 |
| O | -5.05624200 | -2.42225800 | 1.22134500 |
| O | -3.00161800 | -1.99483500 | 1.96059300 |
| K | -1.11514100 | -2.51778800 | -1.02328500 |
| H | -0.32852700 | 0.49466700 | 0.80227600 |

$HCO_3^-$-$KCO_3^-$

| | | | |
|---|---|---|---|
| H | -3.14679700 | 1.76944100 | 0.00126800 |
| O | 2.53081400 | 1.13648800 | -0.00107400 |
| C | 3.32569500 | 0.07827400 | 0.00006200 |
| O | 2.75771900 | -1.11763800 | 0.00090000 |
| O | 4.58191400 | 0.20401900 | 0.00034600 |
| C | -3.46147900 | -0.10549300 | -0.00001500 |
| O | -2.20119300 | -0.16884200 | -0.00009100 |
| O | -4.32158100 | -0.98224700 | -0.00061300 |
| O | -3.95776400 | 1.23375200 | 0.00091600 |
| K | 0.46538100 | -0.21317800 | -0.00024300 |
